# New Polyprenylated Acylphloroglucinol Derivatives and Xanthones From *Hypericum wilsonii*

**DOI:** 10.3389/fchem.2021.717904

**Published:** 2021-09-24

**Authors:** Ji Hao, Tongxi Zhou, Yuanren Ma, Jingtong Deng, Haitao Cheng, Qiang Wang, Qinxiong Lin, Xinzhou Yang, Hoyoung Choi

**Affiliations:** ^1^ School of Pharmaceutical Sciences, South-Central University for Nationalities, Wuhan, China; ^2^ College of Korean Medicine, Kyung Hee University, Seoul, South Korea

**Keywords:** polyprenylated acylphloroglucinol derivatives, xanthones, type 2 diabetes, *Hypericum wilsonii*, GLUT4

## Abstract

Four new polyprenylated acylphloroglucinol derivatives, hyperwilone A-D (**1–4**), and two new xanthones, wilsonxanthone A (**5**) and wilsonxanthone B (**6**), together with eight known compounds were isolated from the aerial parts of *Hypericum wilsonii*. Their structures were expounded by comprehensive analysis of the 1D and 2D NMR spectra and HRESIMS. The relative configurations and absolute configurations of **1-6** were determined by NMR calculations and comparing their experimental and computed ECD data. All compounds were evaluated for GLUT4 translocation effects in L6 myotubes. Compound **5** showed the strongest GLUT4 translocation effects with 2.57 folds at a concentration of 30 μg/ml.

## Introduction

Diabetes is a common chronic disease caused by the combined action of genetic and environmental factors. Type 2 diabetes (T2DM) featured with insulin resistance is the most prevalent type of diabetes in the population, accounting for around 90% of all diabetes cases (Zimmet et al., 2001). A large amount of human and financial resources have been invested globally in the fight against T2DM and its related complicating disease (Ha do et al., 2009). This problem is especially widespread in China due to the rapid improvement of people’s quality of life and lack of exercise (Yang et al., 2017a). Although current antidiabetic treatment strategies have proven to be quiet effective, there are still some questions about tolerability and mechanism-based side effects (Cramer et al., 2008). Therefore, the development of more safer and effective antidiabetic drugs is in line with the actual needs of people today. Glucose transporter 4 (GLUT4) has a crucial role in systemic glucose homeostasis, which is one of the most potential target in the development of an antidiabetic drug (Leto and Saltiel, 2012). An increasing body of evidence suggests the enhancement of GLUT4’s translocation can revive insulin resistance; therefore, it hopefully leads to the exploitation of the new antidiabetic medicine (Zhang et al., 2007; Naresh et al., 2012).

The contribution of natural products (NP) to the development of antidiabetic drugs cannot be underestimated (Kazmi et al., 2018). Well-known examples include metformin derived from galegine, the main chemical component of European goat’s rue *Galega officinalis* L., for use in T2DM and dyslipidemias, as well as non-alcoholic fatty liver disease (Hundal and Inzucchi, 2003), and dapagliflozin derived from phlorizin, the main chemical constituent from roots of apple tree *Malus pumila* Mill., which is the first approved SGLT2 inhibitor for the treatment of T2DM, being an important option in the treatment of diabetes (Dhillon, 2019). Overall, natural products show great potential for the fight against T2DM.

The plants belonging to the genus *Hypericum* (Hypericaceae) are distributed widely in the temperate and subtropical regions of the northern hemisphere and have been used by folks as a traditional medicine. Polyprenylated acylphloroglucinol derivatives and polyprenylated xanthones with interesting structural characteristics, which catch the attention of chemists, have been isolated from *Hypericum* genus (Yang et al., 2015; Xu et al., 2016), and they have various biological activities, such as antidepressant (Verotta, 2003), antimicrobial (Oya et al., 2015), antitumor (Zhang et al., 2014), and hepatoprotective activities (Zhen et al., 2019). Especially studies have shown that ethyl acetate extract of *H. perforatum* has an antihyperglycemic effect in rats (Arokiyaraj et al., 2011), *H. attenuatum* regulates dyslipidemia and improves hyperglycemic status (Lv et al., 2019), and *H. laricifolium* has a good inhibitory effect on *α*-glucosidase (Quispe et al., 2017). Thus, there is great potential to discover new therapeutics for diabetes from this genus. *Hypericum wilsonii* N. Robson, which also belongs to the *Hypericum* genus and has been found to contain 1,2-seco-homoadamantane–type polycyclic polyprenylated acylphloroglucinols (PPAPs) (Xie et al., 2020a), is a kind of shrub, growing on hillsides, under forests, or grasslands, at an altitude of 1,000–1750 m. It is mainly distributed in Hubei and Sichuan provinces as well as Chongqing city of China. When we studied the antidiabetic active ingredients of *Hypericum wilsonii in vitro*, we found that its petroleum ether extract (HW-PE) showed good GLUT4 translocation activity. Bioassay-guided phytochemical investigation on the active HW-PE led to the separation of six new compounds, hyperwilone A (**1**), hyperwilone B (**2**), hyperwilone C (**3**), hyperwilone D (**4**), wilsonxanthone A (**5**), and wilsonxanthone B (**6**), along with **7** known polyprenylated acylphloroglucinol derivatives and a xanthone. Here, we describe the structure elucidation process of the new compounds in detail, the GLUT4 translocation activity in L6 cells of compounds **1–14**, and the effects of compound **5** *in vitro*.

## Material and Methods

### General Experimental Procedures

The ^1^H (600 MHz), ^13^C (150 MHz), and 2D (^1^H-^1^H COSY, HSQC, HMBC, and ROESY) NMR spectra were recorded on a Bruker Ascend IIITM 600 MHz NMR spectrometer (Bruker Corporation, Fallanden, Switzerland). The HR-ESI-MS data were obtained in the positive ion mode on a UHPLC system and the Q Exactive HF Mass Spectrometer (Thermo Fisher Scientific, United States). The UV spectra were recorded using a UH5300 ultraviolet–visible spectrophotometer (Hitachi, Japan). The IR spectra were measured on a Fourier transform infrared spectrophotometer (Shimadzu, Japan). Fluorescence was measured on a fluorescence microplate reader (Thermo Fisher Scientific, San Jose, CA, United States). Optical rotation was measured using an Autopol IV-T automatic polarimeter (Rudolph Research Analytical, United States). ECD spectra were recorded with a Chirascan Plus circular dichroism spectrometer (Applied Photophysics Ltd., London, United Kingdom). Semi-preparative HPLC was carried out on a Waters 2535 HPLC fitted with a 2998 Photodiode Array Detector and a 2707 Autosampler (Waters, United States). Separations were performed on a COSMOSIL PFP, Chloster, and C18 column (5 μm, 10 × 250 mm) (COSMOSIL, Japan). All solvents applied to chromatography were HPLC grade, and all other chemicals were analytical reagent grade. HPLC-grade acetonitrile was purchased from Merck Chemical Company (Darmstadt, Germany). Silica gel (300–400 mesh, Qingdao Marine Chemical Group Corporation, Qingdao, China), silica gel (H, Yantai Jiangyou Silica Gel Development Corporation, Yantai, China), and macroporous resin HP 20 (Mitsubishi, Japan) were used for column chromatography (CC). The analytical TLC plates were HSGF254 SiO_2_ from Yantai Jiangyou Silica Gel Development Co., Ltd., China.

### Plant Material

*Hypericum wilsonii* N. Robson was collected from Chongqing city, China, in August 2018. The identification of plant was done by Professor Dingrong Wan of School of Pharmaceutical Sciences, South-Central University for Nationalities (SCUN), Wuhan, China. A voucher specimen (SC0758) is deposited in the School of Pharmaceutical Sciences, SCUN, Wuhan, China.

### Extraction and Isolation

Air-dried aerial parts of *H wilsonii* N. Robson (21 kg) were broken to pieces and extracted entirely by immersion at room temperature with 80% ethanol (4 × 20 L, 3 days each). The extracting solution was evaporated in vacuo to be concentrated to yield 1947 g of fluid extract. The fluid extract was mixed to suspension by water (1:5), and the suspension was then extracted with petroleum ether (4 × 10L), ethyl acetate (4 × 10 L), and *n*-BuOH (4 × 10 L), respectively. The extracting solution was evaporated in vacuo to yield petroleum ether extract (452 g), ethyl acetate extract (674 g), and *n*-BuOH extract (712 g), respectively. The petroleum ether extract (430 g) was subjected to HP 20 macroporous resin column chromatography (4,500 g) eluted with 20, 30, 40, 50, 60, 65, 70, 75, 80, 82, 85, 90, and 95% aq. ethanol in a gradient way to divide into nine fractions (Fr. A-I). The Fr. G (115 g) was separated by column chromatography (silica gel 300–400 mesh) and eluted with PE-EtOAc (1:0 to 0:1 v/v), which afforded ten fractions, Ga-Gj. Fr. Ge was further separated on silica gel (10–40 μm) eluted with PE-CH_2_Cl_2_ (1:1 v/v) to obtain Ge1-Ge11.

Fr. Ge3 (6.9 g) was purified using semi-preparative HPLC (H_2_O-MeCN, 16:84 to 5:95, v/v, 40 min) to give compounds 8 (1.34 g), 9 (403.0 mg), 10 (354.2 mg), and other eight fractions. Fr. Ge3-6 (28.1 mg) was separated by HPLC and eluted with H_2_O-MeCN (32:68 to 29:71, v/v, 20 min) to afford 1 fraction (1.4 mg). Similarly, Fr. Ge3-7 (35.6 mg) was purified by HPLC chromatography (H_2_O-MeCN, 33:67 to 31:69, v/v, 20min) to afford 6 (1.2 mg) and 11 (5.4 mg) fractions. Fr. Ge3-8 (233.2mg) was separated by HPLC and eluted with H_2_O-MeCN (20:80 to 9:91, v/v, 20min) to give 5 (3.8 mg) and other six fractions. Subsequently, Ge3-8-2 (9.7 mg) was purified using semi-preparative HPLC (H_2_O-MeCN, 38:62) to give 12 (1.6 mg) fractions, and Ge3-8-4 (26.7 mg) was separated by semi-preparative HPLC (H_2_O-MeCN, 20:80 to 17:83, v/v, 20 min) to give 13 (17.2 mg) fractions. Fr. Ge3-9 (70.6 mg) was separated by HPLC and eluted with H_2_O-MeCN (23:77 to 15:85, v/v, 20 min) to give 14 (5.6 mg) fractions. Fr. Ge3-10 (161.5 mg) was separated by preparative TLC with PE-EtOAc (5:1, 0.1% formic acid) to give four fractions. Subsequently, Ge3-10-4 (22.7mg) was purified by semi-preparative HPLC (H_2_O-MeCN, 31:69 to 29:71, v/v, 20 min) to afford 2 (1.5 mg) fractions. Fr. Ge7 (1.06 g) was purified using preparative HPLC (H_2_O-MeCN, 10:90 v/v) to give ten fractions. Fr. Ge7-8 (45.6 mg) was purified using semi-preparative HPLC (H_2_O-MeCN, 3:97 to 1:99, v/v, 20 min) to afford 4 (2.3 mg) fractions.

Fr. Gd was then separated on silica gel (10–40 μm) and eluted with PE-CH_2_Cl_2_ (3.5:1 to 1:1, v/v) to obtain Gd1-Gd9. Fr. Gd7 (730 mg) was purified using preparative HPLC (H_2_O-MeCN, 5:95 to 0:100, v/v, 25 min) to give twelve fractions. Fr. Gd7-3 (32.5 mg) was purified using preparative HPLC (H_2_O-MeCN, 20:80, v/v) to afford 3 (2.6 mg) and 7 (14.3 mg) fractions. The purity of compounds 1–14 is at the range of 94.8–98.7%, respectively, by HPLC-PDA analysis.

### Compound Characterization

*Hyperwilone A* (**1**) colorless oil. [*α*] 20D +39.3 (*c* 0.65, CHCl_3_); UV (CHCl_3_) *λ*_max_ (log *ε*) 245 (0.71) nm; IR *ν*_max_ 1,219, 772 cm^−1^; ECD (*c* 0.5, MeOH) λ_max_(Δ*ε*) 210 (0.12), 222 (2.42), 229 (1.18), 233 (1.38), 248 (−1.89), 269 (0.61), and 317 (−1.16) nm; ^1^H and ^13^C and 2D NMR spectroscopic data (see Tables 1, 2; Supplementary Figures S1–7); HRESIMS *m/z* 531.2742 [M + H]^+^ (calcd for C_33_H_39_O_6_, 531.2747).

**TABLE 1:** ^1^H NMR (600 MHz) data of compounds **1-4** in CDCl_3_ (δ in ppm, *J* in Hz).

No	1	2	3	4
4	—	—	—	2.12 m
5	—	—	—	1.42 m
6	2.65 m	2.50 dd (14.3, 6.0)	2.37 dd (14.8, 6.6)	—
		1.80 d (14.3)	2.10 d (14.8)	
7	1.97 m	1.88 m	1.99 m	—
11	—	1.70 m	1.88 m	2.14 m
12/12′	7.12 d (8.2)	1.01 d (6.4)	0.99 d (6.6)	1.03 d (6.5)
13/13′	7.27 t (8.2)	2.00 d (13.7, 7.5, 2.2)	1.02 d (6.6)	1.13 d (6.5)
		1.31 m		
14	7.44 t (8.2)	0.82 t (7.5)	—	—
15	2.85 dd (15.7, 5.9)	3.18 d (7.2)	2.64 t (13.1)	2.50 m
	2.64 m		2.43 dd (13.1, 12.9)	
16	6.29 t (5.9)	4.51 t (7.2)	1.79 m	5.06 t (6.3)
18	9.30 s	1.62 s	1.34 s	1.71 s
19	1.83 s	1.52 s	1.40 s	1.99 m
20	2.58 d (7.2)	2.54 d (7.3)	2.52 t (7.0)	2.21 m
				1.82 m
21	5.18 t (7.2)	5.19 d (7.3)	5.23 d (7.0)	4.94 t (6.9)
23	1.71 s	1.69 s	1.73 s	1.68 s
24	1.67 s	1.71 s	1.65 s	1.56 s
25	2.70 m	1.91 m	1.90 m	2.96 dd (15.0, 7.1)
			1.63 m	2.86 dd (15.0, 10.8)
26	2.66 m	1.84 m	1.83 m	4.75 dd (10.8, 7.1)
28	1.28 s	1.40 s	1.11 s	1.30 s
29	1.39 s	1.34 s	1.04 s	1.19 s
30	1.52 s	1.23 s	1.25 s	1.21 s
31	1.45 s	1.27 s	1.26 s	1.33 s
32	—	—	—	2.08 m
				2.00 m
33	—	—	—	5.06 t (6.3)
35	—	—	—	1.66 s
36	—	—	—	1.58 s

**TABLE 2:** ^13^C NMR (150 MHz) data of compounds 1-4 in CDCl_3_ (δ in ppm).

No	1	2	3	4
1	81.9	87.6	85.5	81.3
2	200.8	206.3	202.9	207.0
3	70.6	71.1	70.9	52.7
4	202.6	208.5	203.2	38.0
5	69.2	68.1	67.3	48.0
6	40.3	38.5	35.1	47.7
7	44.5	43.6	42.1	177.7
8	56.1	47.7	47.3	119.6
9	201.3	205.5	204.8	187.6
10	192.3	208.7	207.8	209.2
11	134.5	50.3	43.0	42.0
12/12′	129.1	17.9	20.9	21.9
13/13′	128.2	26.8	20.4	20.7
14	133.0	11.8	—	—
15	27.5	34.3	30.1	29.7
16	147.9	119.8	59.4	119.5
17	141.6	136.5	73.0	138.8
18	194.7	18.3	31.3	16.7
19	9.7	26.3	30.6	40.1
20	27.7	29.0	29.1	29.7
21	117.7	119.3	118.9	124.9
22	136.1	135.2	135.5	132.4
23	26.2	18.2	26.2	25.9
24	18.3	26.2	18.2	18.0
25	51.8	29.7	22.6	27.6
26	60.5	49.7	56.7	93.2
27	61.2	76.1	46.6	71.9
28	19.8	26.8	29.7	26.4
29	24.5	33.2	17.1	23.6
30	23.6	24.9	22.5	26.6
31	22.7	22.8	25.1	22.4
32	—	—	—	26.6
33	—	—	—	123.9
34	—	—	—	131.9
35	—	—	—	25.8
36	—	—	—	17.8

*Hyperwilone B* (**2**) colorless oil. [*α*] 20D +51.1 (*c* 0.50, CHCl_3_); UV (CHCl_3_) *λ*_max_ (log *ε*) 245 (0.76) nm; IR *ν*_max_ 2957, 2924, 2853, 1,458, 1,377 cm^−1^; ^1^H, ^13^C and 2D NMR spectroscopic data (see Tables 1, 2; Supplementary Figures S12–18); HRESIMS *m/z* 499.3416 [M + H]^+^ (calcd for C_31_H_47_O_5_, 499.3423).

*Hyperwilone C* (**3**) colorless oil. [*α*] 20D +47.3 (*c* 0.50, MeOH); UV (CHCl_3_) *λ*_max_ (log *ε*) 265 (0.83) nm; IR *ν*_max_ 2965, 2928, 1726, 1,686, 760 cm^−1^; ECD (*c* 0.5, MeOH) λ_max_ (Δ*ε*) 237 (−1.08), 273 (2.35), 305 (−1.63), 341 (0.53) nm; ^1^H, ^13^C and 2D NMR spectroscopic data (see Tables 1, 2; Supplementary Figures S22–28); HRESIMS *m/z* 485.3256 [M + H]^+^ (calcd for C_30_H_45_O_5_, 485.3267).

*Hyperwilone D* (**4**) colorless oil. [*α*] 20D +50.0 (*c* 0.74, CHCl_3_); UV (CHCl_3_) *λ*_max_ (log *ε*) 270 (0.79) nm; IR *ν*_max_ 2926, 1730, 1,622, 1,219, 772 cm^−1^; ECD (*c* 0.5, MeOH) λ_max_ (Δ*ε*) 247 (4.57), 279 (−7.08) nm, 303 (10.97), 331 (−0.81) nm; ^1^H, ^13^C and 2D NMR spectroscopic data (see Tables 1, 2; Supplementary Figures S33–39); HRESIMS *m/z* 553.3886 [M + H]^+^ (calcd for C_35_H_53_O_5_, 553.3893).

*Wilsonxanthone A* (**5**) yellow powder. [*α*] 20D −10.1 (*c* 0.74, CHCl_3_); UV (CHCl_3_) *λ*_max_ (log *ε*) 245 (1.23) nm; IR *ν*_max_ 2926, 1,651, 1,581, 1,219, 772 cm^−1^; ECD (*c* 0.5, MeOH) λ_max_ (Δ*ε*) 259 (−0.23), 274 (−0.09), 305 (−0.25), 349 (0.09) nm; ^1^H, ^13^C and 2D NMR spectroscopic data (see Table 3; Supplementary Figures S44–50); HRESIMS *m/z* 381.1696 [M + H]^+^ (calcd for C_23_H_25_O_5_, 381.1702).

**TABLE 3:** ^1^H NMR (600 MHz) data and ^13^C NMR (150 MHz) data of compounds 5 and 6 in CDCl_3_ (δ in ppm, *J* in Hz).

No	5		6	
	δ_H_	δ_C_	δ_H_	δ_C_
1	—	161.3	—	161.3
2	6.28 s	99.8	6.35 s	99.6
3	—	161.5	—	162.1
4	—	100.1	—	100.8
4a	—	153.9	—	153.8
4b	—	144.3	—	144.3
5	—	144.4	—	144.4
6	7.33 dd (7.9, 1.2)	120.0	7.34 dd (7.9, 1.5)	120.2
7	7.27 t (7.9)	124.2	7.28 t (7.9)	124.4
8	7.78 dd (7.9, 1.2)	117.2	7.80 dd (7.9, 1.5)	117.2
8a	—	121.4	—	121.4
9	—	180.8	—	180.8
9a	—	103.6	—	103.8
1′	2.98 dd (16.3, 5.1)	21.9	3.03 dd (16.0, 5.3)	24.7
	2.44 dd (16.3, 9.3)		2.86 dd (16.0, 11.1)	
2′	1.86 m	40.8	2.54 ddd (11.1, 9.6, 5.3)	44.2
3′	—	79.9	—	144.6
4′	1.27 s	21.4	4.95 s	113.8
			4.89 s	
5′	1.48 s	27.6	1.78 s	20.6
1′′	2.33 m	29.3	4.74 dd (9.6, 9.2)	76.3
	1.90 m			
2′′	5.21 t (7.5)	122.1	5.29 d (9.2)	122.3
3′′	—	133.9	—	140.3
4′′	1.75 s	26.0	1.83 d (0.9)	26.1
5′′	1.61 s	18.1	1.79 d (0.9)	18.8

*Wilsonxanthone B* (**6**) yellow powder. [*α*] 20D +29.3 (*c* 0.55, CHCl_3_); UV (CHCl_3_) *λ*_max_ (log *ε*) 245 (0.84) nm; IR *ν*_max_ 2924, 1,219, 772 cm^−1^; ECD (*c* 0.5, MeOH) λ_max_ (Δ*ε*) 263 (1.81), 360 (−0.11) nm; ^1^H, ^13^C and 2D NMR spectroscopic data (see Table 3; Supplementary Figures S55–61); HRESIMS *m/z* 379.1539 [M + H]^+^ (calcd for C_23_H_23_O_5_, 379.1545).

### Computational ECD Details

The first step, random searching was used for conformational analyses through the MMFF force field in the Spartan’14. Afterward, the generated conformers were optimized at the B3LYP/6-31G(d) level in Gaussian 09 software with density functional theory (DFT). Time-dependent density functional theory (TD-DFT) method was chosen to calculate the conformers with a Boltzmann population of over 1% at the B3LYP/6–311+G(d, p) level, and SCRF/PCM method was used to evaluate the solvent effects of the MeOH solution. Finally, the Boltzmann-averaged ECD spectra were produced by the SpecDis 1.62 (Bruhn et al., 2013) using a Gaussian band shape with a 0.30 eV exponential half-width. The absolute configurations of 4–6 were resolved by comparing the experiment spectra with the calculated ECD spectra.

### NMR Calculations

The conformational analyses process implemented in Spartan’14 was used to search the conformation by using MMFF force field. Gaussian 09 program was used to optimize the geometric structure by DFT at B3LYP/6-31G(d) level, so as to obtain stable conformational isomers. The ^13^C NMR chemical shifts were calculated using the PCM solvent continuum model at mPW1PW91/6-311G(d,p) concentration using gauge-independent atomic orbitals (GIAO) (Grimblat et al., 2015). According to the Boltzmann distribution theory and its relative Gibbs free energy, the average value of the NMR calculated data of the isomers of 1 and 2 is taken. The ^13^C NMR chemical shifts of TMS were used as reference by calculating at the same theoretical level. The experimental and computational data of isomers were analyzed by the improved probabilistic DP4+ method (Lodewyk et al., 2012). The higher DP4+ probability score of compounds 1 and 2 indicates that its configuration is correct.

### Cell Culture

Rat skeletal muscle L6 cells, obtained from Wuhan Procell Life Science and Technology Co., Ltd, were cultured in complete media containing 1% antibiotics (100 U/mL penicillin and 100 μg/ml streptomycin) (Hyclone, United States), 10% FBS (FBS, Hyclone, United States), and 89% α-MEM (Gibco, United States) at 37°C and in the presence of 5% CO2. The medium was replenished with fresh medium containing 2% FBS when cells was subcultured at the density of 60%, and the medium was replaced every 48 h until the seventh day.

### Assay of GLUT4 Translocation

According to the manufacturer’s protocol, we used Lipofectamine 2000 to establish an L6 myotube stably overexpressing IRAP-mOrange. IRAP and GLUT4 are two colocalization proteins that exist on GLUT4 storage vesicles (GSV). IRAP can successfully act as a reporter molecule to reflect the transport of GLUT4 protein. IRAP-mOrange-L6 cells were inoculated on sterile coverslips overnight to make the cells completely adherent and then replaced with serum-free α-MEM basic culture media for 2 h to starve cells. Subsequently, we treated the cells with a specific concentration of samples. The images of treated cells were taken by a laser scanning confocal microscope LSM 700 (Carl Zeiss, Jena, Germany) to track the dynamic changes of IRAP-mOrange fluorescence.

### Western Blot Analysis

L6 cells were inoculated into a 60-mm dish at a density of 5 × 105 cells and cultured for 1 week. When the cells were coaxed to differentiating into myotubes in α-MEM containing 2% FBS, they were considered suitable for next experiments. Compound C (Calbiochem, San Diego, CA, United States), wortmannin (Selleckchem, Houston, TX, United States), or Go6983 (EMD Millipore, Billerica, MA, United States) was used to pretreat cells for 30 min before treatment with the indicated concentration of compound 5. After 12 h of incubation in a constant temperature incubator, the remaining medium was gently washed off with PBS and the cells were collected in each dish. Then the RIPA protein extraction kit was applied to crack the cells on ice. The parameters of the high-speed centrifuge were set to centrifuge at 15,000 g for 15 min and then the supernatant was collected. The protein concentration of supernatant was quantitated by a bicinchoninic acid (BCA) protein assay kit (Bio-Rad Laboratories, Munich, Germany). After obtaining the concentration and volume of the protein sample, samples were mixed with an appropriate amount of SDS-PAGE protein loading buffer (5×) and denatured in boiling water at 100 °C for 10 min. An equivalent amount of samples (30 μg) was loaded on the 10% sodium dodecyl sulfate (SDS)–polyacrylamide gel electrophoresis, and an electrophoretic separation was performed. Then the protein was electrotransferred to the polyvinylidene difluoride membrane (Pall Corporation, Washington, United States) activated by methanol. The membrane was blocked with 5% skimmed milk for two hours, followed by p-AMPK antibody, AMPK antibody, GLUT4 antibody, and β-actin (Cell Signaling Technology, Danvers, MA, United States) addition and overnight incubation at 4 °C. Subsequently, the membranes were incubated with secondary antibodies (Abcam, Cambridge, MA, United States) at appropriate dilution for 2 h. Finally, the protein band attached to a membrane was imaged with an enhanced chemiluminescence (ECL) kit and ChemiDoc XRS (Bio-Rad, California, United States).

## RESULTS and DISCUSSION

### Chemistry

**FIGURE 1:**
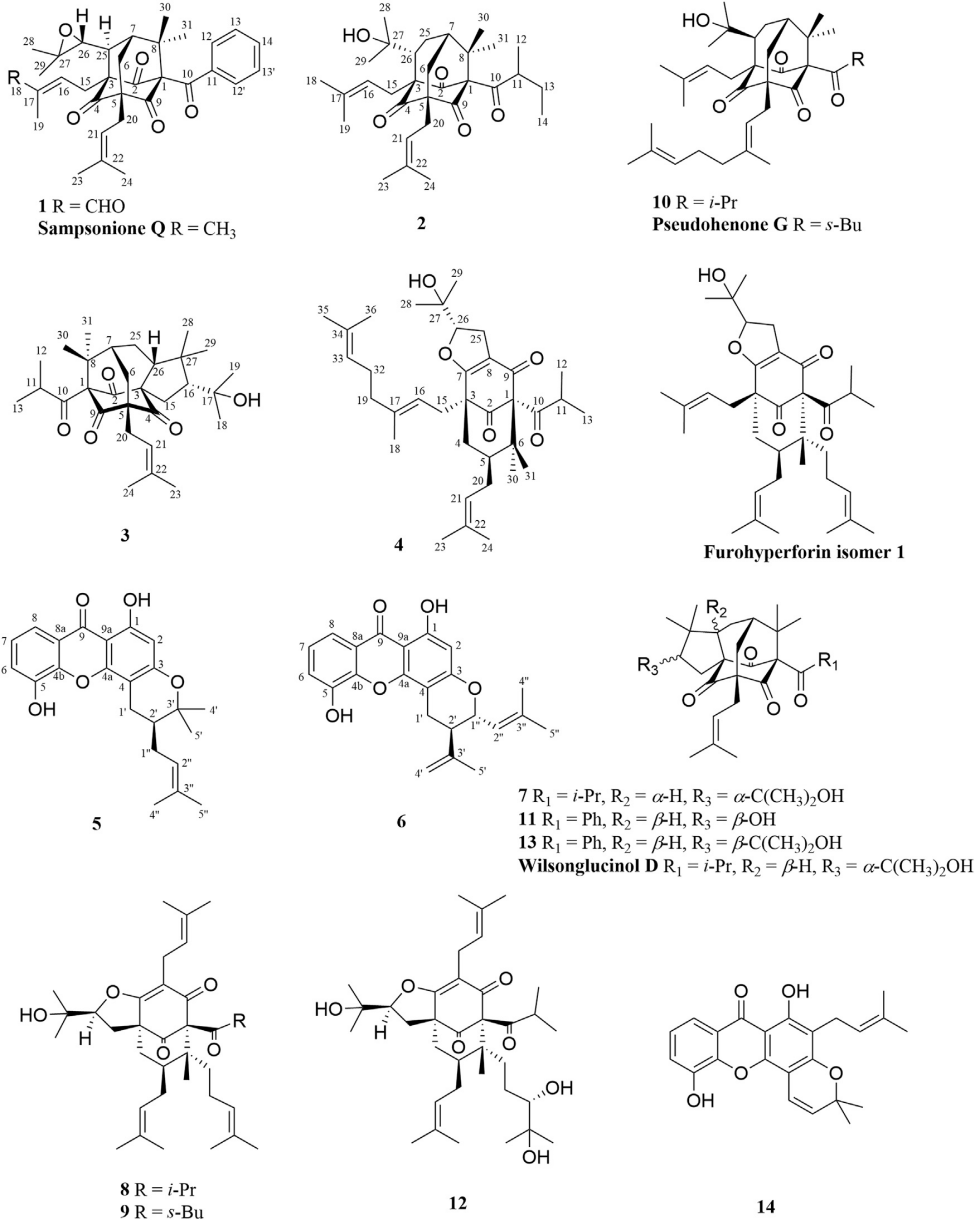
Chemical structures of compounds **1-6**.

**FIGURE 2:**
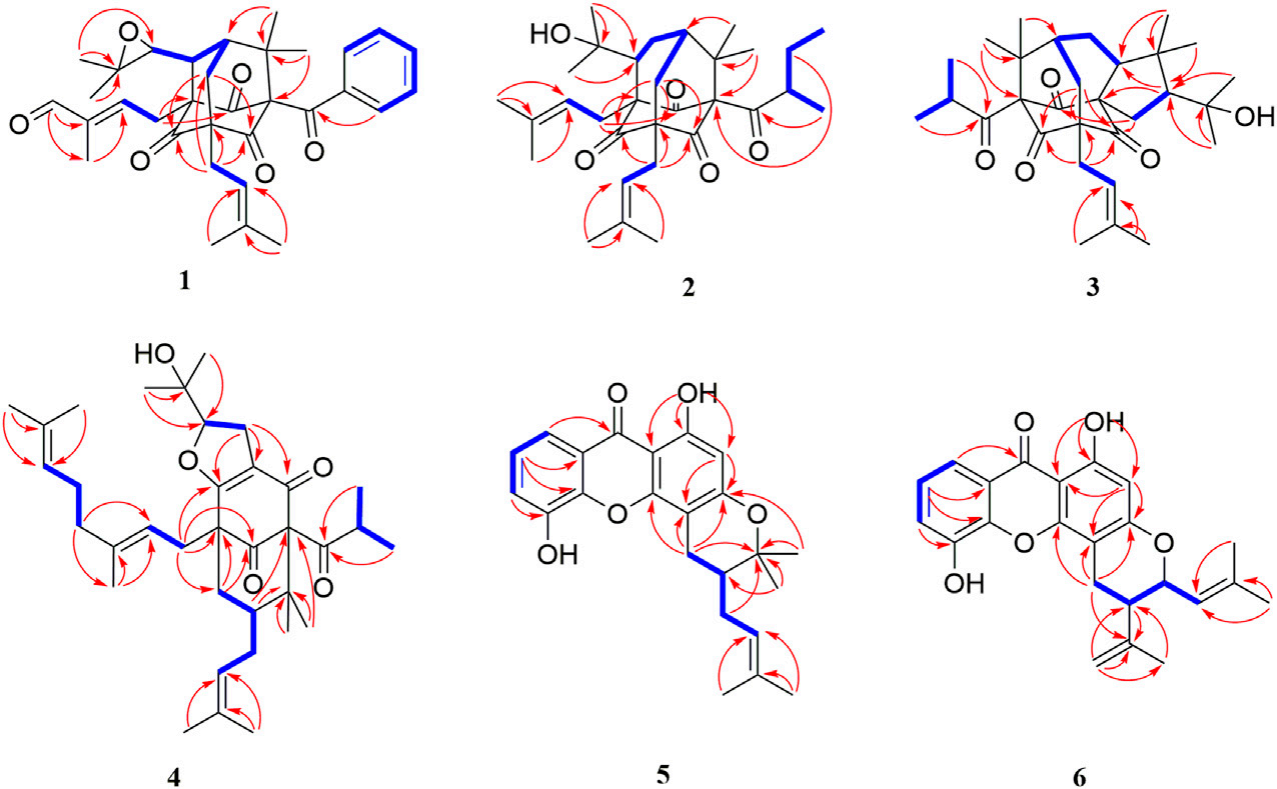
^1^H–^1^H COSY and key HMBC correlations of compounds **1-6**.

**FIGURE 3:**
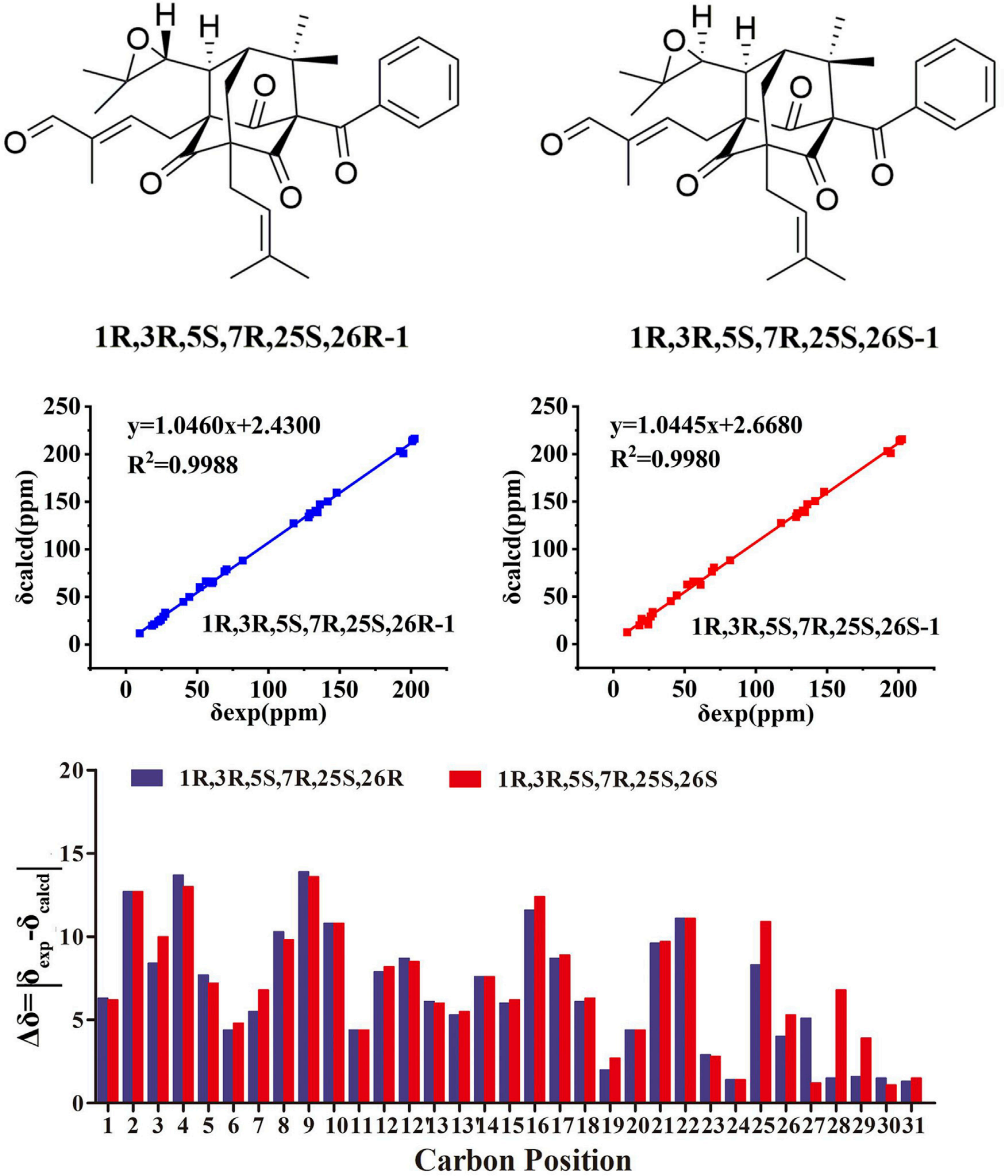
NMR calculations of compound **1**.

The chemical structures of six new compounds (**1–6**) are shown in Figure 1. Compound 1 was obtained as colorless oil. The molecular formula of hyperwilone A 1) was deduced to be C_33_H_38_O_6_ by the [M + H]^+^ ion at m/z 531.2742 in HRESIMS (calcd. 531.2747). The ^1^H NMR data (Table 1) exhibited the signals of an aldehyde proton (δ_H_ 9.30, 1H, s), five phenyl protons (δ_H_ 7.12, 2H; 7.27, 2H; 7.44, 1H), two olefinic protons (δ_H_ 6.29 and 5.18), and seven methyl groups (δ_H_ 1.28–1.83, s). The ^13^C NMR and DEPT data (Table 2) disclosed that 1 possessed 33 carbons involving eight quaternary carbon, four carbonyl, one aldehyde, and ten methine, three methylene, and seven methyl groups. Detailed analysis of these data suggested that the three non-conjugated carbonyls at δ_C_ 200.8 (C-2), 202.6 (C-4), and 201.3 (C-9); four quaternary carbons at δ_C_ 81.9 (C-1), 70.6 (C-3), 69.2 (C-5), and 56.1 (C-8); two methines at δ_C_ 51.8 (C-25) and 44.5 (C-7); and one methylene at δ_C_ 40.3 (C-6) were present for an adamantane-type PPAP (Liao et al., 2015). In addition, the following spectral characteristics were consistent with those of sampsonione Q (Xiao et al., 2007): δ_H_ 2.70 (1H, m, H-25), 2.66 (1H, m, H-26), 1.39, and 1.28 (each 3H, s, H-29, 28), δ_C_ 51.8, 60.5, 61.2, 19.8, and 24.5. The ^13^C-NMR data of 1 were alike to those of sampsonione Q, except that there were eight methyl groups in sampsonione Q, while 1 contained one more aldehyde group and seven methyl groups. In the HMBC spectrum, the correlation of H-18 (δ_H_ 9.30) to C-16, 17, and 19; the correlation of H-16 (δ_H_ 6.29) to C-3, 15, 18, and 19; and the correlation of H−19 (δ_H_ 1.83) to C-16, 17, and 18 indicated that the methyl group at C-18 was oxidized to an aldehyde group (Figure 2). In the ROESY spectrum, the correlation of H−6/Me−30, Me−31/H−25 indicated that H-25 was *α*-orientation. The relative configuration of C-26 was investigated by the TDDFT to calculate the ^13^C NMR data for (1R,3R,5S,7R,25S,26R)-1 and (1R,3R,5S,7R,25S,26S)-1. As shown in Figure 3, the ^13^C NMR chemical shifts of isomers were calculated at the mPW1PW91/6-311G (d,p) level. The calculation result of (1R, 3R, 5S, 7R, 25S, 26R)-1 (R^2^ = 0.9988) matched the experimental data better than (1R, 3R, 5S, 7R, 25S, 26S)-1 (R^2^ = 0.9980) (Li et al., 2020), which indicated that H-26 was *β*-orientation. The absolute configuration of C-1 of 1 was defined as R due to CD spectra of compound 1 showed negative CEs at 333 nm (Zhu et al., 2014). Thus, the absolute configuration of 1 was appointed as (1R, 3R, 5S, 7R, 25S, and 26R). The structure of hyperwilone A 1) was assigned, as shown in Figure 1.

**FIGURE 4:**
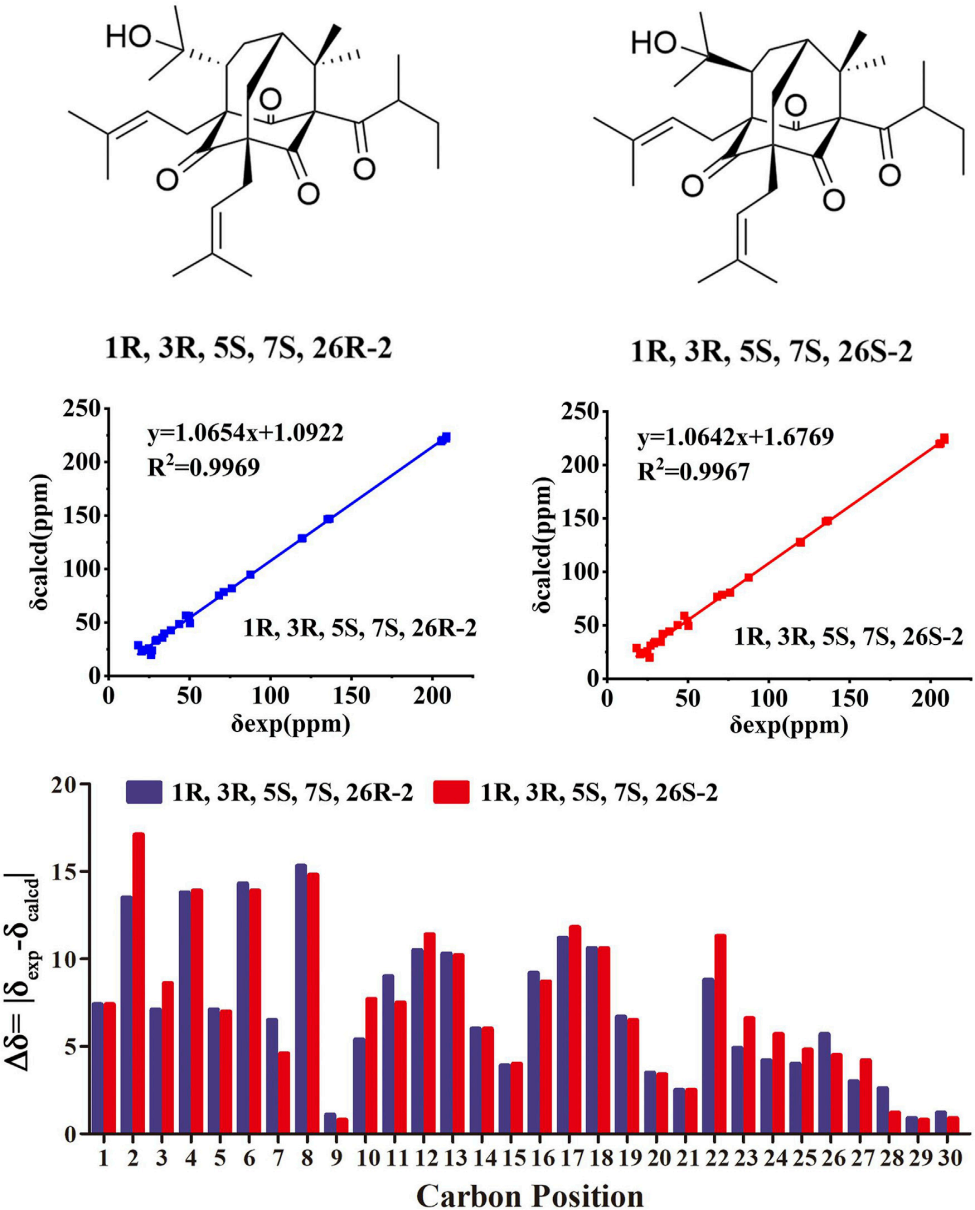
NMR calculations of compound **2**.

Compound **2** was obtained as colorless oil. The molecular formula of hyperwilone B 2) was evidenced to be C_31_H_46_O_5_ by the [M + H]^+^ ion at m/z 499.3416 in HRESIMS (calcd. 499.3423). The ^1^H NMR data (Table 1) demonstrated the signals of two olefinic protons (δ_H_ 5.19 and 4.51), a sec-butyl group (δ_H_ 1.01, 3H; 0.82, 3H; 2.00, 1H and 1.31, 1H; 1.70, 1H), and eight methyls (δ_H_ 1.22–1.72, s). The ^13^C NMR and DEPT data (Table 2) indicated 31 carbon resonances assigned to seven quaternary carbons, four carbonyls, five methines, five methylenes, and ten methyls. An in-depth analysis of these data showed that three non-conjugated carbonyls at δ_C_ 206.3 (C-2), 208.5 (C-4), and 205.5 (C-9); five quaternary carbons at δ_C_ 87.6 (C-1), 71.1 (C-3), 68.1 (C-5), and 47.7 (C-8); two methines at δ_C_ 43.6 (C-7) and 49.7 (C-26); and two methylenes at δ_C_ 38.5 (C-6) and 29.7 (C-25) were likely a homo-adamantane-type PPAP (Yang et al., 2017b). Comparison of its ^1^H and ^13^C NMR data with those of pseudohenone G, a known homo-adamantane-type PPAP from *H*. *pseudohenryi* (Yang et al., 2017b), indicated that they possessed the same carbon skeleton, except that there were an isoprenyl group and a geranyl group in pseudohenone G, while 2 contained two isoprenyl groups. In the HMBC spectrum, the correlation of H-20 (δ_H_ 2.54), H-23 (δ_H_ 1.69), and H-24 (δ_H_ 1.71) to C-5 indicated that an isoprenyl group, instead of a geranyl group, was linked to C-5 (Figure 2). The relative configuration was investigated by the TDDFT to calculate the ^13^C NMR data for (1R, 3R, 5S, 7S, 26S)-2 and (1R, 3R, 5S, 7S, 26R)-2. As shown in Figure 4, the ^13^C NMR chemical shifts of isomers were calculated at the mPW1PW91/6-311G(d,p) level. The calculated results for (1R, 3R, 5S, 7S, 26R)-2 (R^2^ = 0.9969) matched the experimental data better than (1R, 3R, 5S, 7S, 26S)-2 (R^2^ = 0.9967) (Li et al., 2020), which indicated that H-26 was *β*-orientation. Therefore, the structure of hyperwilone B 2) was assigned as shown in Figure 1.

Compound **3** was obtained as colorless oil. The molecular formula of hyperwilone C 3) was evidenced to be C_30_H_45_O_5_ by the [M + H]^+^ ion at m/z 485.3256 in HRESIMS (calcd. 485.3267). The ^1^H NMR data (Table 1) exhibited the signals of an olefinic proton (δ_H_ 5.23), an isopropyl group (δ_H_ 1.02, 3H; 0.99, 3H; 1.88, 1H), and eight methyls (δ_H_ 1.04–1.73, s). The ^13^C NMR data and DEPT (Table 2) indicated that 3 possessed 30 carbons assigned to seven quaternary carbon, four carbonyl, five methine, four methylene, and ten methyl groups. Furthermore, comparing hyperwilone C with wilsonglucinol D by their ^1^H, ^13^C NMR, and MS data (Xie et al., 2020b) indicated that their spectroscopic data were almost identical, indicating that they were isomers and they possessed the same carbon skeleton. The correlation of H-6*β*/H-26, H-26/Me-28, and Me-28/H-16 in the ROESY spectrum indicated that H-16 and H-26 were *β*-orientation, which confirmed that the relative configuration of 3 was the same with wilsonglucinol D. Since CD spectra of 3 showed positive CEs, while wilsonglucinol D showed negative CEs around 330 nm, the absolute configuration of C-1 of 3 was thus determined as S (Xie et al., 2020b) and the absolute configuration of 3 was assigned as (1S,3S,5R,7R,16R,26R). Thus, the structure of 3 was determined as the enantiomer of wilsonglucinol D and named hyperwilone C.

**FIGURE 5:**
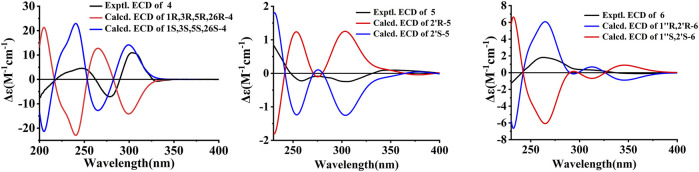
Experimental and the calculated ECD spectra of compounds **4-6**.

Compound **4** was obtained as colorless oil. The molecular formula of hyperwilone D 4) was evidenced to be C_35_H_52_O_5_ by the [M + H]^+^ ion at *m/z* 553.3886 in HRESIMS (calcd. 553.3893). The ^1^H NMR data (Table 1) exhibited the signals of three olefinic protons (δ_H_ 5.06, 5.06, and 4.94), an isopropyl group (δ_H_ 1.13, 3H; 1.03, 3H; 2.14, 1H), and nine methyls (δ_H_ 1.18–1.70, s). The ^13^C NMR and DEPT data (Table 2) indicated that 4 possessed 35 carbons assigned to eight quaternary carbons, three carbonyls, one oxygenated tertiary carbon, and six methines, six methylenes, and eleven methyl groups. Comparing with the data in the literature, some of the signals were assigned to an enolized 1,3-diketo group at δ_C_ 177.7 (C-7), 119.6 (C-8), and 187.6 (C-9), one unconjugated carbonyl carbon at δ_C_ 207.0 (C-2), a methylene at δ_C_ 38.0 (C-4), a methane at δ_C_ 48.0 (C-5), and three quaternary carbons at δ_C_ 81.3 (C-1), 52.7 (C-3), and 47.7 (C-6). Those characteristic NMR data indicated that 4 was a PPAP derivative with a [3.3.1] ring. In addition, the following characteristic spectral features were consistent with those of furohyperforin (Lee et al., 2006): δ_H_ 4.75 (H-26), 2.96 (H-25*α*), 2.86 (H-25*β*), 1.30 (H-28), and 1.19 (H-29), δ_C_ 93.2 (C-26), 71.9 (C-27), 27.6 (C-25), 26.4 (C-28), and 23.6 (C-29). The ^13^C-NMR data (Table 2) of 4 were similar to those of furohyperforin isomer 1 (Lee et al., 2006), except that 4 contained one more geranyl group, instead of two isoprenyl groups in furohyperforin isomer 1. In the ^1^H-^1^H COSY spectrum, the correlation of H-20 (δ_H_ 1.82) to H-5 (δ_H_ 1.42) indicated that isoprenyl group was linked to C-5 (Figure 2). In the HMBC spectrum, the correlation of H-15 (δ_H_ 2.50) to C-2, 3, 4, 7, and the correlation of H-16 (δ_H_ 5.06) to C-3 indicated that geranyl group was linked to C-3 (Figure 2). The correlation of H-11 (δ_H_ 2.14), H-12 (δ_H_ 1.03), and H-13 (δ_H_ 1.13) to C-10 indicated that the isopropyl group was linked to C-10 (Figure 2). The correlation of H-30 (δ_H_ 1.21) to C-1, 6 and the correlation of H-31 (δ_H_ 1.33) to C-1, 6 indicated that two methyls were linked to C-6, instead of one isoprenyl group, and one methyl were linked to C-6 (Figure 2). H-5 was in the *α*-orientation, based on the C-5 chemical shift (δ_C_ 48.0) and the chemical shift difference between H-4*β* and H-4*α* (Δδ ca.0.0) (Chen et al., 2010). In addition, the relative configuration of C-2 and C-6 of compound 4 were comfirmed to be the same as those of furohyperforin by the correlations of H-5/Me-12 and H-15 in the ROESY spectrum of 4. The ROESY of H-15/Me-28 indicated that H-26 was *β*-orientation. The absolute configuration of 4 was defined as (1S,3S,5S,26S)-4 by comparison of the calculated and experimental ECD spectra (Figure 5). Therefore, the structure of hyperwilone D 4**)** was determined as shown in Figure 1.

Compound **5** was obtained as yellow powder. The molecular formula of wilsonxanthone A 5) was evidenced to be C_23_H_24_O_5_ by the [M + H]^+^ ion at *m/z* 381.1696 in HRESIMS (calcd. 381.1702). The ^1^H NMR data (Table 3) exhibited the signals of an ABC spin system assigned to a 1,2,3-trisubstituted benzene ring at δH 7.78 (dd, J = 1.2, 7.9 Hz), 7.33 (dd, J = 1.2; 7.9 Hz), and 7.27 (t, J = 7.9 Hz), an aromatic proton at δ_H_ 6.28, an olefinic proton at δ_H_ 5.21 (t, *J* = 7.5 Hz), a hydroxyl proton at δ_H_ 12.63, and four methyls (δ_H_ 1.27, 1.48, 1.61, and 1.75, s). The ^13^C NMR and DEPT data (Table 3) indicated that compound 5 possessed 23 carbons comprising ten quaternary carbons, one carbonyl, six methines, two methylenes, and four methyl groups. Comparing with the data in the literature (Mountessou et al., 2018) the 2,2-dimethyldihydropyran moiety was inferred from the signals at δ_H_ 2.98 (dd, *J* = 16.3, 5.1 Hz) and 2.44 (dd, *J* = 16.3, 9.3 Hz), δ_H_ 1.86 (1H, m) and δ_H_ 1.48 and 1.27 (3H each, s) which was also confirmed by the signals at δ_C_ 79.9, 40.8, 27.6, 21.9, and 21.4 in the ^13^C NMR spectrum. The 2,2-dimethyldihydropyran group was attached at C-3 and C-4 of the xanthone skeleton of compound 5 as illustrated by HMBC correlations observed between the methylene protons at H-1' (δ_H_ 2.98 and 2.44) with the carbon signals at C-3 (δ_C_ 161.5), C-4a (153.9), C-4 (100.1), C-3' (79.9), and C-2' (40.8). On the other hand, the HMBC correlations between hydroxyl proton with the carbon signals at C-1 (δ_C_ 161.3) and C-9a (δ_C_ 103.6) confirmed that hydroxyl group was attached at C-1 of the xanthone skeleton. An isoprenyl group was determined by the presence of signals at δ_H_ 5.21 (t, *J* = 7.5 Hz), δ_H_ 2.33 and 1.90 (1H each, m), δ_H_ 1.75 and 1.61 (3H each, s) which were further confirmed by the resonances at δ_C_ 133.9, 122.1, 29.3, 26.0, and 18.1 in the ^13^C NMR spectrum, respectively. Thus, HMBC correlations between the methylene protons at H-1''*α* (δ_H_ 2.33) and the carbon signals at C-3' (δ_C_ 79.9), C-2' (δ_C_ 40.8), and C-4' (δ_C_ 21.4); H-1'' (δ_H_ 1.90) and the carbon signals at C-2' (δ_C_ 40.8) clearly identified the point of attachment at C-2′ of the 2,2-dimethyldihydropyran ring (Figure 2). The absolute configuration of the C-2′ was determined by a comparison of the experimental and calculated ECD data (Figure 5). Thus, the (2′S) absolute configuration of 5 was defined. Thus, wilsonxanthone A 5) with [α] 20D −10.1 was assigned as the structure in Figure 1.

Compound **6** was obtained as yellow powder. The molecular formula of wilsonxanthone B 6) was evidenced to be C_23_H_22_O_5_ by the [M + H]^+^ ion at *m/z* 379.1539 in HRESIMS (calcd. 379.1545). The ^1^H NMR data (Table 3) exhibited the signals of an ABC spin system at δ_H_ 7.80 (dd, *J* = 1.5, 7.9 Hz), 7.33 (dd, *J* = 1.5, 7.9 Hz), and 7.28 (t, *J* = 7.9 Hz) assignable to a 1,2,3-trisubstituted benzene ring, an aromatic proton at δ_H_ 6.35(s), three olefinic protons at δ_H_ 5.29 (d, *J* = 9.1 Hz), 4.95, and 4.89 (1H each, s), a hydroxyl proton at δ_H_ 12.65 and three methyls at δ_H_ 1.83 (d, *J* = 0.9 Hz), 1.79 (d, *J* = 0.9 Hz), and 1.78 (s). The ^13^C NMR and DEPT data (Table 3) indicated that compound 6 possessed 23 carbons comprising ten quaternary carbons, one carbonyl and seven methines, two methylenes, and three methyl groups. According to these data, the structure of 6 was similar with 5, except that the branched chain attached to the dihydropyran ring was different. In the HMBC spectrum, the correlation of H-4' (δ_H_ 4.95 and 4.89) to C-3' (δ_C_ 144.6) and C-5' (δ_C_ 20.6), H-5' (δ_H_ 1.78) to C-3′ and C-4' (δ_C_ 113.8) indicated that there was an isopropenyl group. The correlation of H-2' (δ_H_ 2.54) to C-3′, 4′ and 5′, H-4′ to C-2' (δ_C_ 44.2) and H-5′ to C-2′ indicated that the isopropenyl group was link to the dihydropyran ring at C-2'. The correlation of H-2'' (δ_H_ 5.29) to C-4'' (δ_C_ 26.1) and C-5'' (δ_C_ 18.8), H-4'' (δ_H_ 1.83) to C-2'' (δ_C_ 122.3) and C-3'' (δ_C_ 140.3), and H-5'' (δ_H_ 1.79) to C-2'', 3'' and 4'' in the HMBC spectrum indicated that there was a 2-methyl-1-propenyl group. The correlation between H-2'' and H-1'' (δ_H_ 4.74) in the ^1^H-^1^H COSY spectrum and the correlation of H-1'' to C-3'', H-2′ to C-2", and H-2" to C-2′ in the HMBC indicated that the 2-methyl-1-propenyl group was link to the dihydropyran ring at C-1'' (Figure 2). In the ROESY spectrum of 6, the correlations of H-2'/H-2'' and H-1''/Me-5′ indicated that the relative configurations of H-2′ and H-1'' were opposite orientation, which was further confirmed by the coupling constant value of 9.6 Hz between H-2′ and H-1'' in the ^1^H NMR spectrum. The absolute configuration of 6 was determined to be (1''R,2′R) by comparing the experimental and calculated ECD spectra (Figure 5). Thus, wilsonxanthone B (6) with [α] 20D +29.3 was assigned as the structure in Figure 1.

Compounds 7–14 were also obtained from petroleum ether extract of *H wilsonii*. Comparing their NMR spectroscopic data of these compounds with values reported in the literature, these known compounds were identified as wilsonglucinol G 7) (Xie et al., 2020b), furohyperforin 8) (Lee et al., 2006), furoadhyperforin 9) (Lee et al., 2006), pseudohenone F (10) (Yang et al., 2017b), pseudohenone E (11) (Yang et al., 2017b), attenuatumione G 12) (Zhou et al., 2016), sampsonione G 13) (Christian et al., 2001), and ananixanthone 14) (Zamakshshari et al., 2019).

### GLUT4 Translocation Effects of Compounds 1–14

**FIGURE 6:**
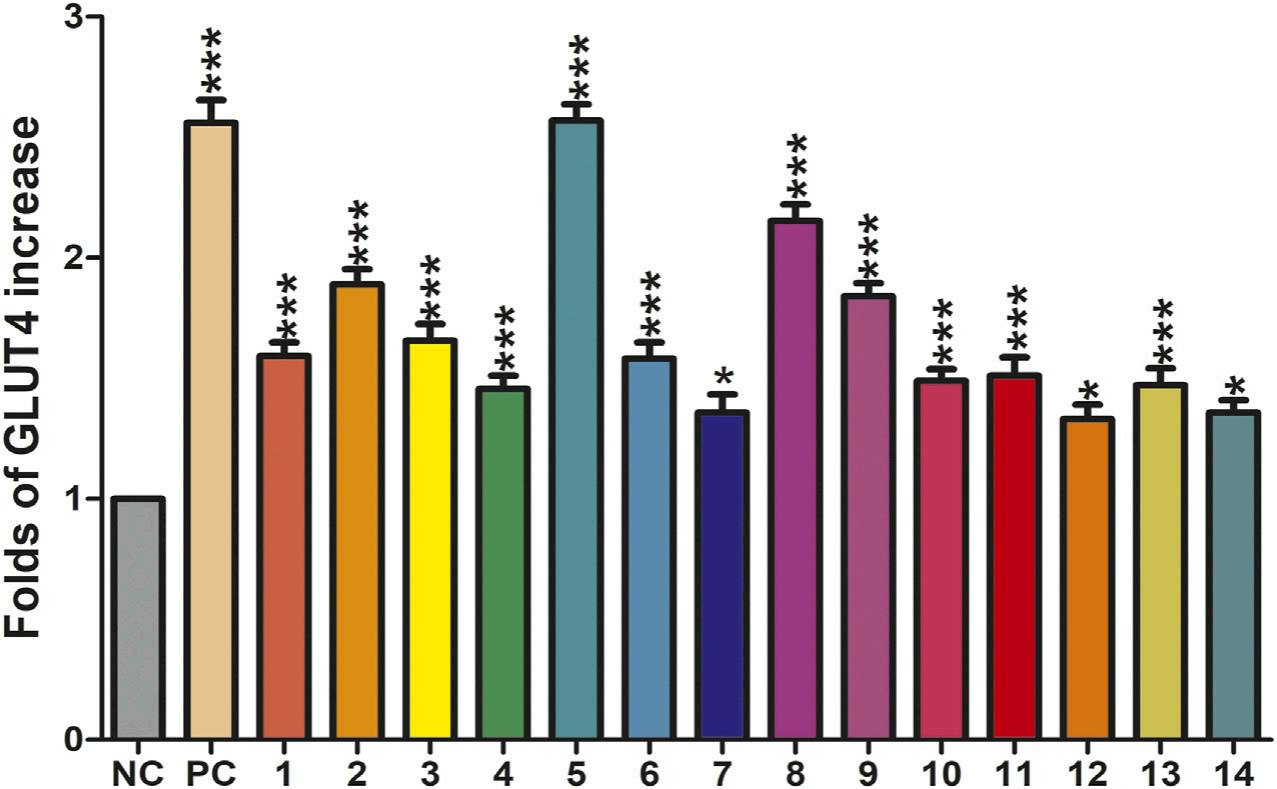
Effects of fourteen compounds on GLUT4 translocation in L6 cells (*p < 0.05, compared with non-treated groups (NC); ***p < 0.001, compared with the NC group).

**FIGURE 7:**
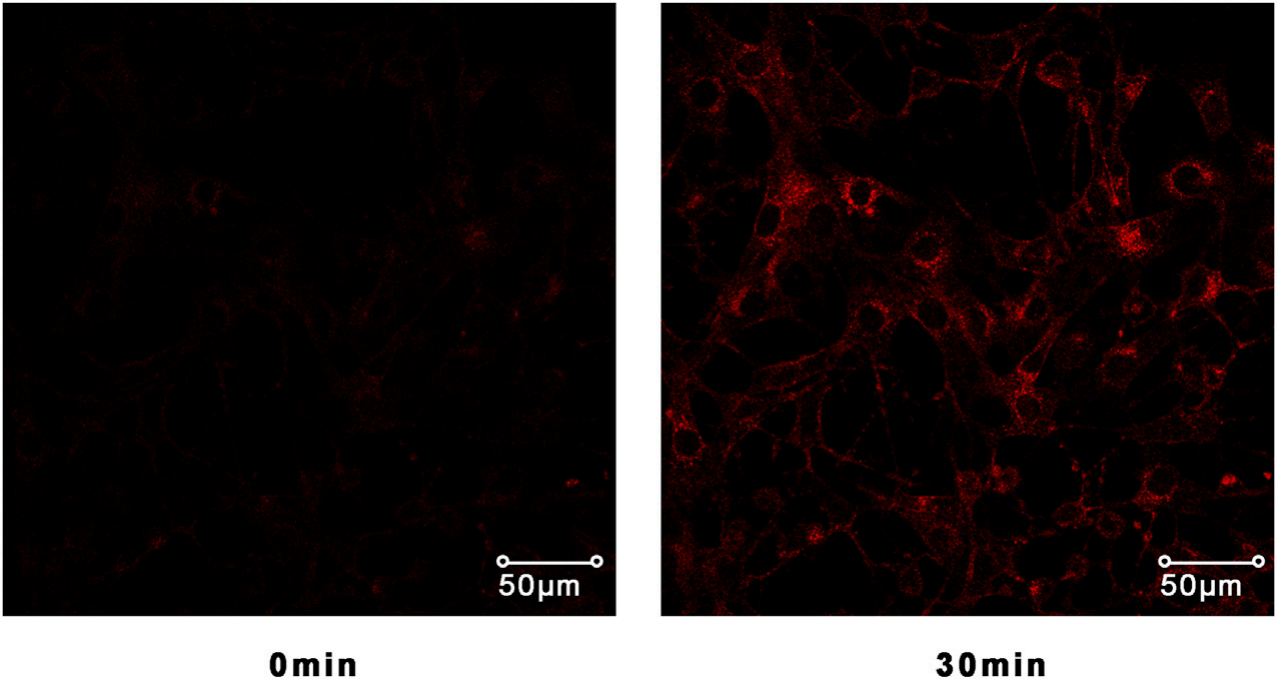
Confocal microscope was used to track IRAP fluorescence changes in L6 cells. L6 cells exposed to the compound **5** for 30 min could significantly induce IRAP fluorescence enhancement.

The translocation change of GLUT4 caused by compounds 1–14 could be reflected by the IRAP fluorescence intensity on the L6 cell membrane. After incubating with samples, the IRAP-mOrange fluorescence intensity at the plasma membrane shows varying degrees of change (Figure 6). Insulin (100 nM) was used as the positive control (PC). Wilsonxanthone A 5) and furohyperforin 8) exerted strong GLUT4 translocation effects, which were enhanced by 1.57 and 1.15 folds, respectively. Hyperwilone A-C (1–3), wilsonxanthone B (6), furoadhyperforin (9), and pseudohenone E (11) show weak to moderate activity with 0.51- to 0.89-fold enhancement, respectively. In Figure 7, compound 5 notably stimulated IRAP fluorescence intensity enhancement after 30 min of exposure to myotubes. This result showed that compound 5 significantly affected GLUT4 translocation in L6 cells. In conclusion, wilsonxanthone A (5), a new xanthone isolated from the aerial parts of *Hypericum wilsonii*, may possess antidiabetic activity.

### Compound 5 Enhanced GLUT4 Translocation and Expression *via* AMPK Pathway

**FIGURE 8:**
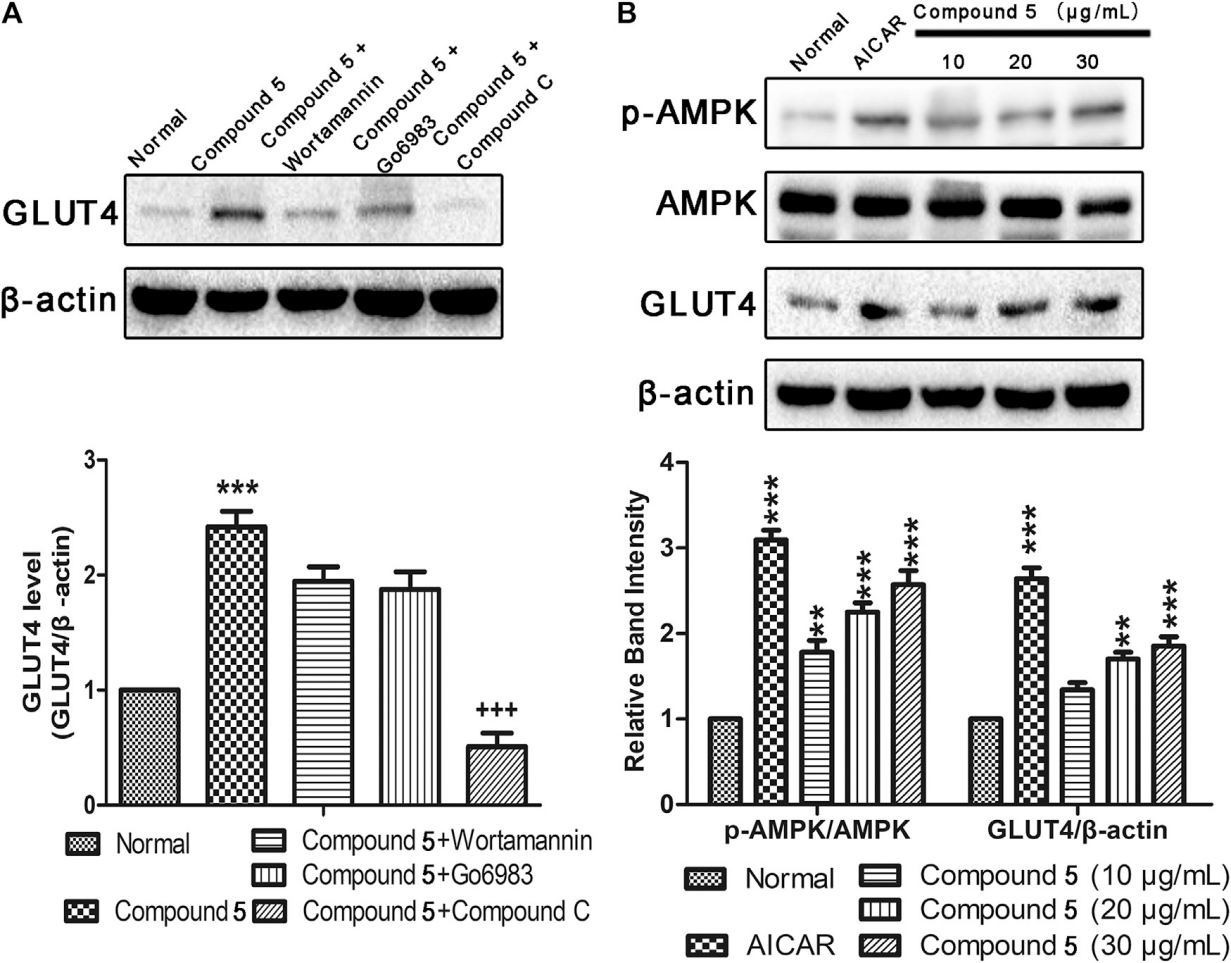
Effects of compound **5** *in vitro*. **(A)** Compound C completely inhibited the GLUT4 expression induced by compound 5 (30 μg/ml), while Wortmannin and Go6983 showed no obvious effects. ***p < 0.001, compared with normal, ^+++^ *p* < 0.001 compared with compound 5. **(B)** Compound 5 at 30 μg/ml notably enhanced AMPK phosphorylation and GLUT4 expressions, respectively. Data are shown as mean ± SEM. *p < 0.05, **p < 0.01, ***p < 0.001 compared with NC group.

Previous research has indicated that AMPK, PI3K/Akt, and PKC pathways participated in regulation of GLUT4 transport and expression (Elmendorf, 2002; Thong et al., 2005). Therefore, the inhibitors of corresponding pathways were used to pretreat L6 cells to further explore the mechanism by which compound 5 stimulates GLUT4 expression. Western blotting results show that the effects of GLUT4 increase trigged by compound 5 were totally repressed when compound 5 accompanied by compound C was added (Figure 8A). In addition, the level of p-AMPK/AMPK and GLUT4 expression was obviously increased following treatment with different concentrations (10, 20, and 30 μg/ml) of compound 5. When the dose of compound 5 was 30 μg/ml, it exerted the strongest stimulatory effect on the translocation of GLUT4 (Figure 8B). The aforementioned results indicated that compound 5 promotes GLUT4 translocation and expression activation through AMPK pathway in a certain dose-dependent manner.

## Conclusion

In conclusion, fourteen compounds including polyprenylated acylphloroglucinol derivatives and xanthones were isolated from *H. wilsonii*, in which six compounds were new compounds. Their structures were elucidated on the basis of extensive 1D and 2D NMR spectroscopic data analysis. And the relative configurations and absolute configurations of compounds 1–6 were elucidated based on NMR calculates and comparison of experimental and calculated ECD spectra. Compounds 5 increased GLUT4 translocation and expression via the AMPK pathway. The discovery of new polyprenylated acylphloroglucinol derivatives and xanthones revealed the chemical composition and potentially antidiabetic medicinal value of *H. wilsonii*.

## Data Availability

The original contributions presented in the study are included in the article/Supplementary Material; further inquiries can be directed to the corresponding authors.
