# Supplementary material for: New Polyprenylated Acylphloroglucinol Derivatives and Xanthones From Hypericum wilsonii
Source: Front Chem. 2021 Sep 24;9:717904. doi: 10.3389/fchem.2021.717904 (PMC8497742; doi:10.3389/fchem.2021.717904)
Supplement: Supplementary file 1 [file DataSheet1.doc]

Supplementary Material

S1. ^1^H NMR spectrum of compound **1** (CDCl_3_, 600 MHz)

S2. ^13^C NMR spectrum of compound **1** (CDCl_3_, 150 MHz)

S3. DEPT 135° spectrum of compound **1** (CDCl_3_, 150 MHz)

S4. HSQC spectrum of compound **1**

S5. HMBC spectrum of compound **1**

S6. ^1^H-^1^H COSY spectrum of compound **1**

S7. ROESY spectrum of compound **1**

S8. CD spectrum of **1**

S9. IR spectrum of **1**

S10. UV spectrum of **1**

S11. HRESIMS spectrum of **1**

S12. ^1^H NMR spectrum of compound **2** (CDCl_3_, 600 MHz)

S13. ^13^C NMR spectrum of compound **2** (CDCl_3_, 150 MHz)

S14. DEPT 135° spectrum of compound **2** (CDCl_3_, 150 MHz)

S15. HSQC spectrum of compound **2**

S16. HMBC spectrum of compound **2**

S17. ^1^H-^1^H COSY spectrum of compound **2**

S18. ROESY spectrum of compound **2**

S19. IR spectrum of **2**

S20. UV spectrum of **2**

S21. HRESIMS spectrum of **2**

S22. ^1^H NMR spectrum of compound **3** (CDCl_3_, 600 MHz)

S23. ^13^C NMR spectrum of compound **3** (CDCl_3_, 150 MHz)

S24. DEPT 135° spectrum of compound **3** (CDCl_3_, 150 MHz)

S25. HSQC spectrum of compound **3**

S26. HMBC spectrum of compound **3**

S27. ^1^H-^1^H COSY spectrum of compound

S28. ROESY spectrum of compound **3**

S29. CD spectrum of **3**

S30. IR spectrum of **3**

S31. UV spectrum of **3**

S32. HRESIMS spectrum of **3**

S33. ^1^H NMR spectrum of compound **4** (CDCl_3_, 600 MHz)

S34. ^13^C NMR spectrum of compound **4** (CDCl_3_, 150 MHz)

S35. DEPT 135° spectrum of compound **4** (CDCl_3_, 150 MHz)

S36. HSQC spectrum of compound **4**

S37. HMBC spectrum of compound **4**

S38. ^1^H-^1^H COSY spectrum of compound **4**

S39. ROESY spectrum of compound **4**

S40. CD spectrum of **4**

S41. IR spectrum of **4**

S42. UV spectrum of **4**

S43. HRESIMS spectrum of **4**

S44. ^1^H NMR spectrum of compound **5** (CDCl_3_, 600 MHz)

S45. ^13^C NMR spectrum of compound **5** (CDCl_3_, 150 MHz)

S46. DEPT 135° spectrum of compound **5** (CDCl_3_, 150 MHz)

S47. HSQC spectrum of compound **5**

S48. HMBC spectrum of compound **5**

S49. ^1^H-^1^H COSY spectrum of compound **5**

S50. ROESY spectrum of compound **5**

S51. CD spectrum of **5**

S52. IR spectrum of **5**

S53. UV spectrum of **5**

S54. HRESIMS spectrum of **5**

S55. ^1^H NMR spectrum of compound **6** (CDCl_3_, 600 MHz)

S56. ^13^C NMR spectrum of compound **6** (CDCl_3_, 150 MHz)

S57. DEPT 135° spectrum of compound **6** (CDCl_3_, 150 MHz)

S58. HSQC spectrum of compound **6**

S59. HMBC spectrum of compound **6**

S60. ^1^H-^1^H COSY spectrum of compound **6**

S61. ROESY spectrum of compound **6**

S62. CD spectrum of **6**

S63. IR spectrum of **6**

S64. UV spectrum of **6**

S65. HRESIMS data of **6**

S66. NMR calculation section

S67. ECD calculation section


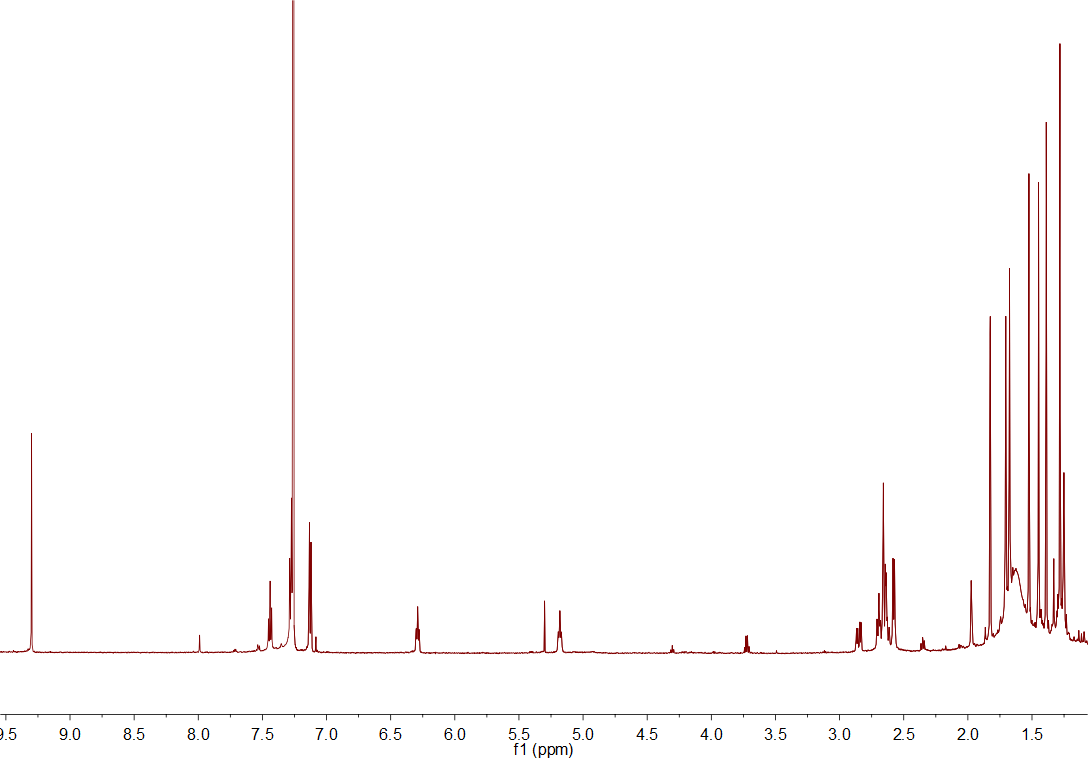


S1. ^1^H NMR spectrum of compound **1** (CDCl_3_, 600 MHz)


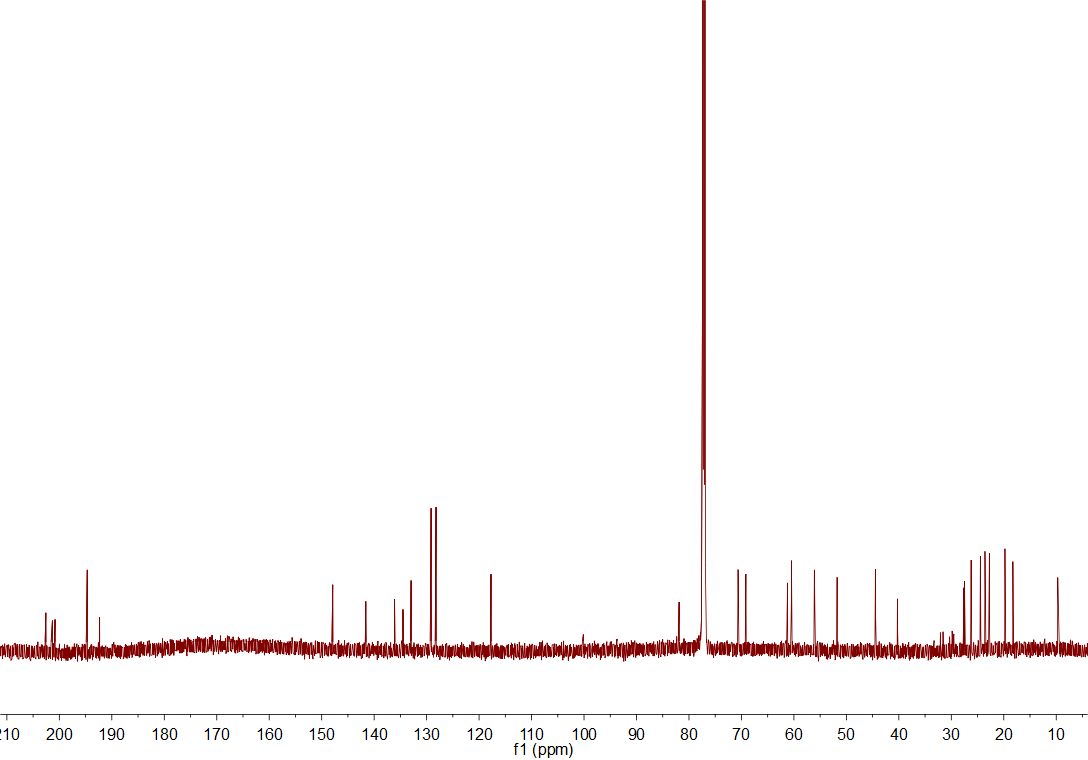


S2. ^13^C NMR spectrum of compound **1** (CDCl_3_, 150 MHz)


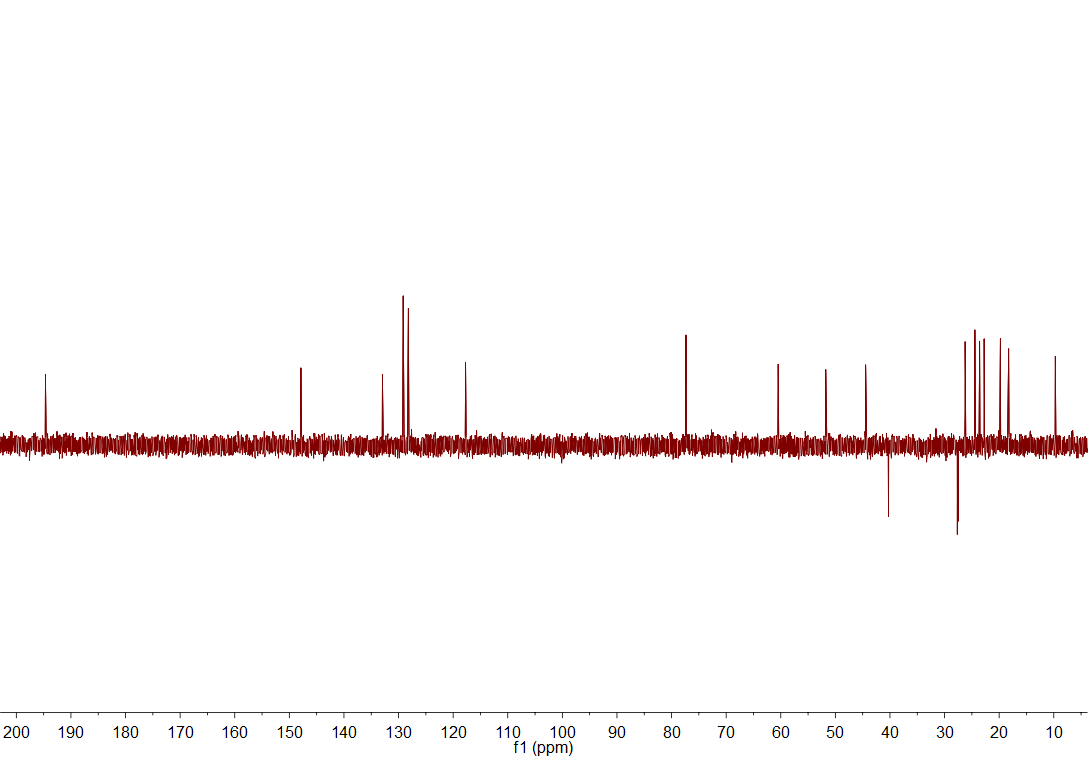


S3. DEPT 135° spectrum of compound **1** (CDCl_3_, 150 MHz)


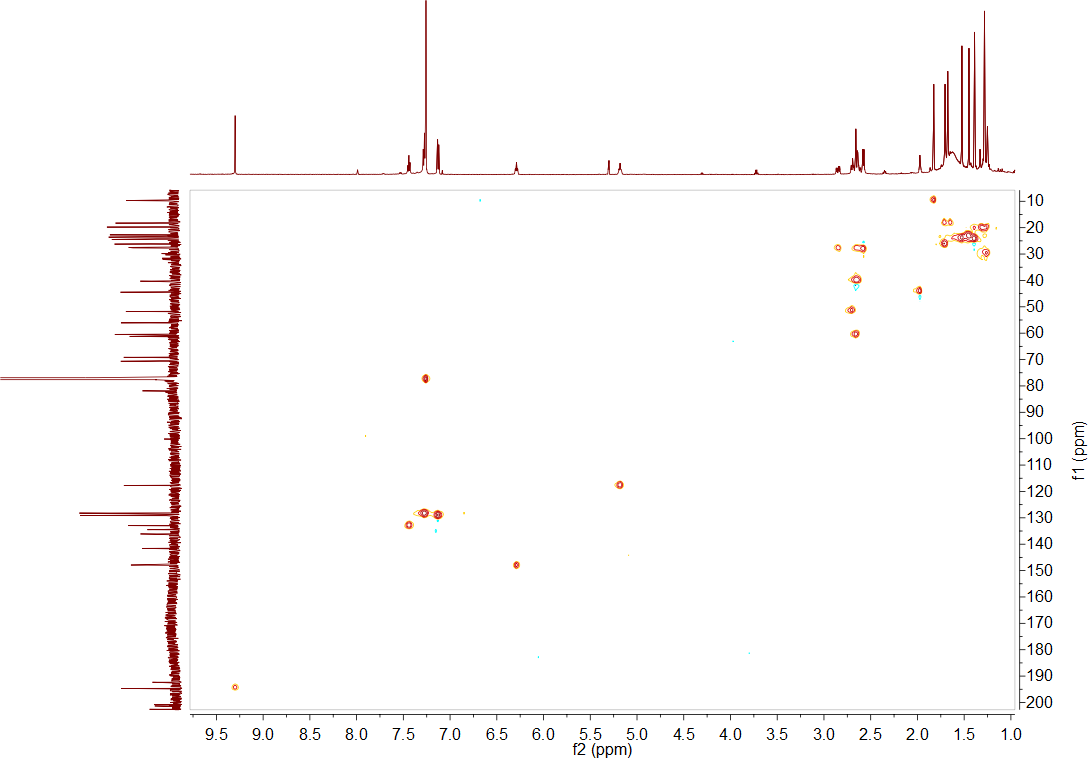


S4. HSQC spectrum of compound **1** (CDCl_3_)


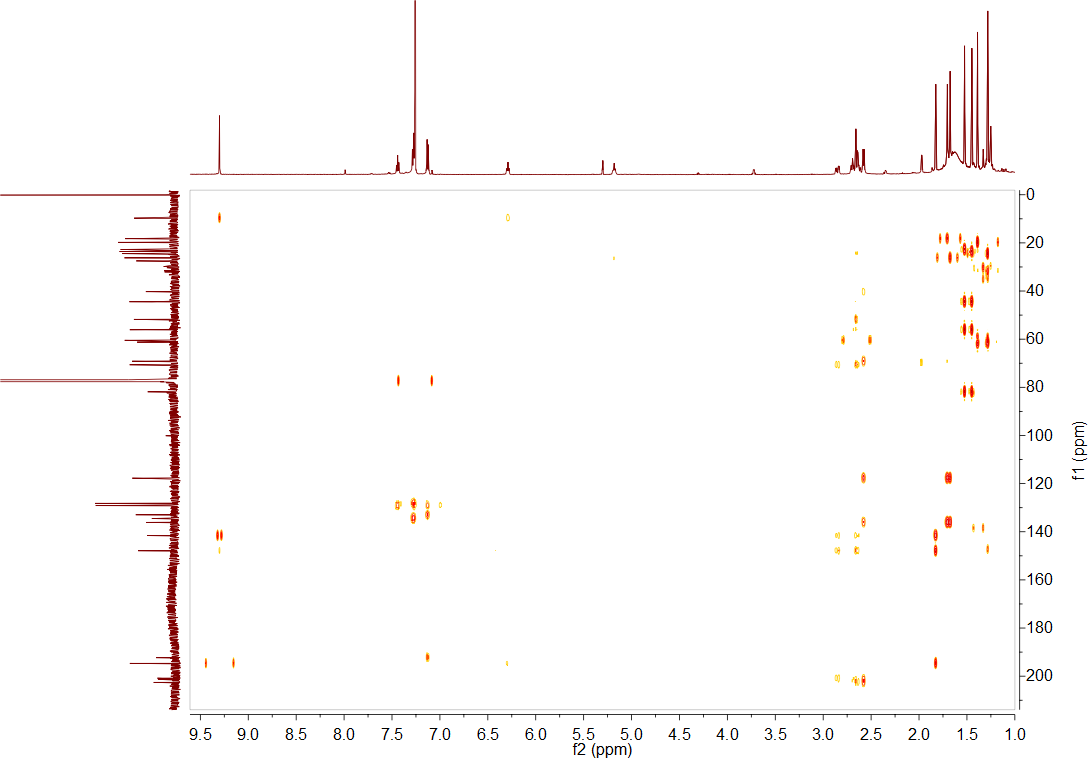


S5. HMBC spectrum of compound **1** (CDCl_3_)


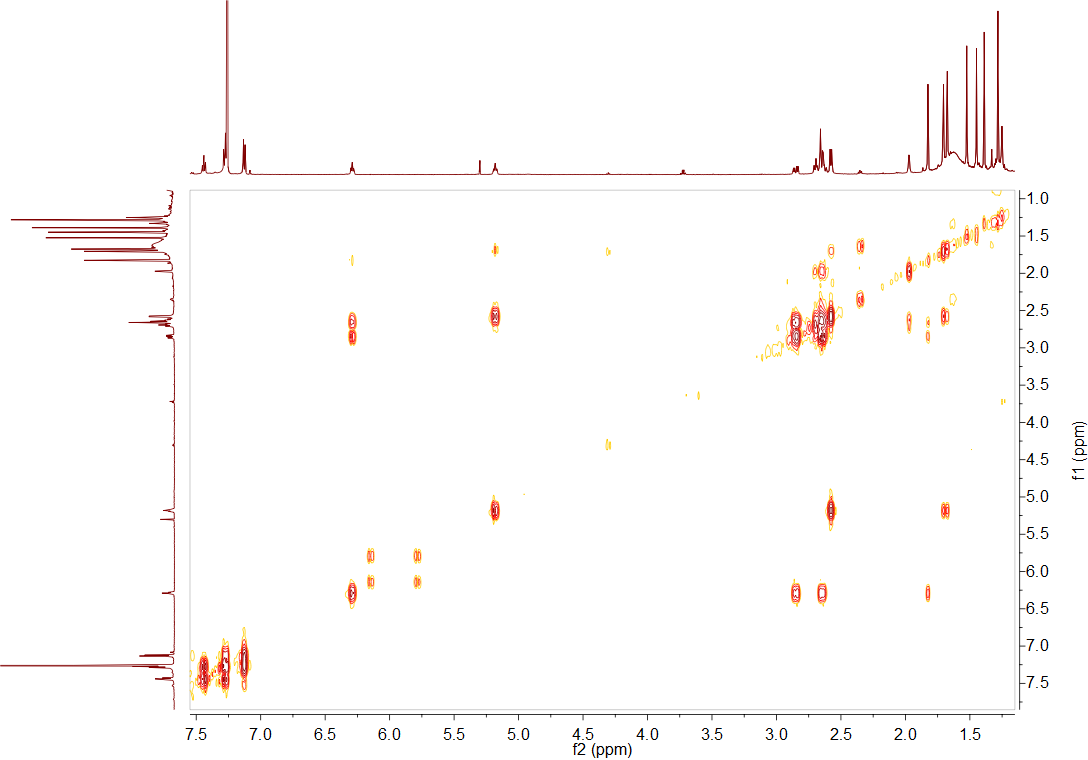


S6. ^1^H-^1^H COSY spectrum of compound **1** (CDCl_3_)


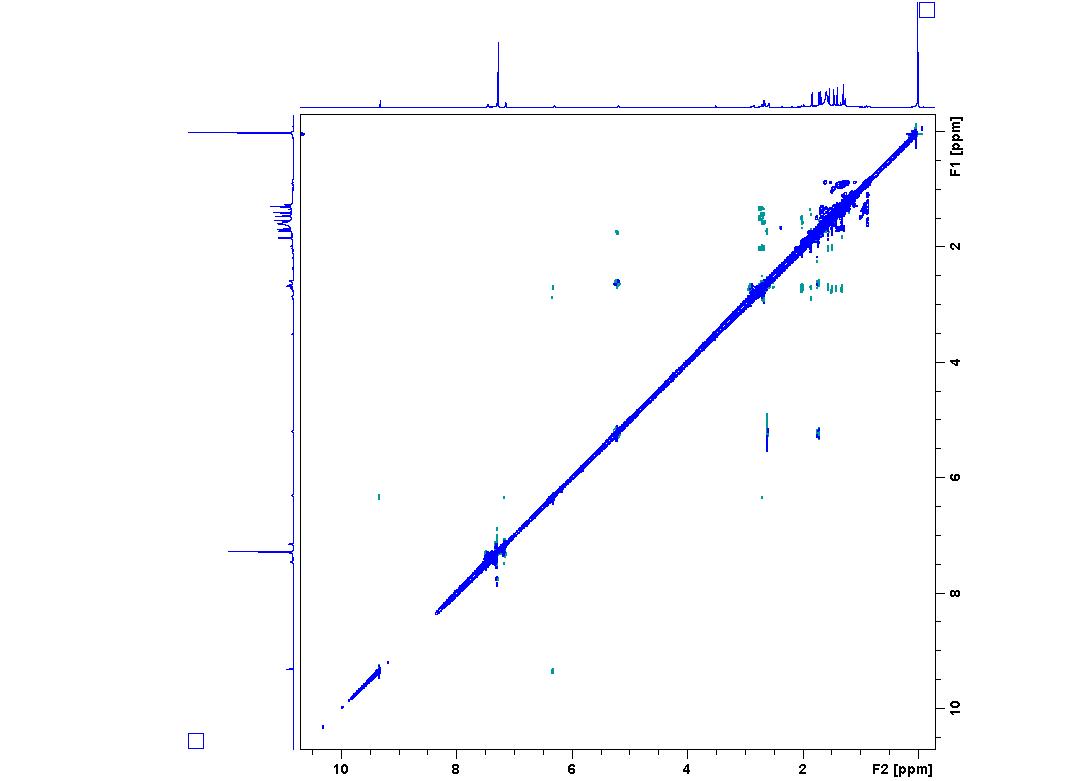


S7. ROESY spectrum of compound **1** (CDCl_3_)


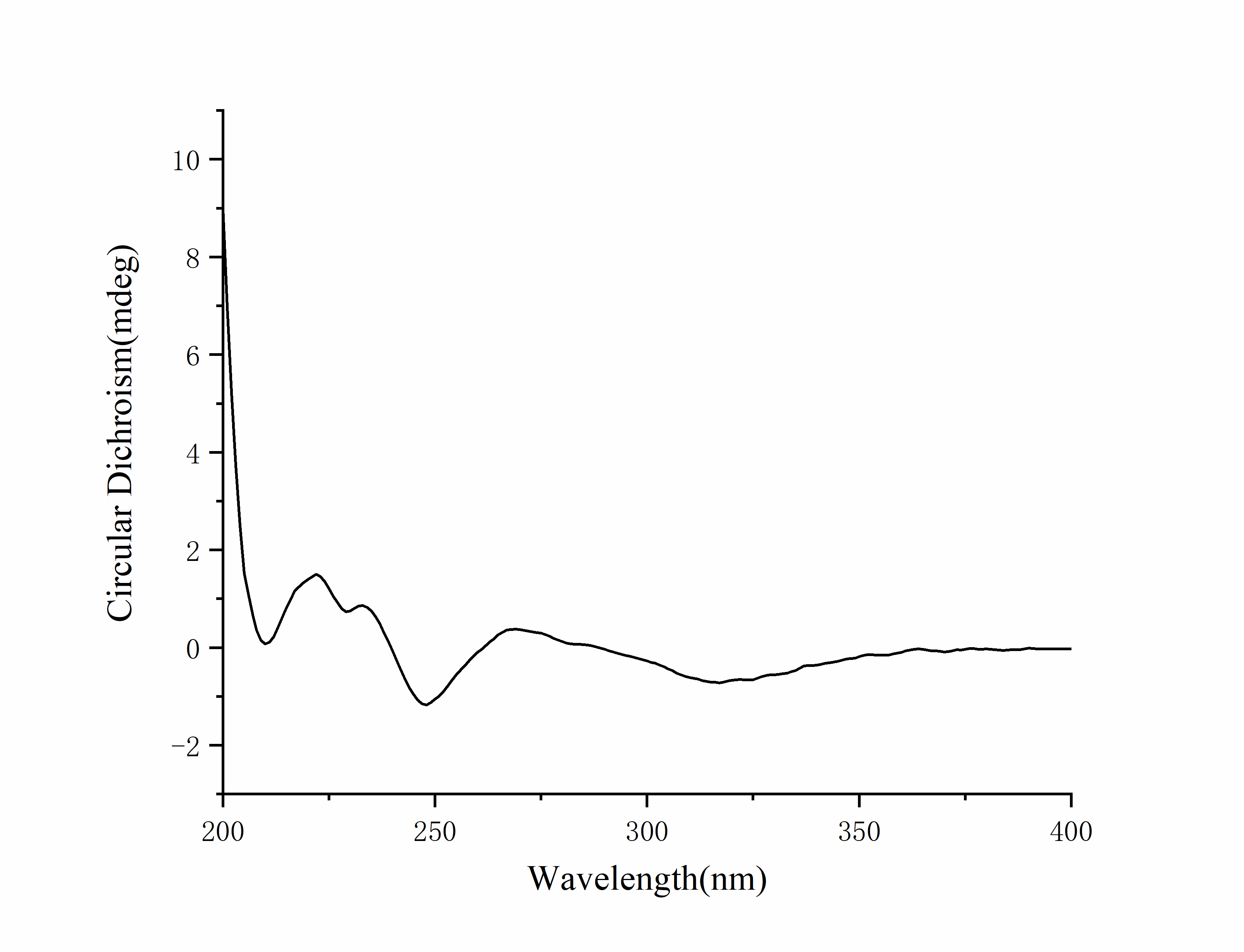


S8. CD spectrum of **1**

**
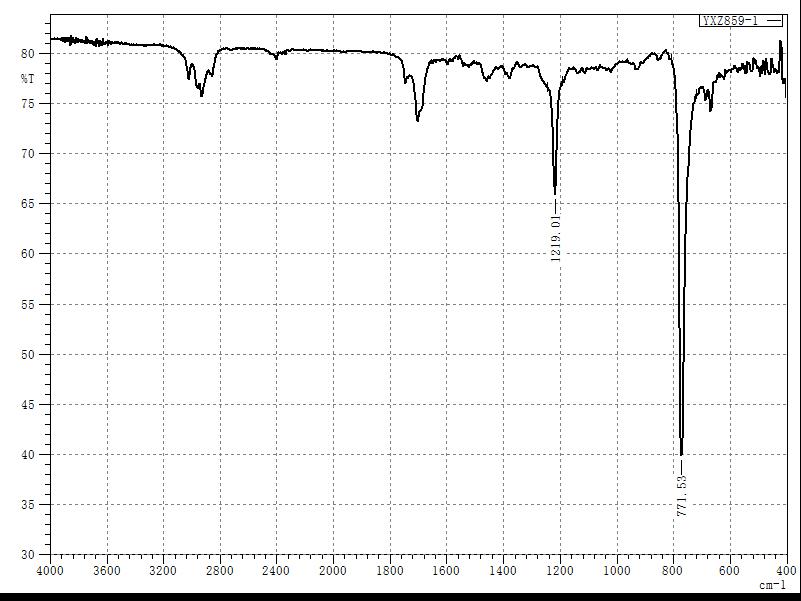
**

S9. IR spectrum of **1**

**
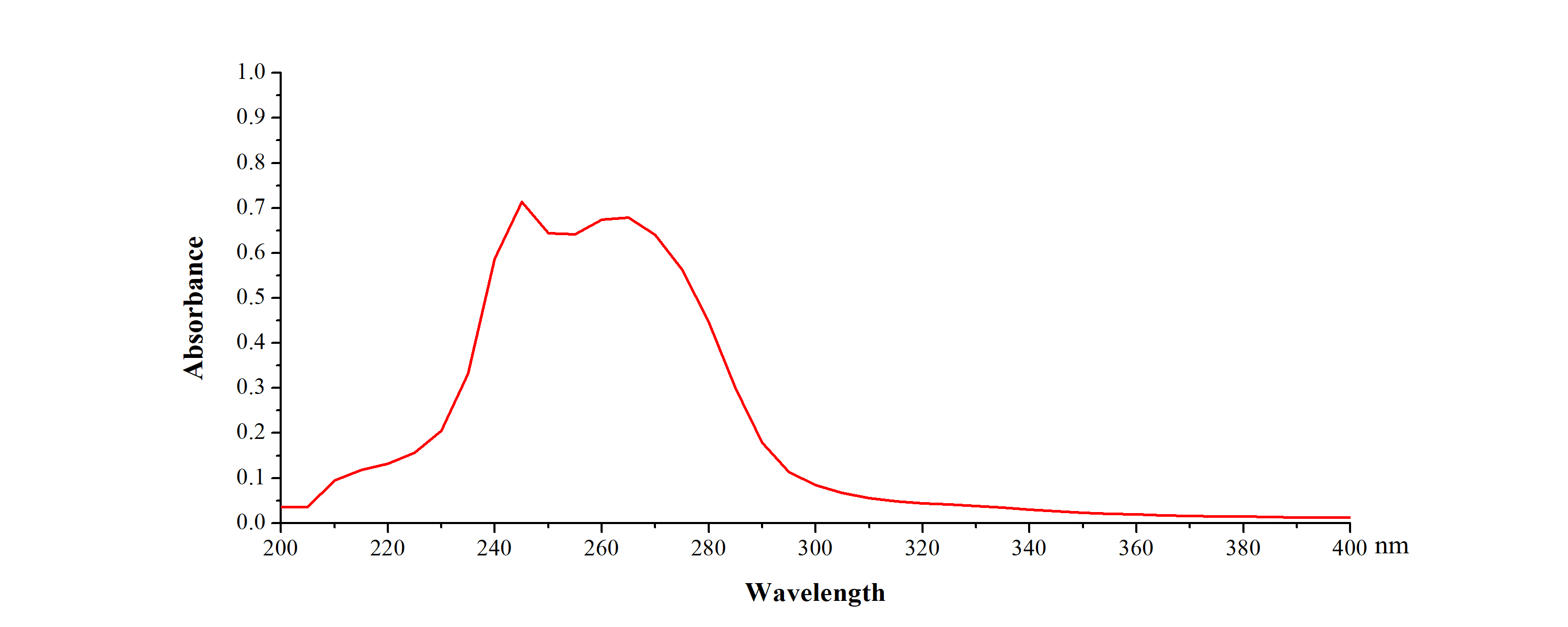
**

S10. UV spectrum of **1**


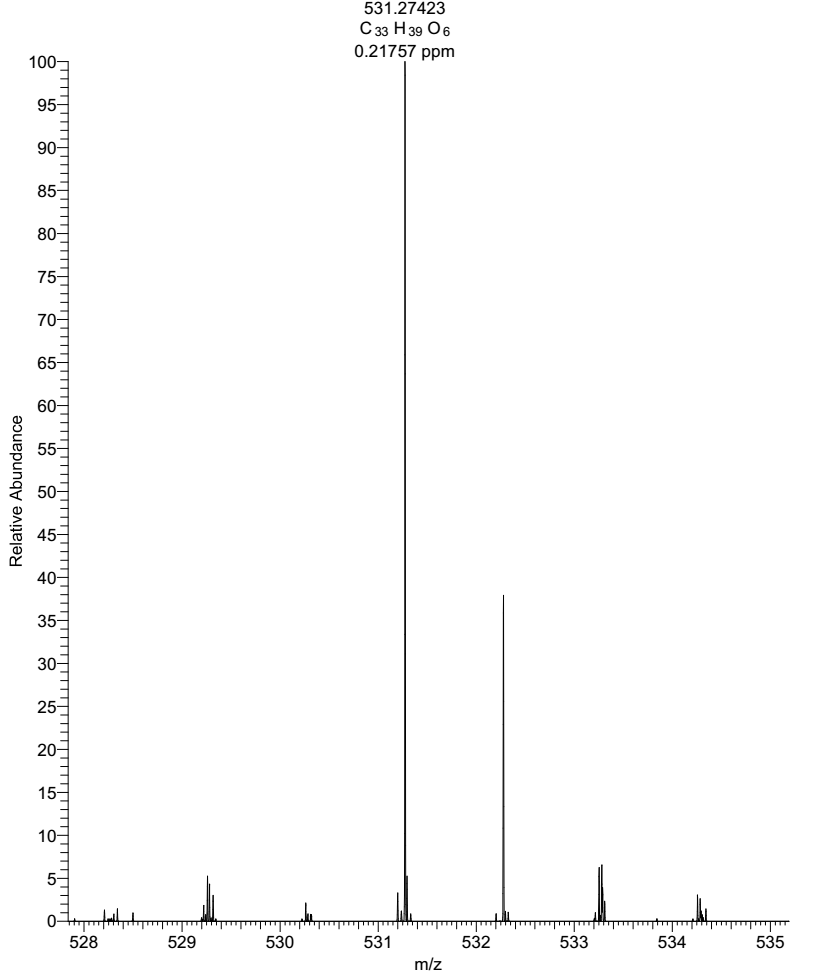


S11. HRESIMS spectrum of **1**


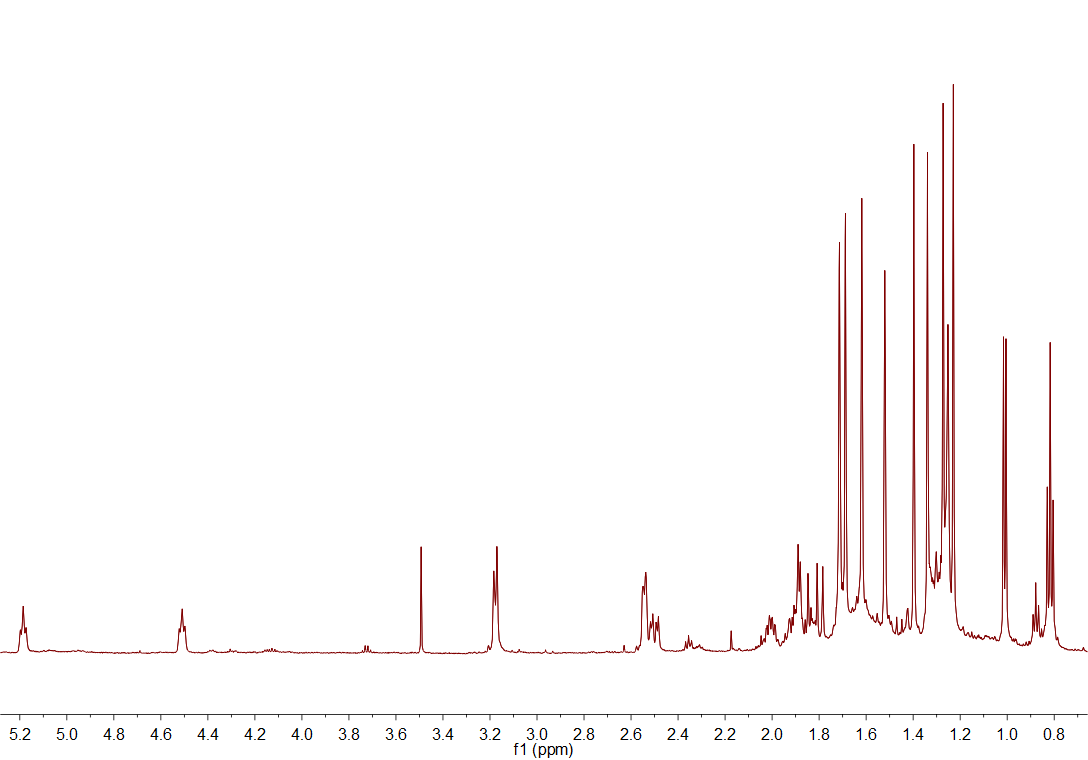


S12. ^1^H NMR spectrum of compound **2** (CDCl_3_, 600 MHz)


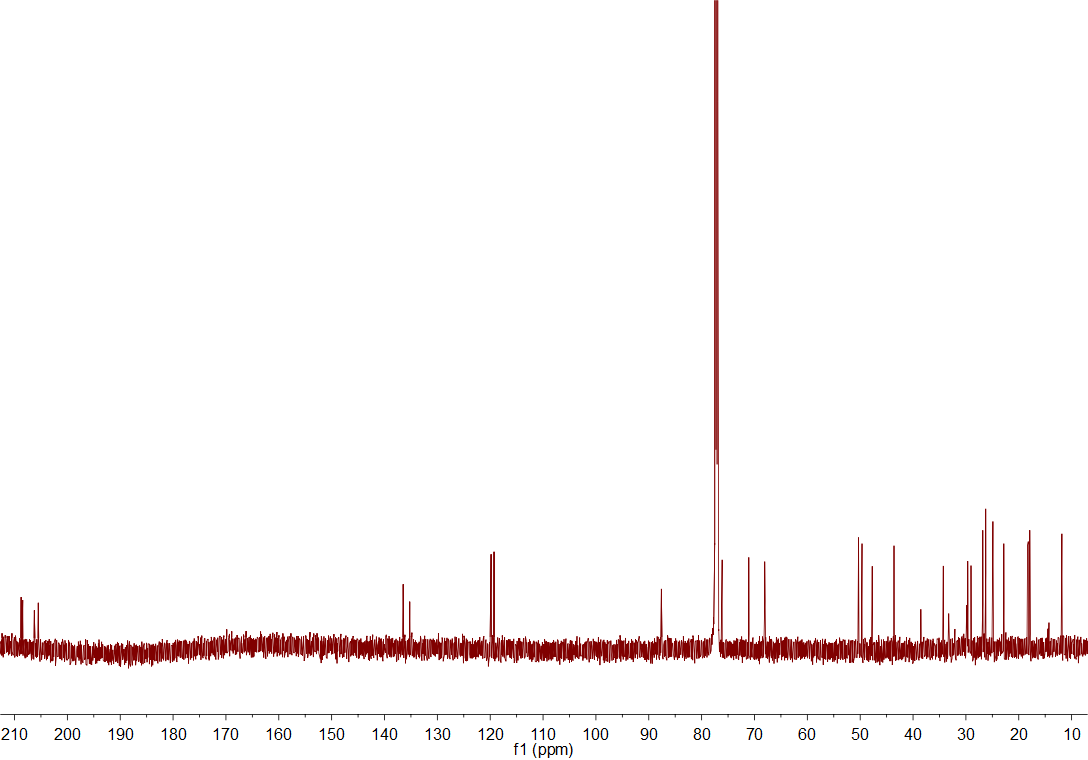


S13. ^13^C NMR spectrum of compound **2** (CDCl_3_, 150 MHz)


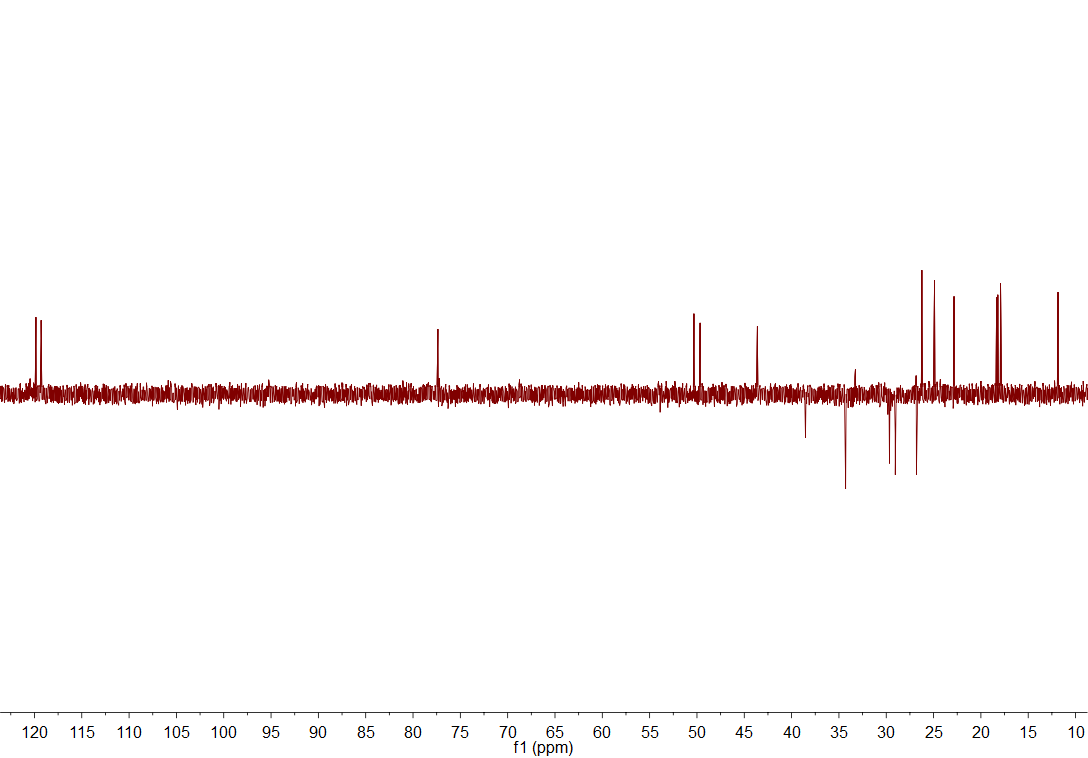


S14. DEPT 135° spectrum of compound **2** (CDCl_3_, 150 MHz)


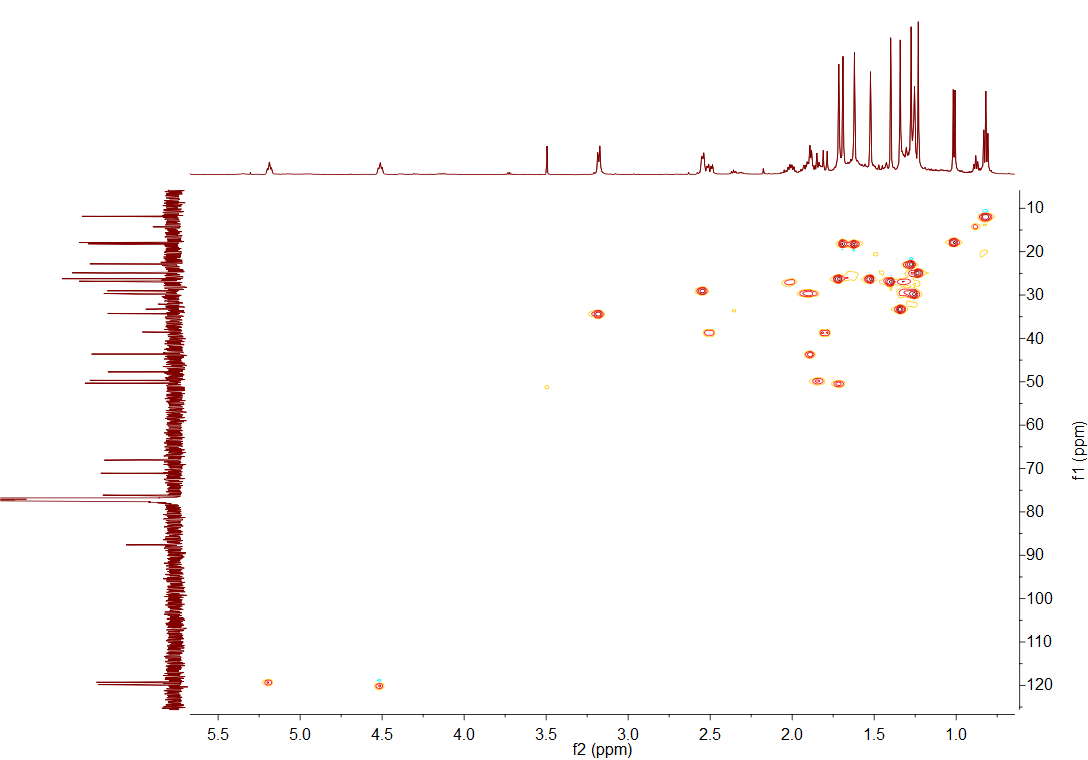


S15. HSQC spectrum of compound **2** (CDCl_3_)


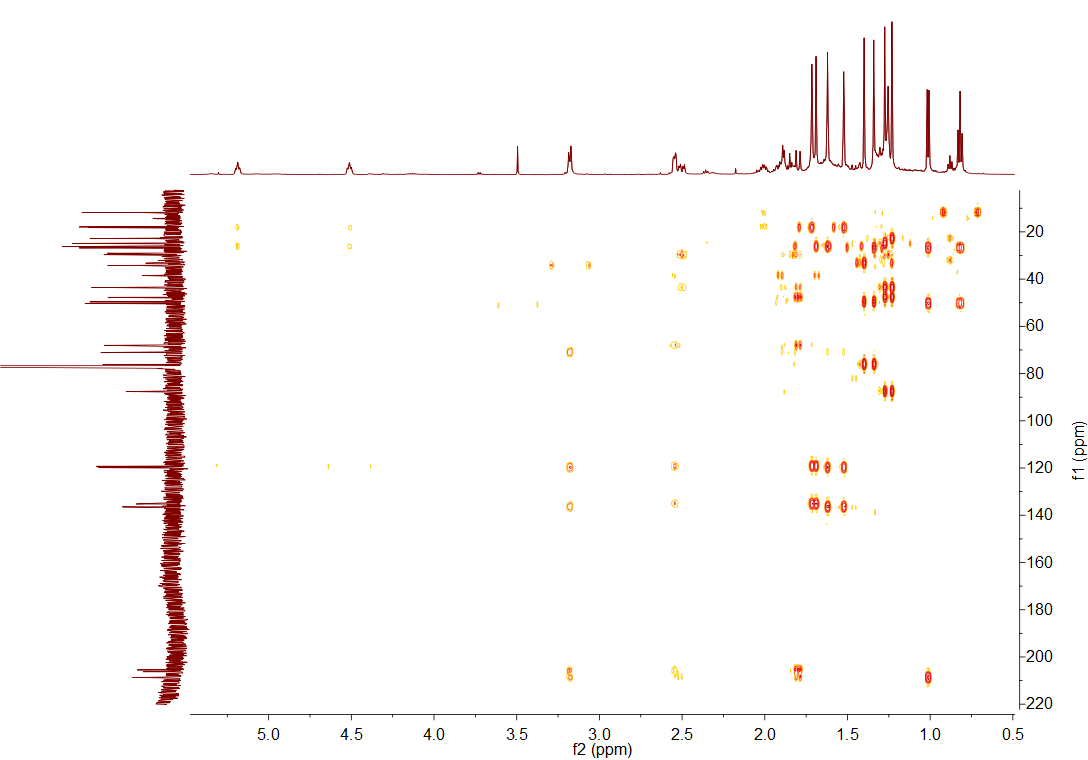


S16. HMBC spectrum of compound **2** (CDCl_3_)


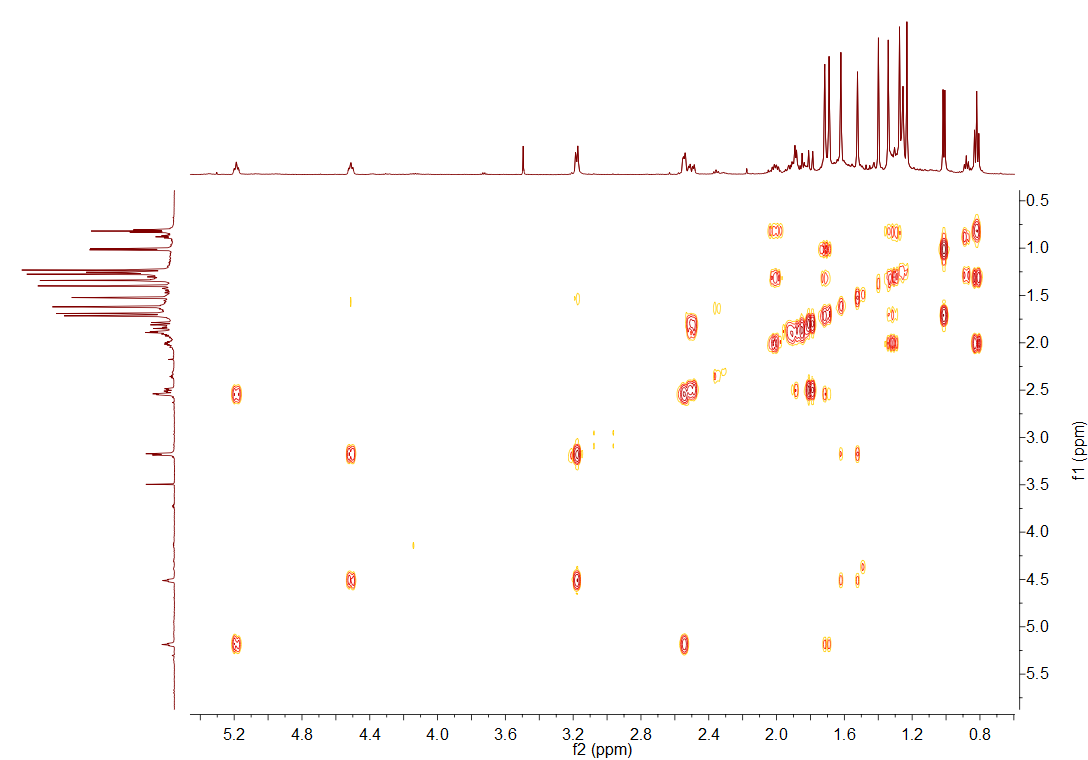


S17. ^1^H-^1^H COSY spectrum of compound **2** (CDCl_3_)


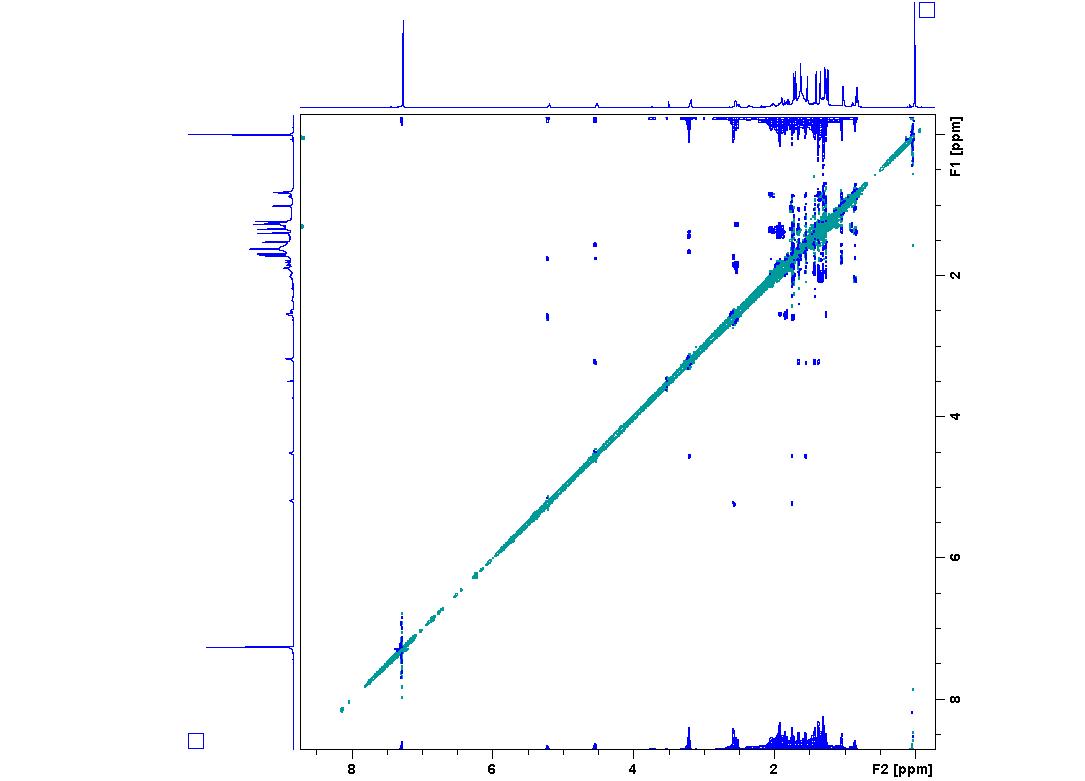


S18. ROESY spectrum of compound **2** (CDCl_3_)

**
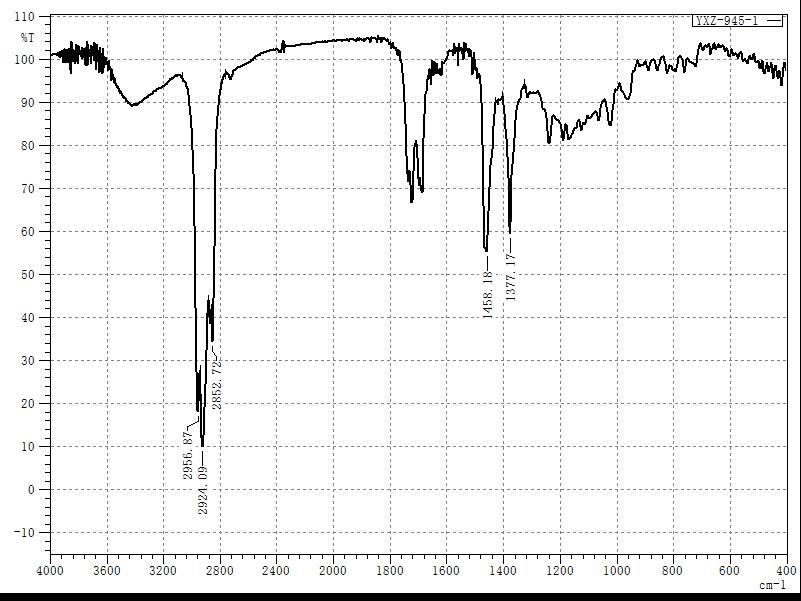
**

S19. IR spectrum of **2**

**
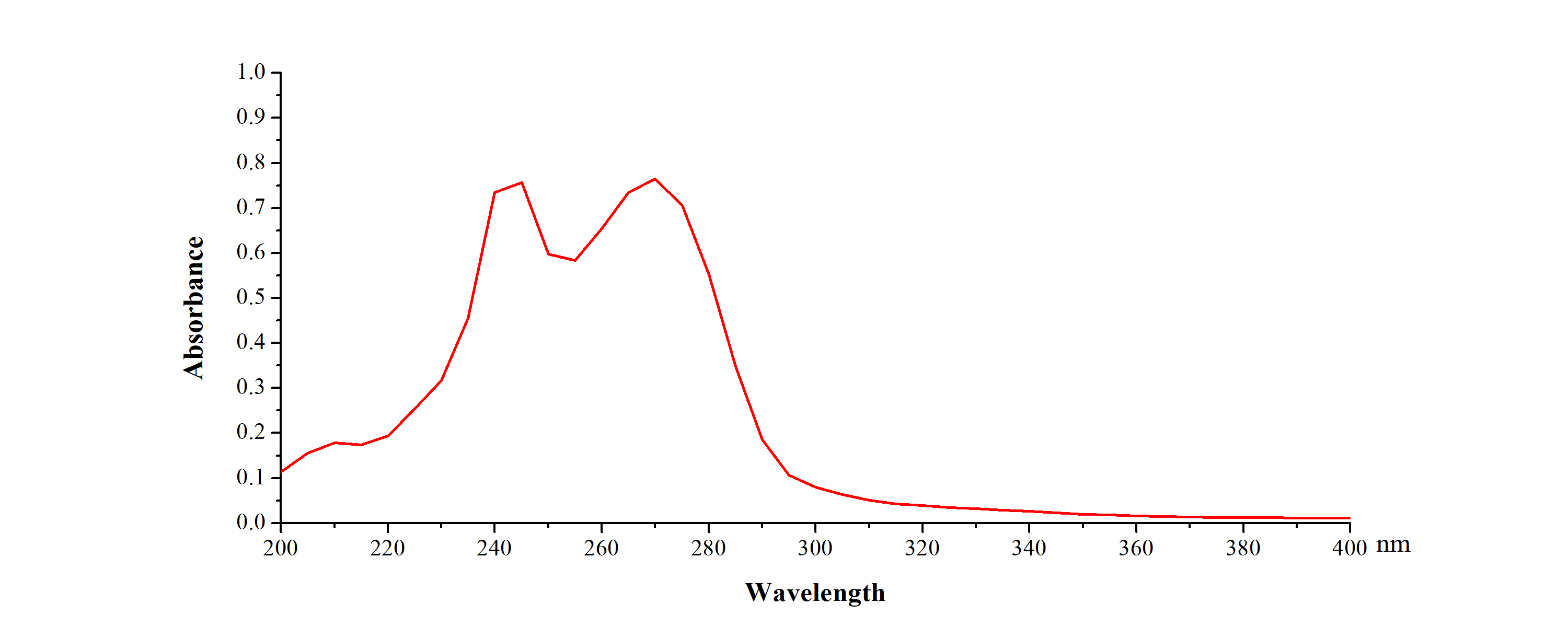
**

S20. UV spectrum of **2**


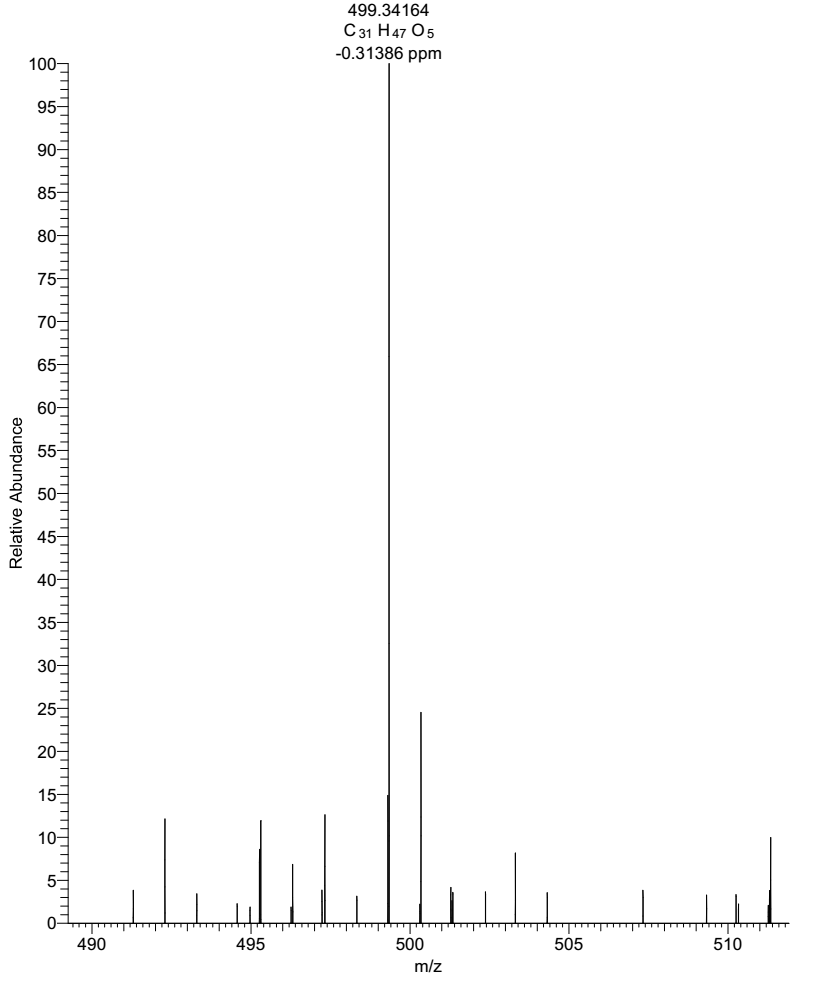


S21. HRESIMS spectrum of **2**


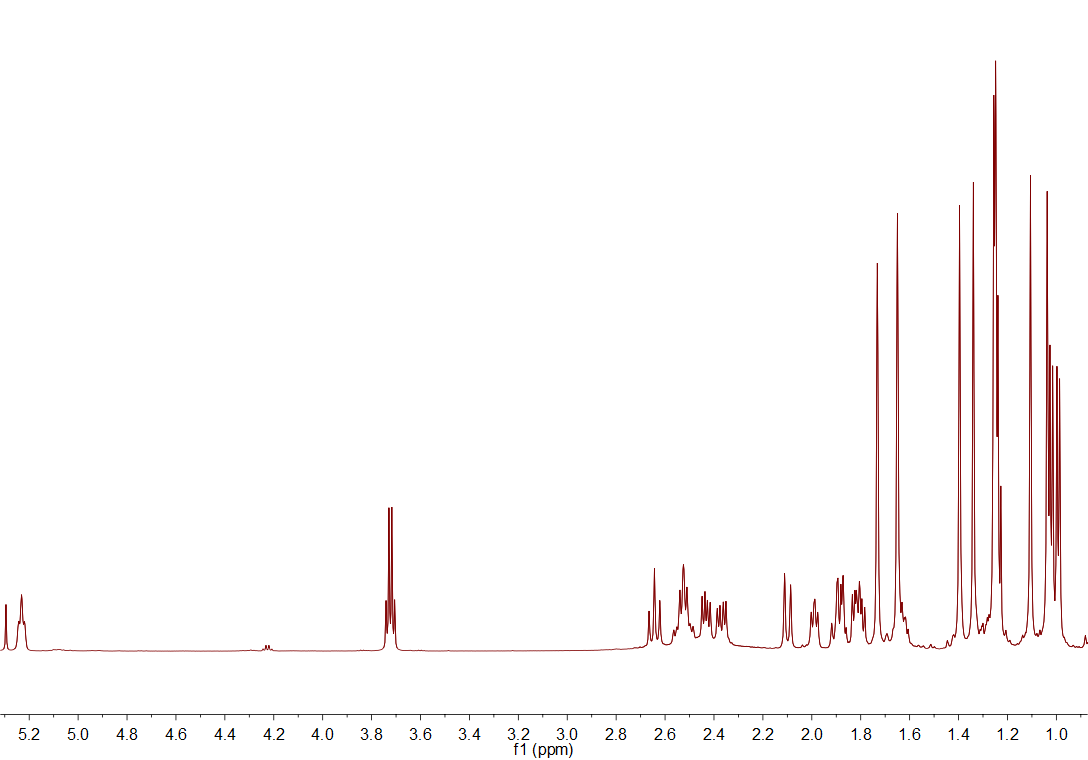


S22. ^1^H NMR spectrum of compound **3** (CDCl_3_, 600 MHz)


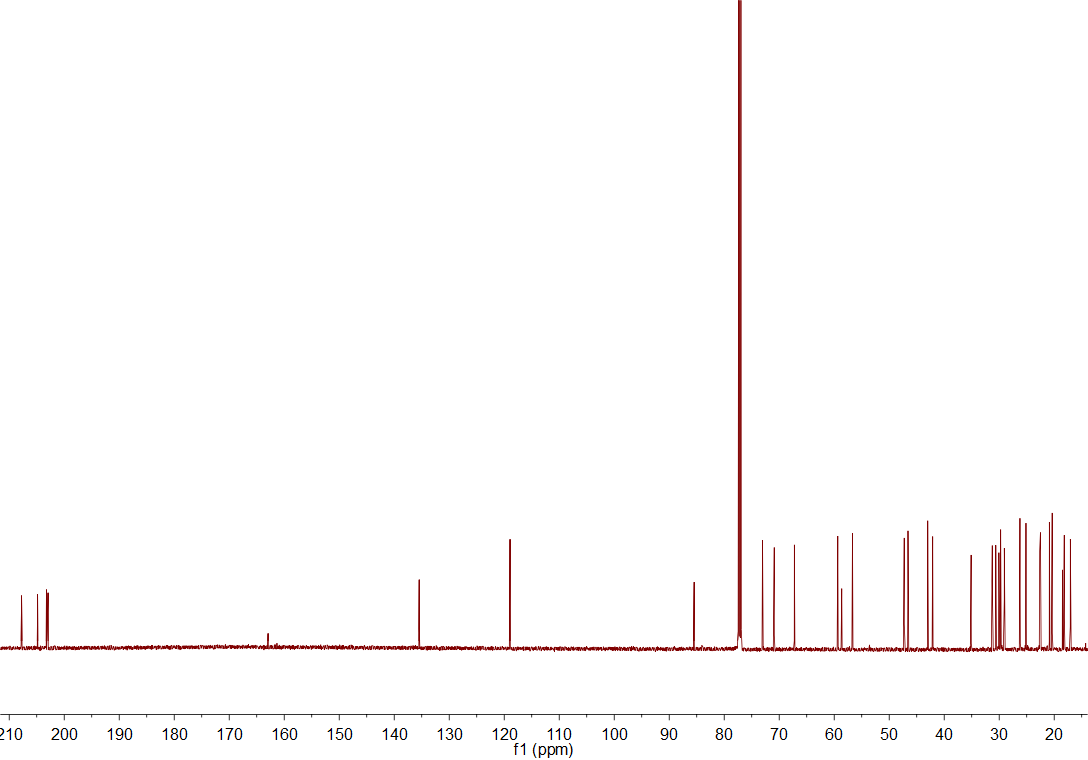


S23. ^13^C NMR spectrum of compound **3** (CDCl_3_, 150 MHz)


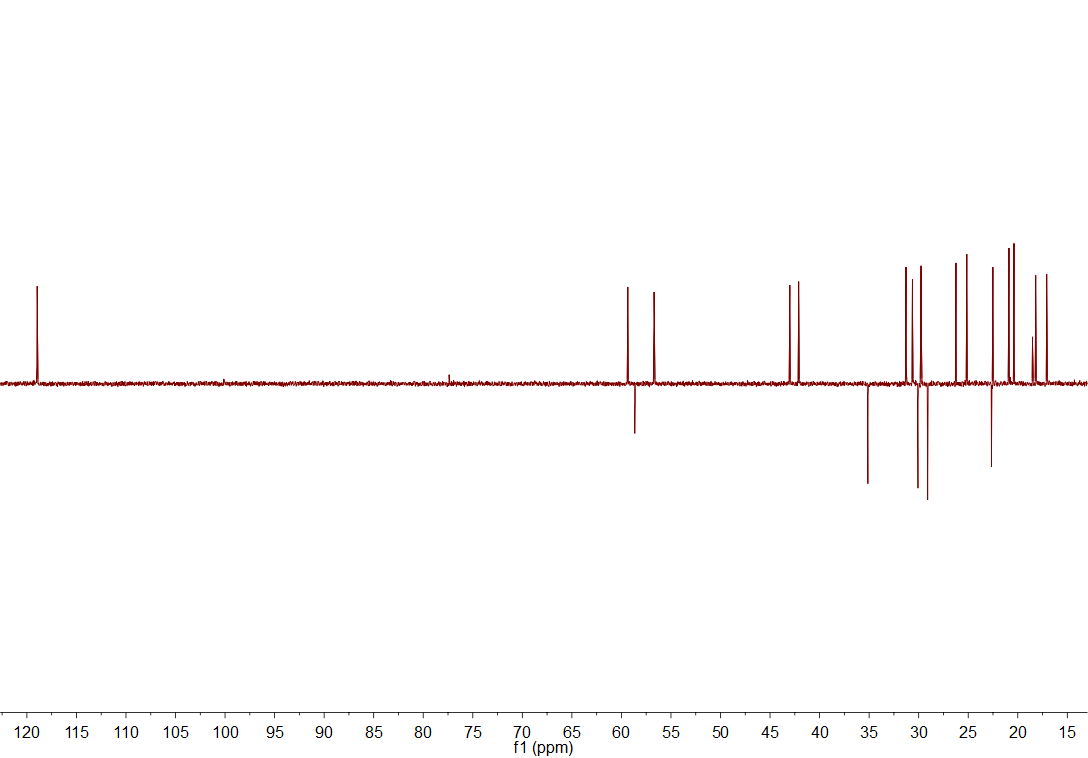


S24. DEPT 135° spectrum of compound **3** (CDCl_3_, 150 MHz)


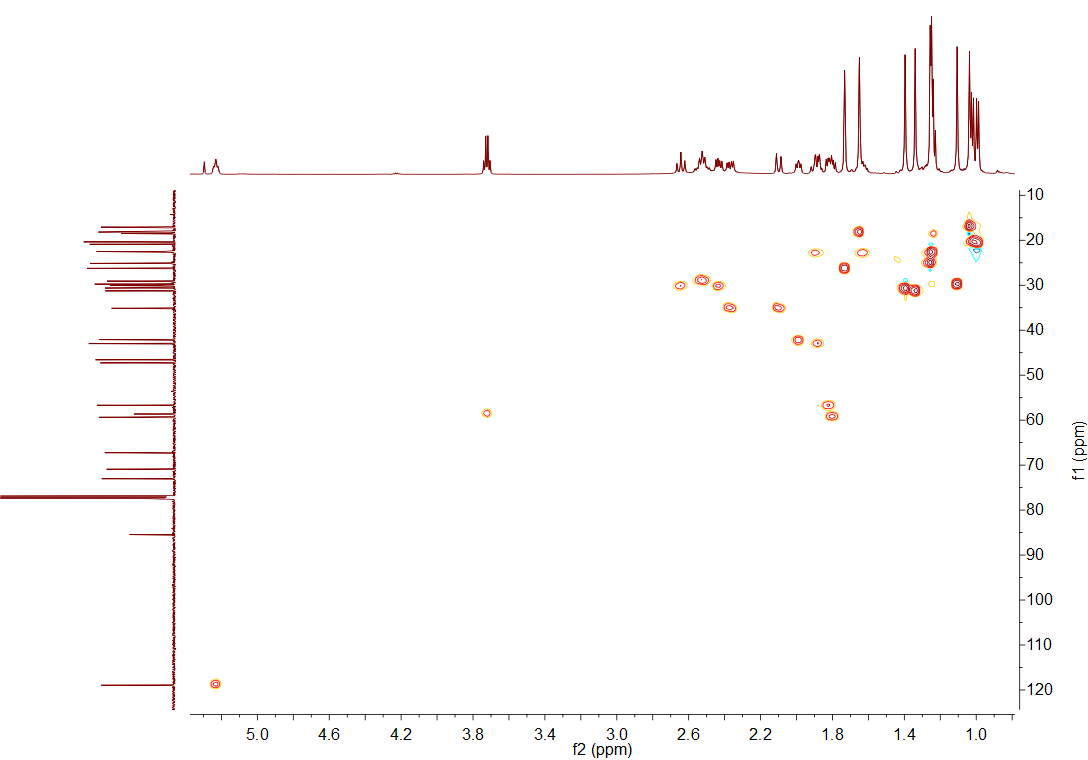


S25. HSQC spectrum of compound **3** (CDCl_3_)


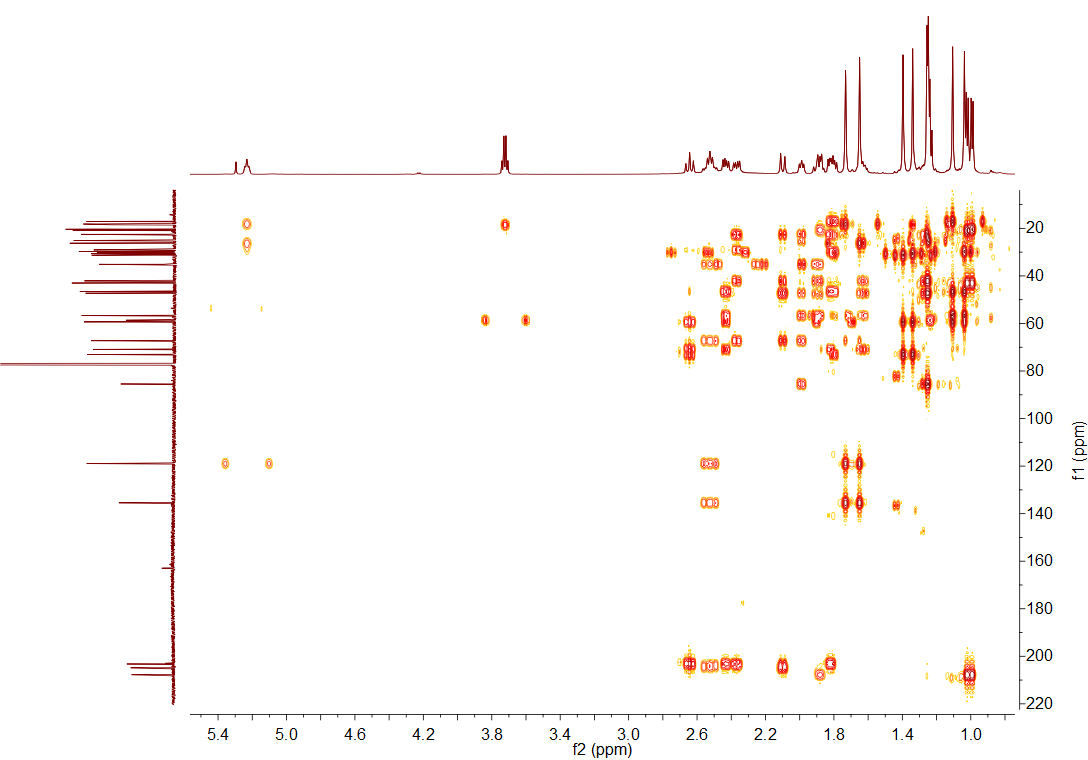


S26. HMBC spectrum of compound **3** (CDCl_3_)


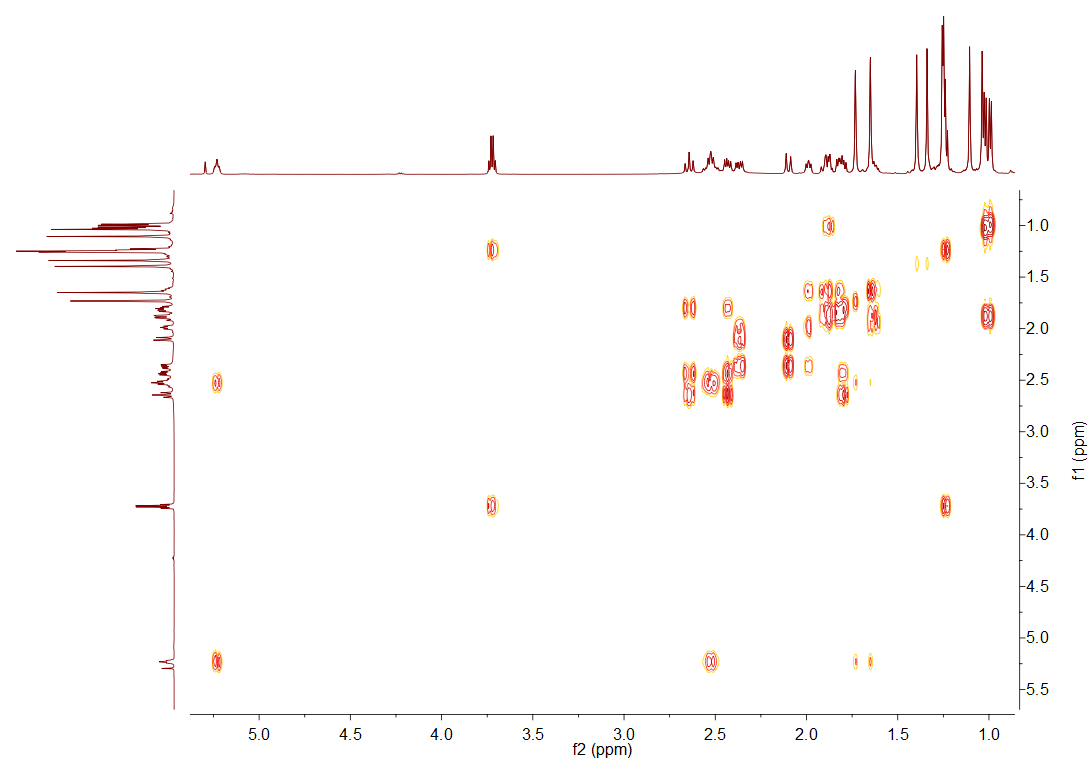


S27. ^1^H-^1^H COSY spectrum of compound **3** (CDCl_3_)


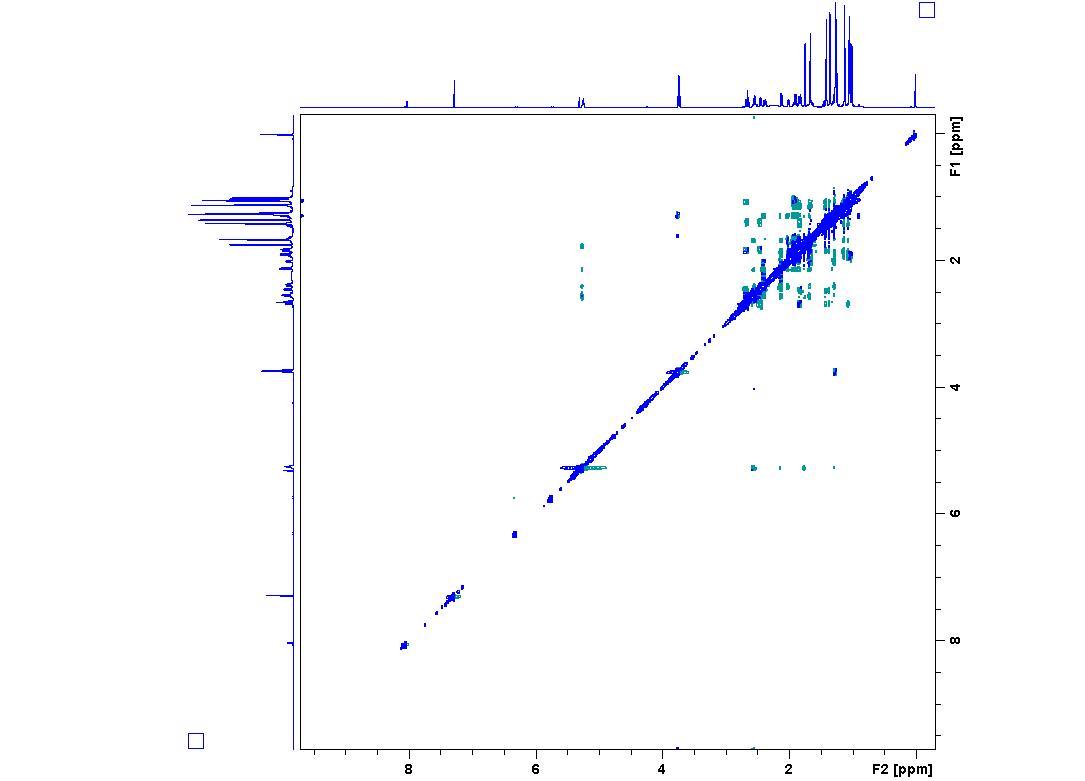


S28. ROESY spectrum of compound **3** (CDCl_3_)


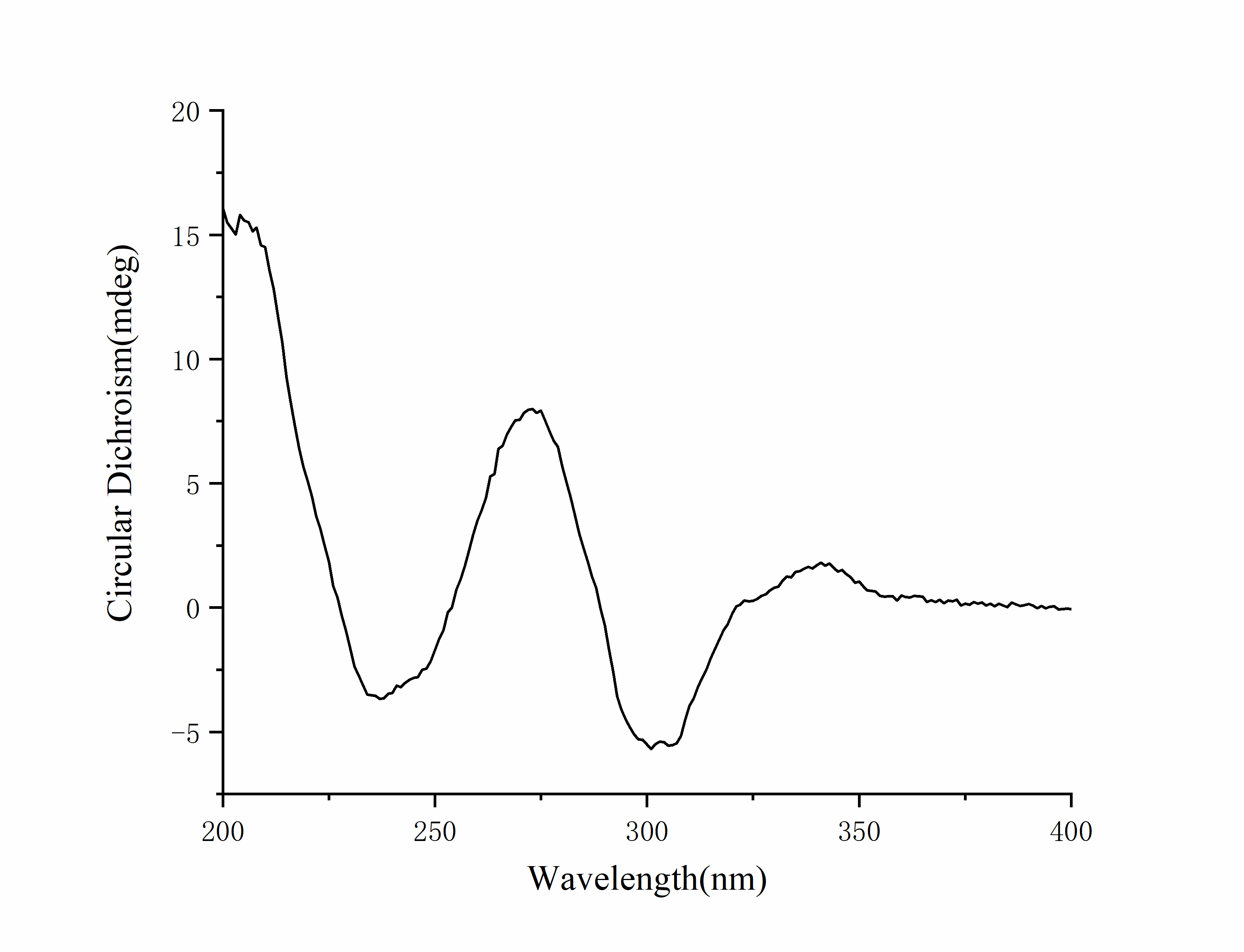


S29. CD spectrum of **3**

**
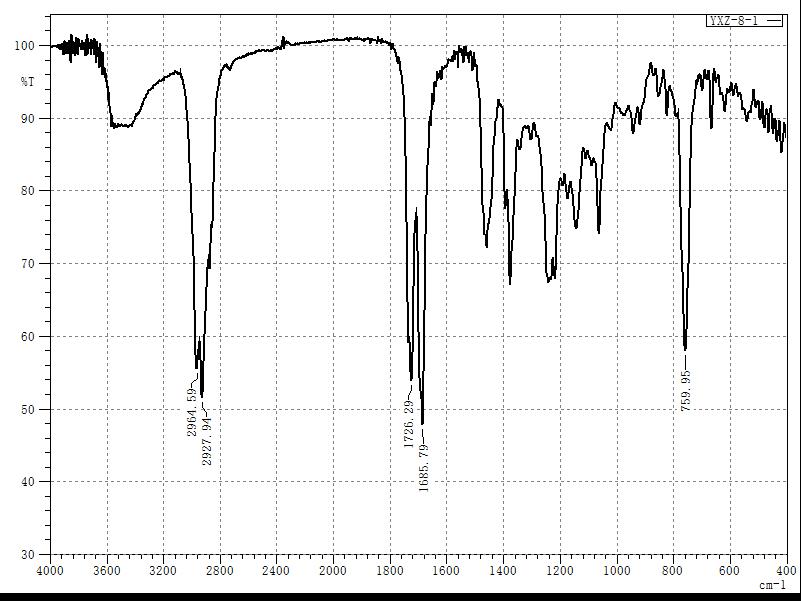
**

S30. IR spectrum of **3**

**
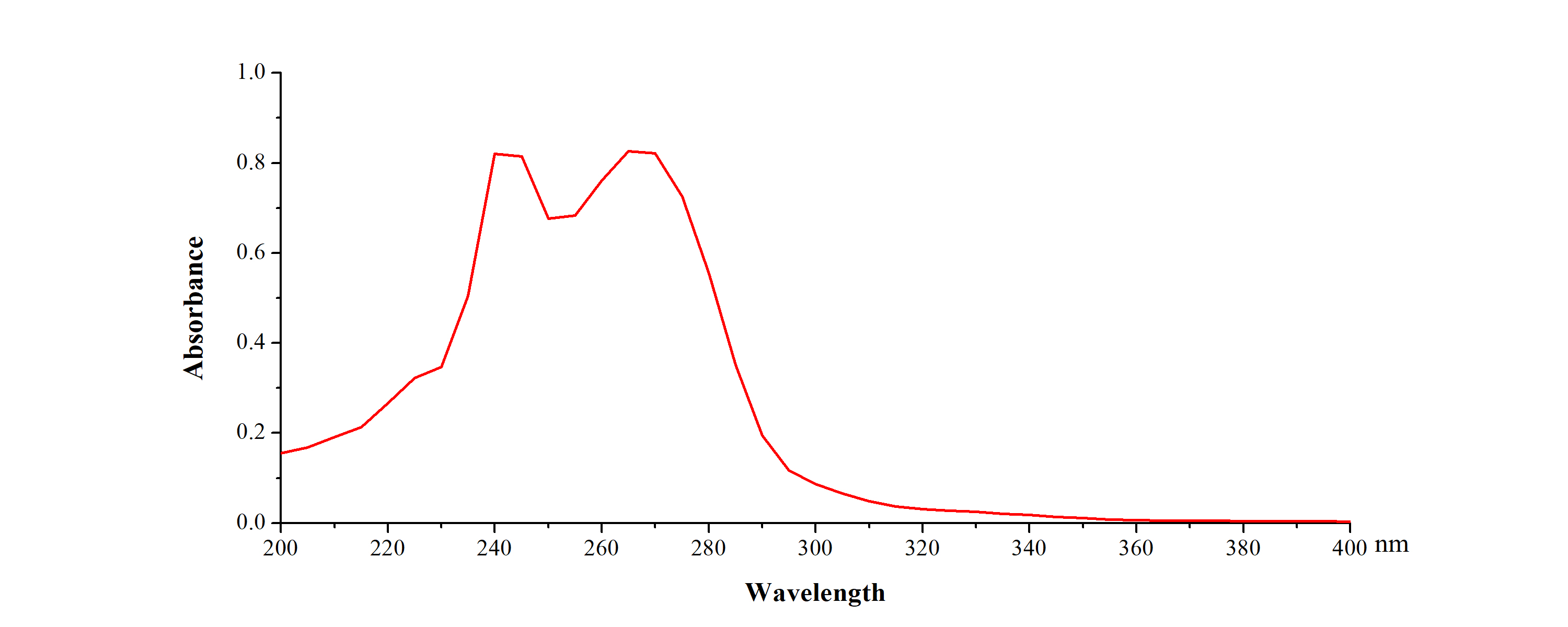
**

S31. UV spectrum of **3**


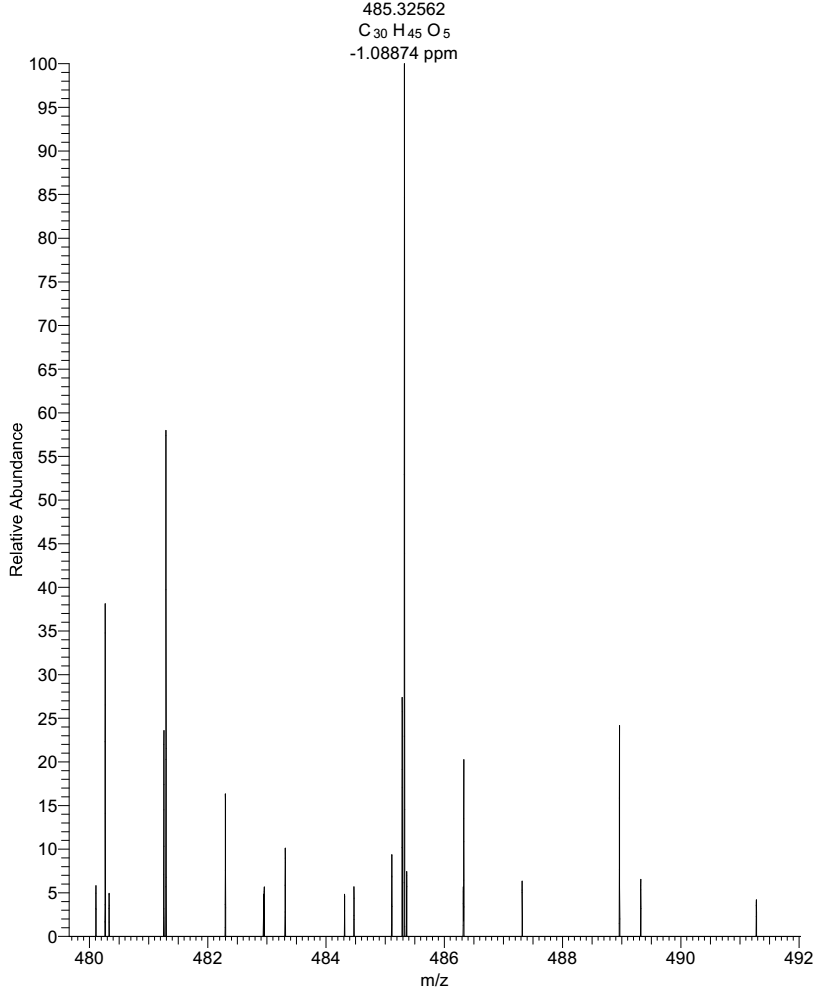


S32. HRESIMS spectrum of **3**


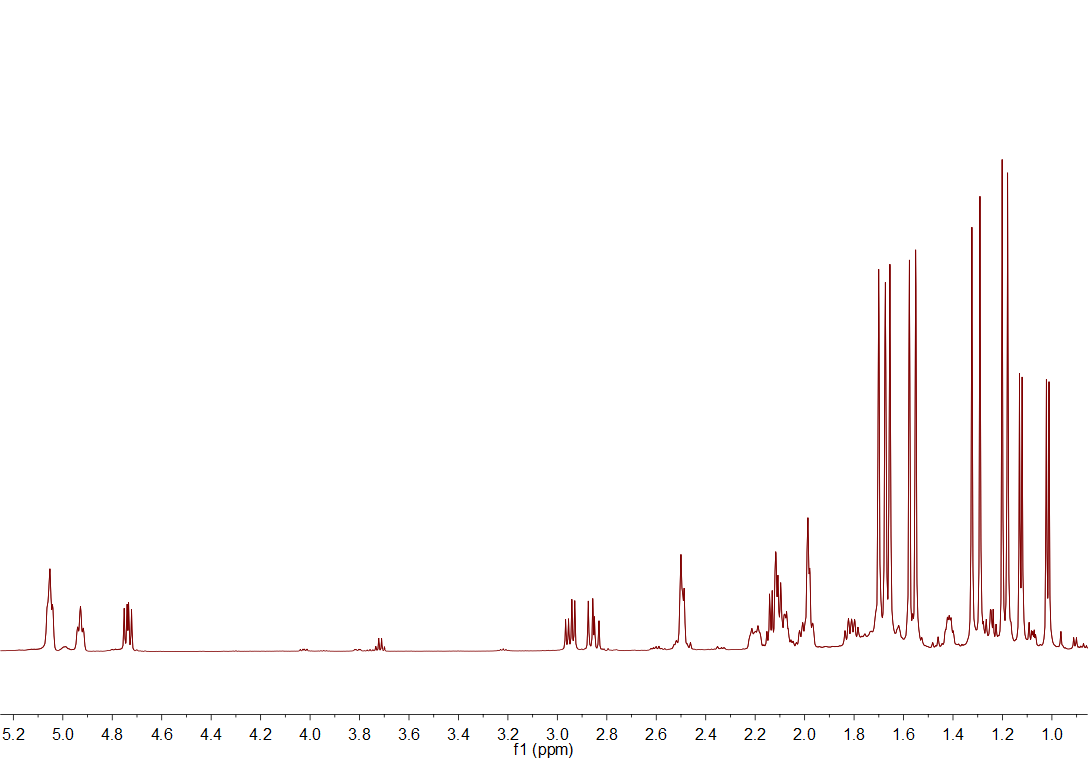


S33. ^1^H NMR spectrum of compound **4** (CDCl_3_, 600 MHz)


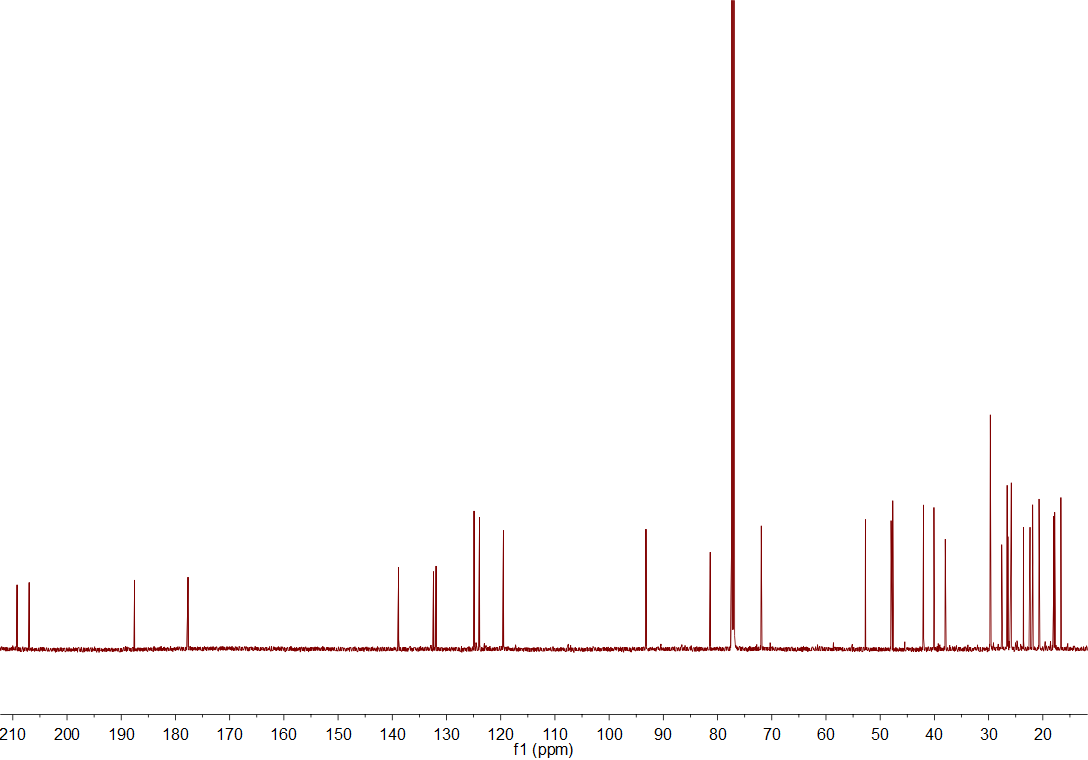


S34. ^13^C NMR spectrum of compound **4** (CDCl_3_, 150 MHz)


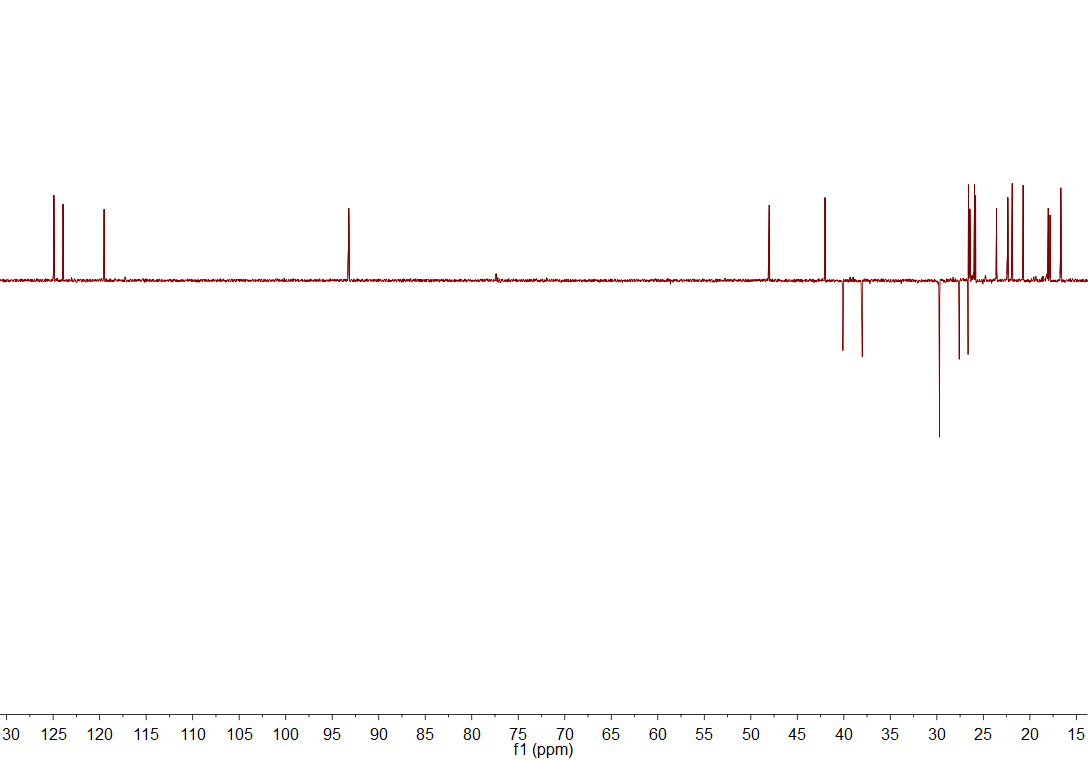


S35. DEPT 135° spectrum of compound **4** (CDCl_3_, 150 MHz)


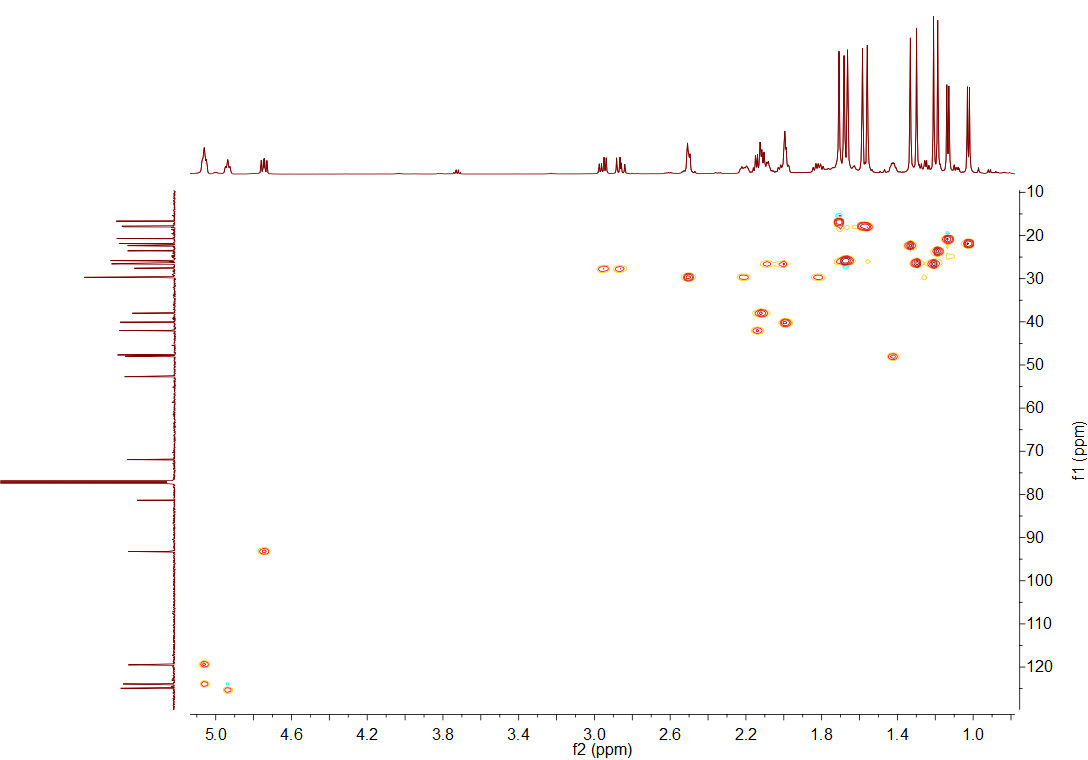


S36. HSQC spectrum of compound **4** (CDCl_3_)


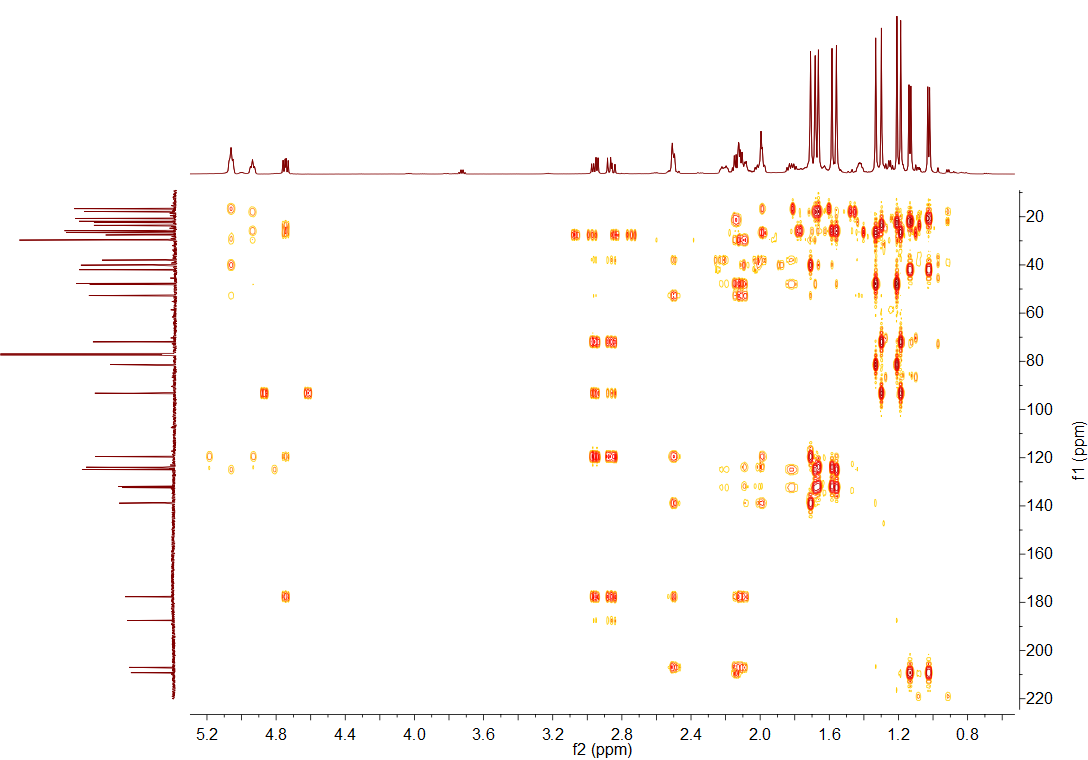


S37. HMBC spectrum of compound **4** (CDCl_3_)


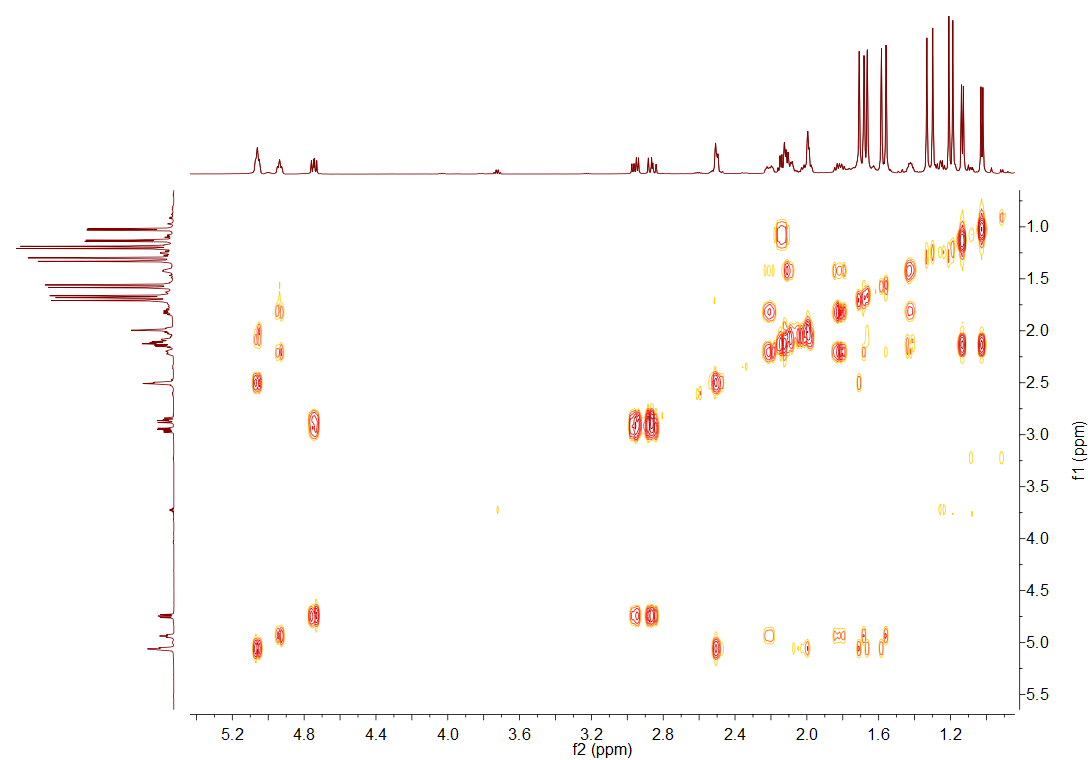


S38. ^1^H-^1^H COSY spectrum of compound **4** (CDCl_3_)


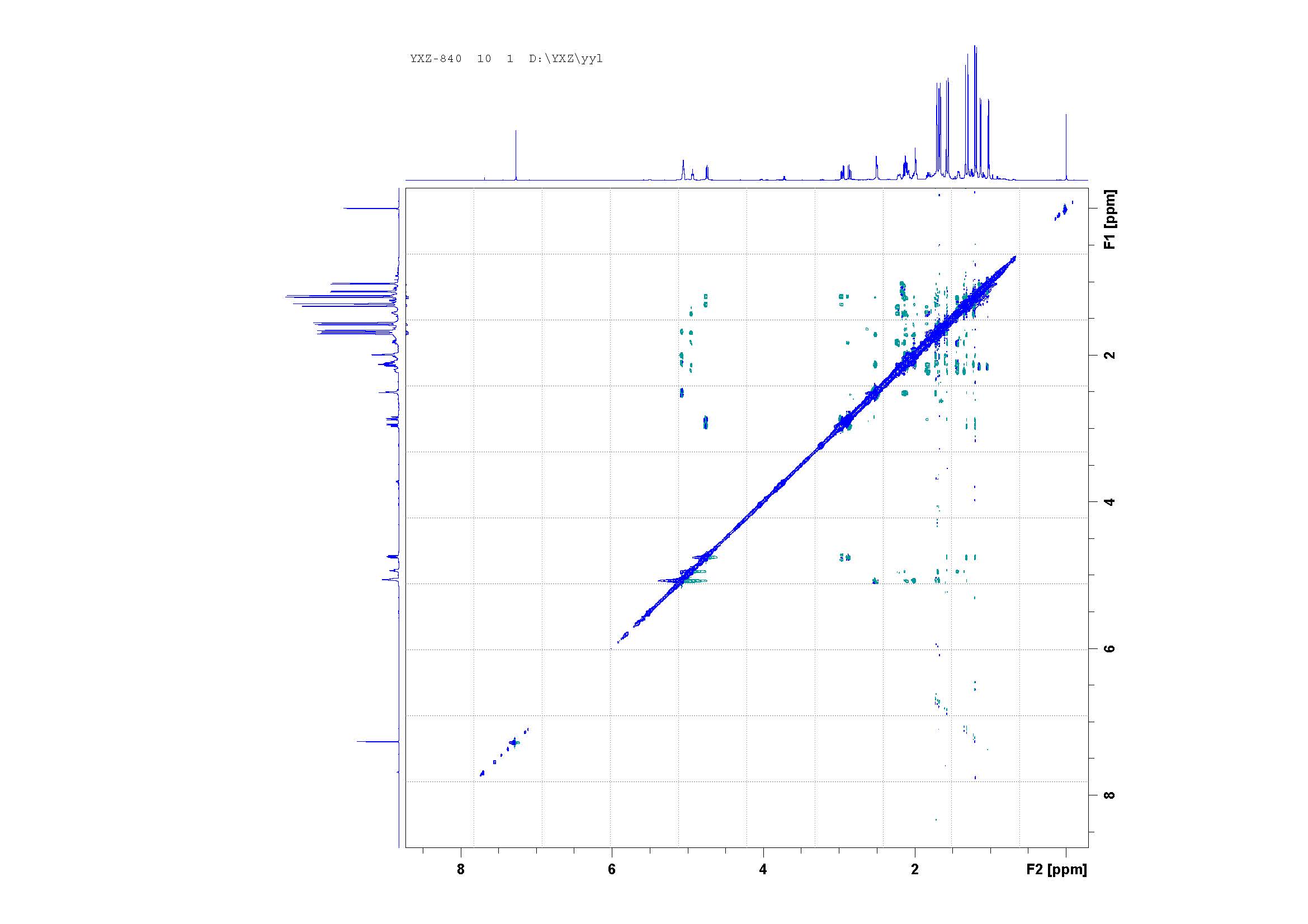


S39. ROESY spectrum of compound **4** (CDCl_3_)


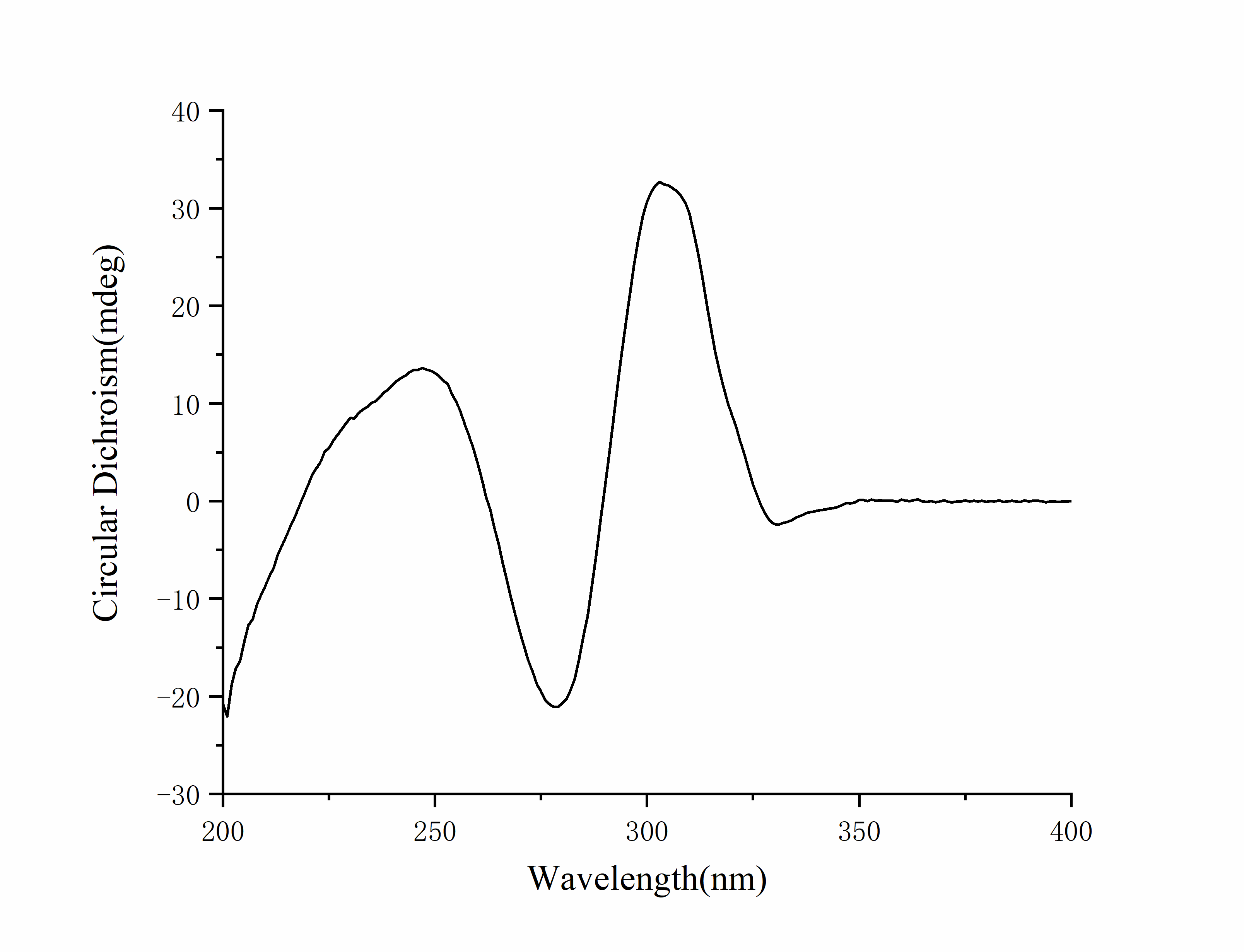


S40. CD spectrum of **4**

**
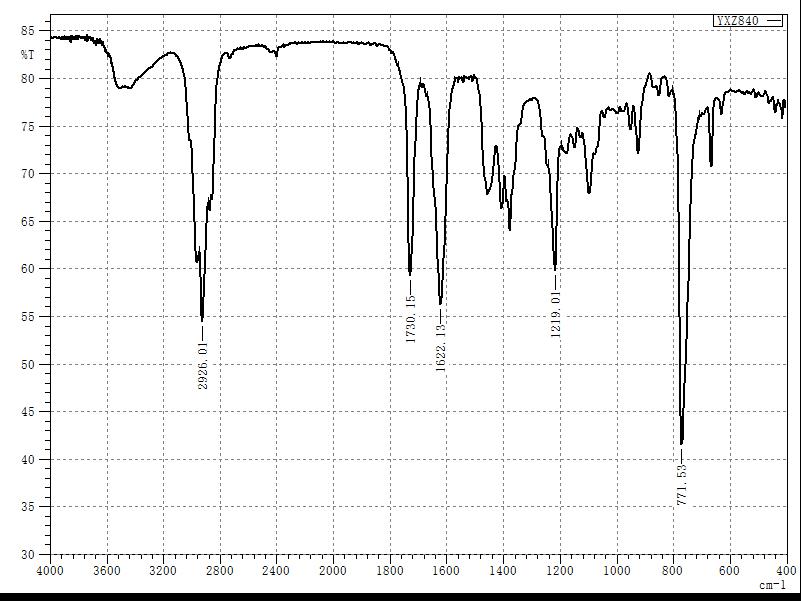
**

S41. IR spectrum of **4**

**
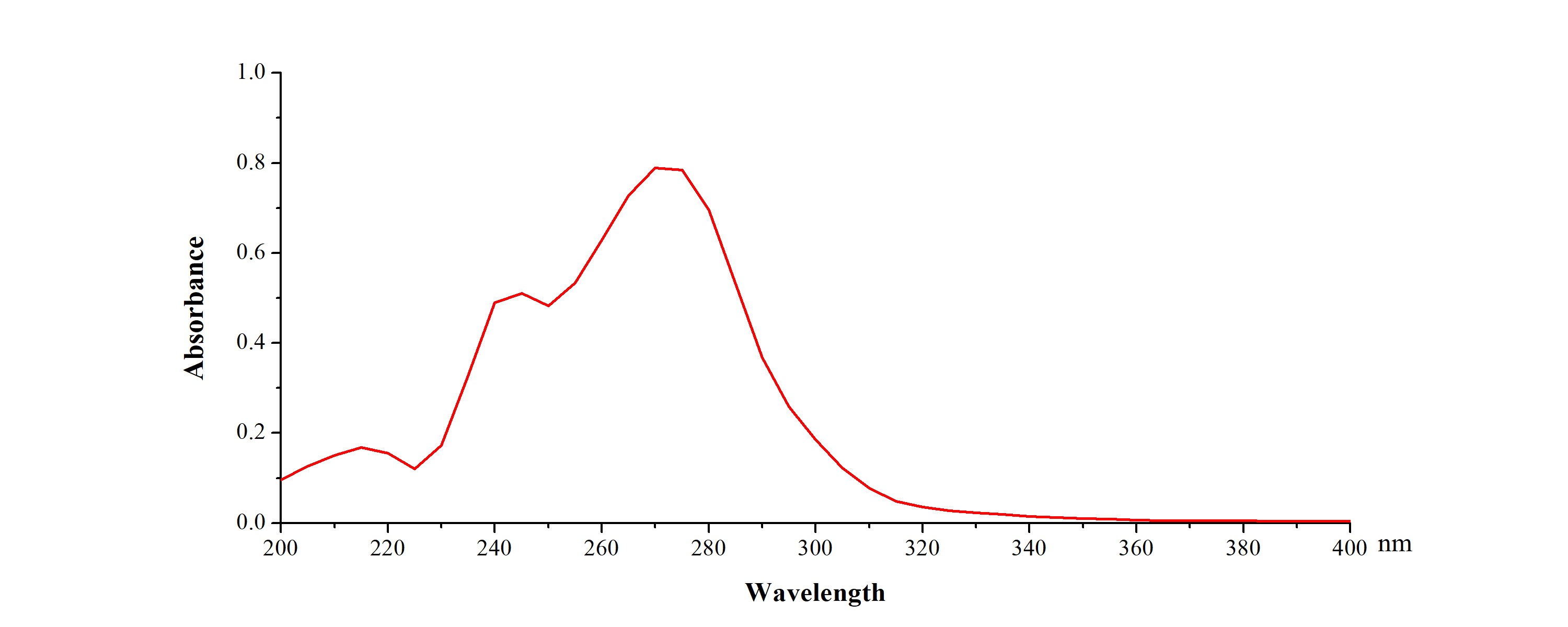
**

S42. UV spectrum of **4**


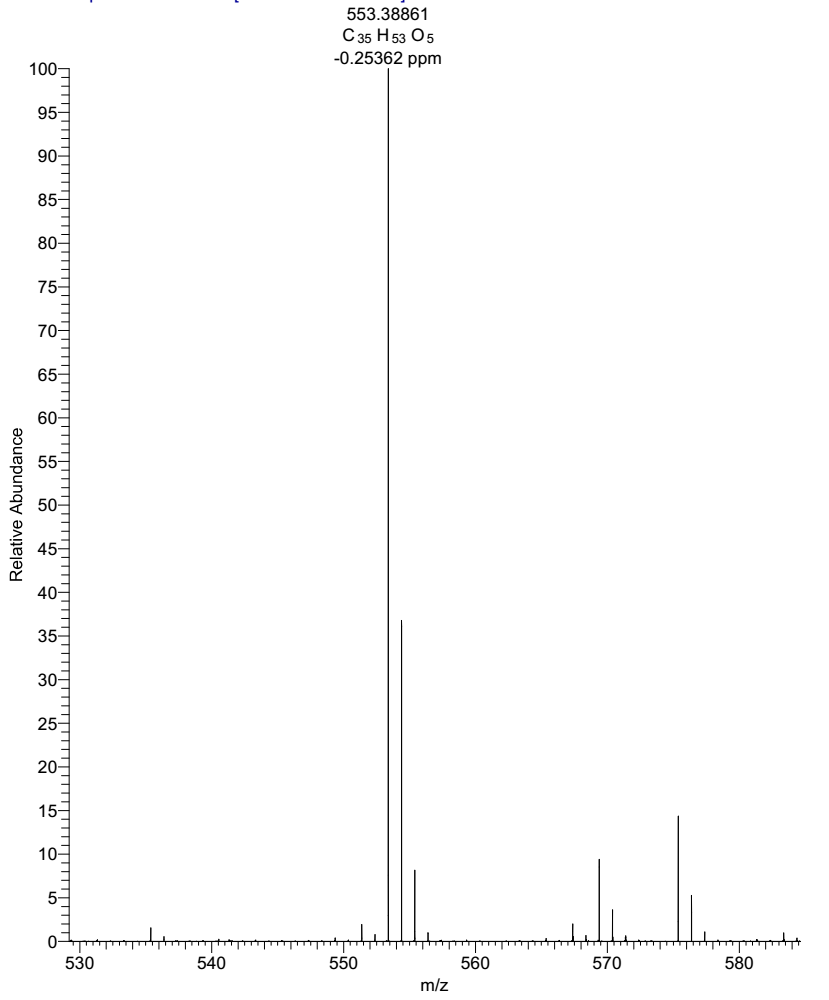


S43. HRESIMS spectrum of **4**


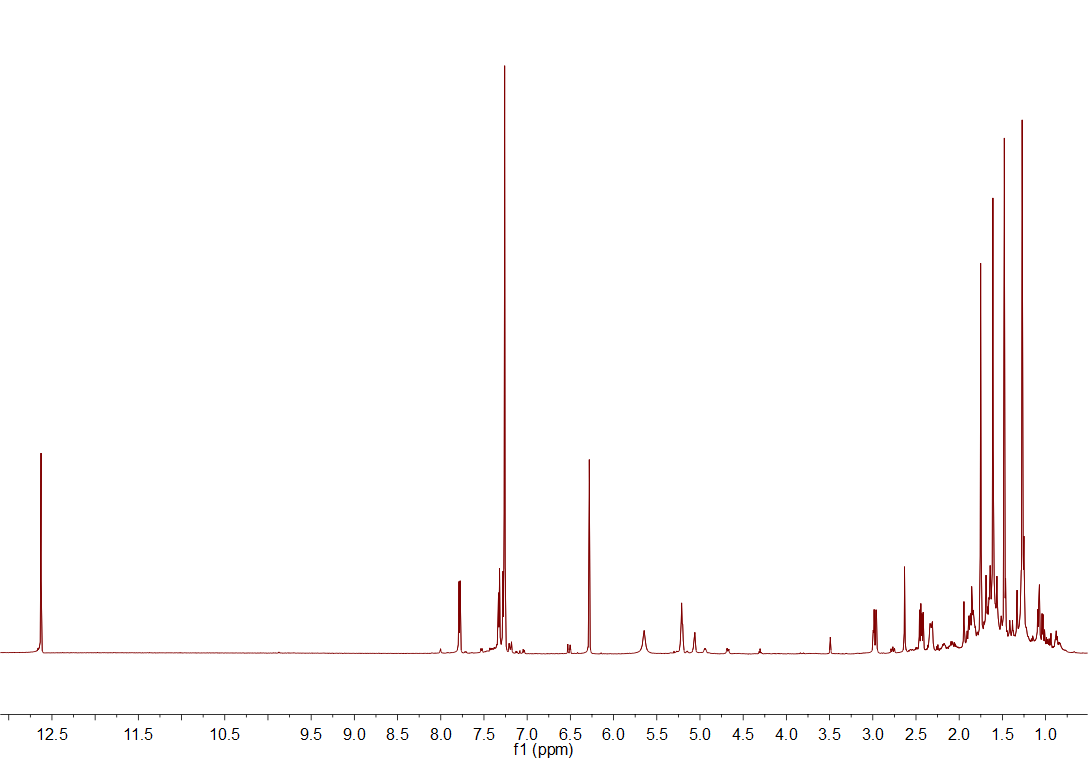


S44. ^1^H NMR spectrum of compound **5** (CDCl_3_, 600 MHz)


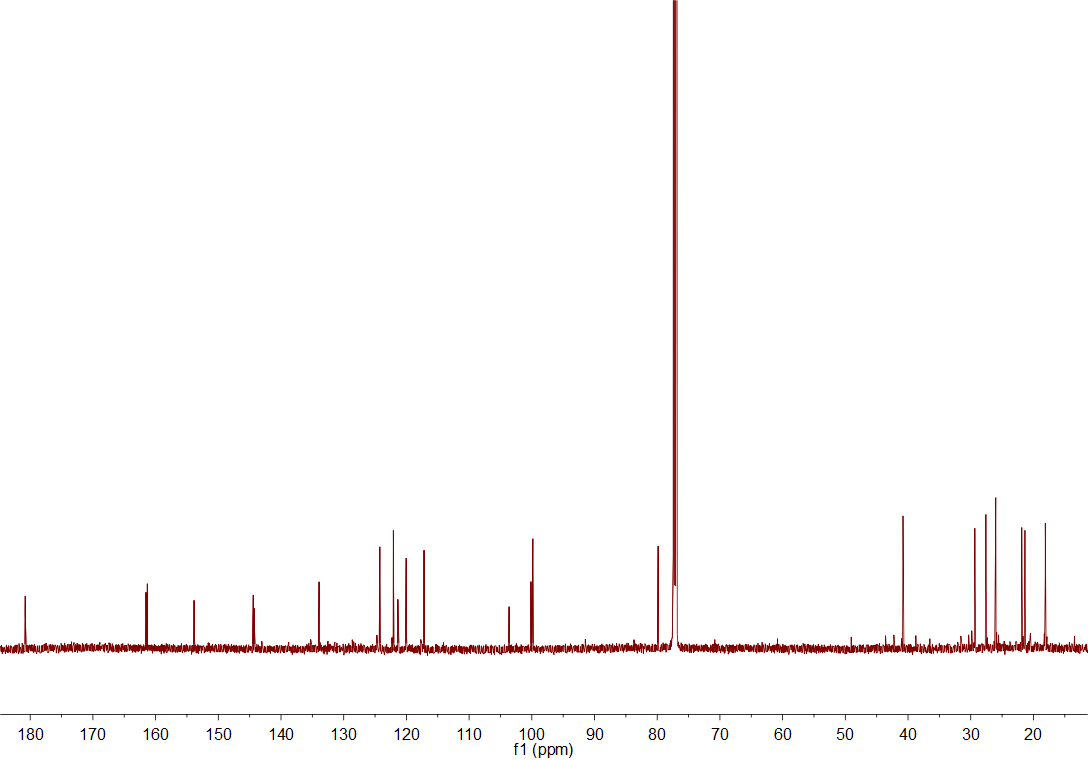


S45. ^13^C NMR spectrum of compound **5** (CDCl_3_, 150 MHz)


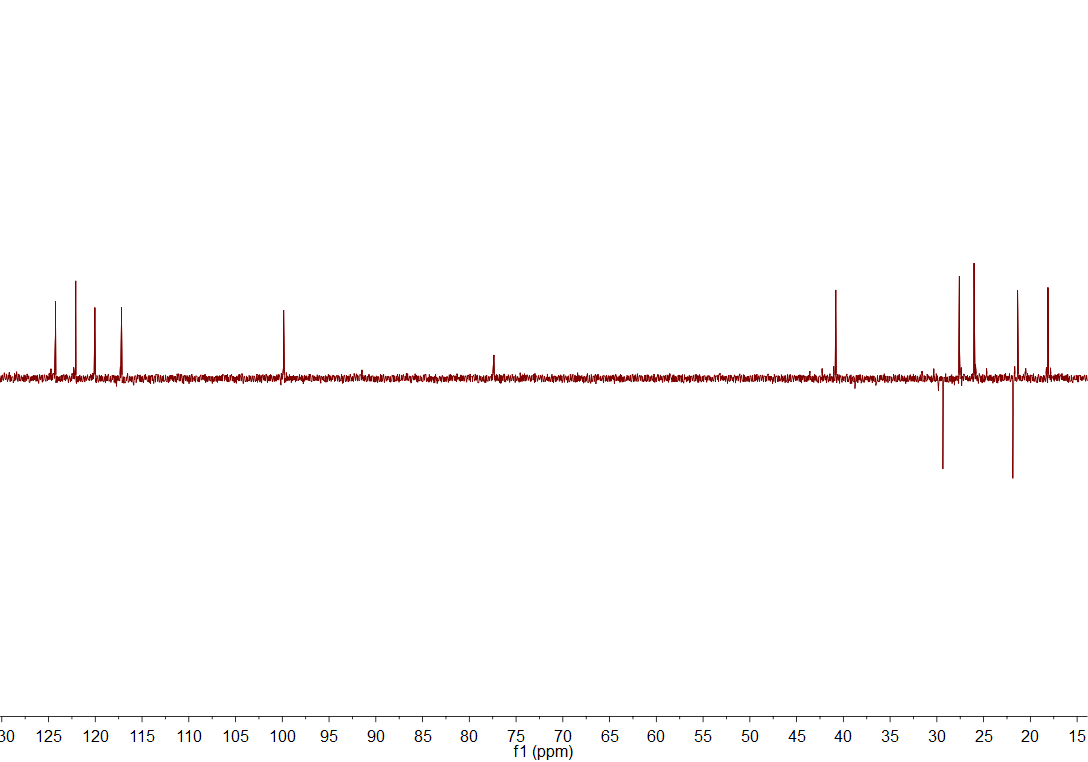


S46. DEPT 135° spectrum of compound **5** (CDCl_3_, 150 MHz)


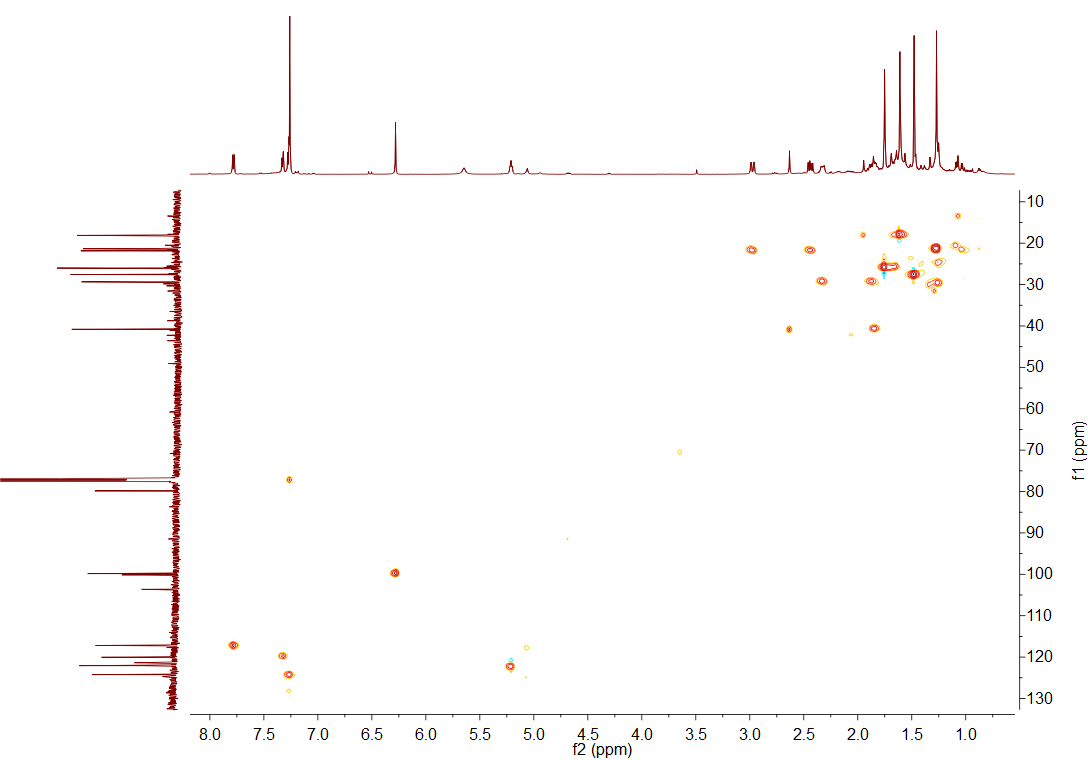


S47. HSQC spectrum of compound **5** (CDCl_3_)


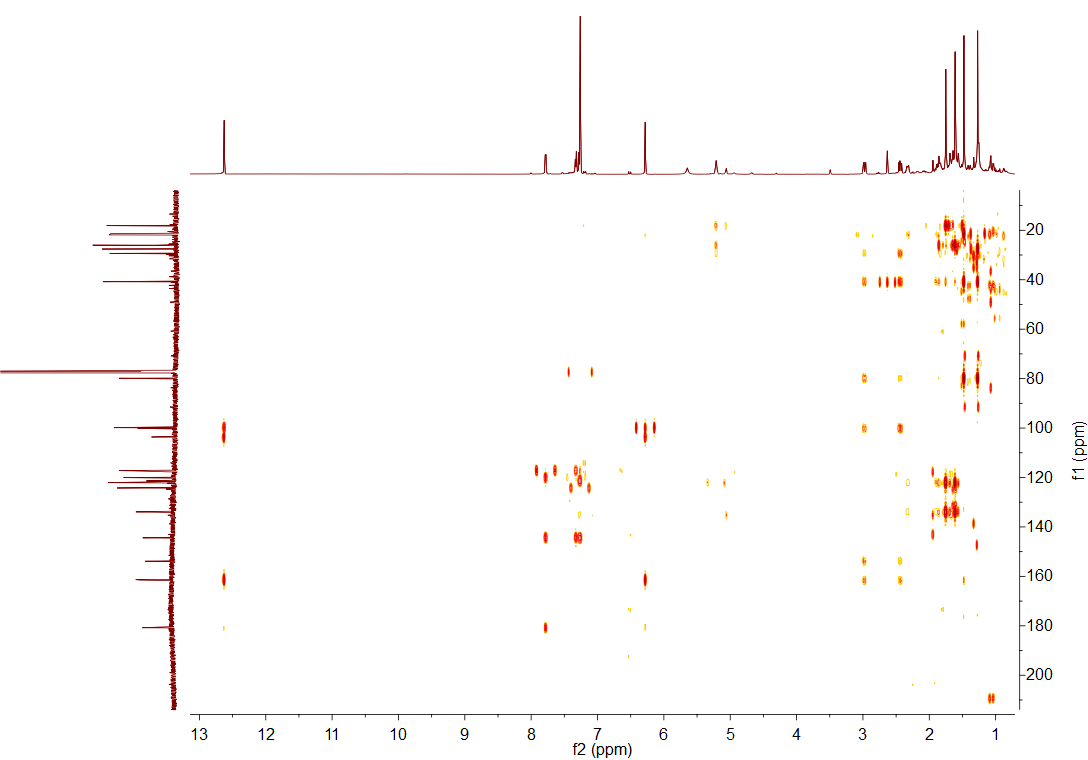


S48. HMBC spectrum of compound **5** (CDCl_3_)


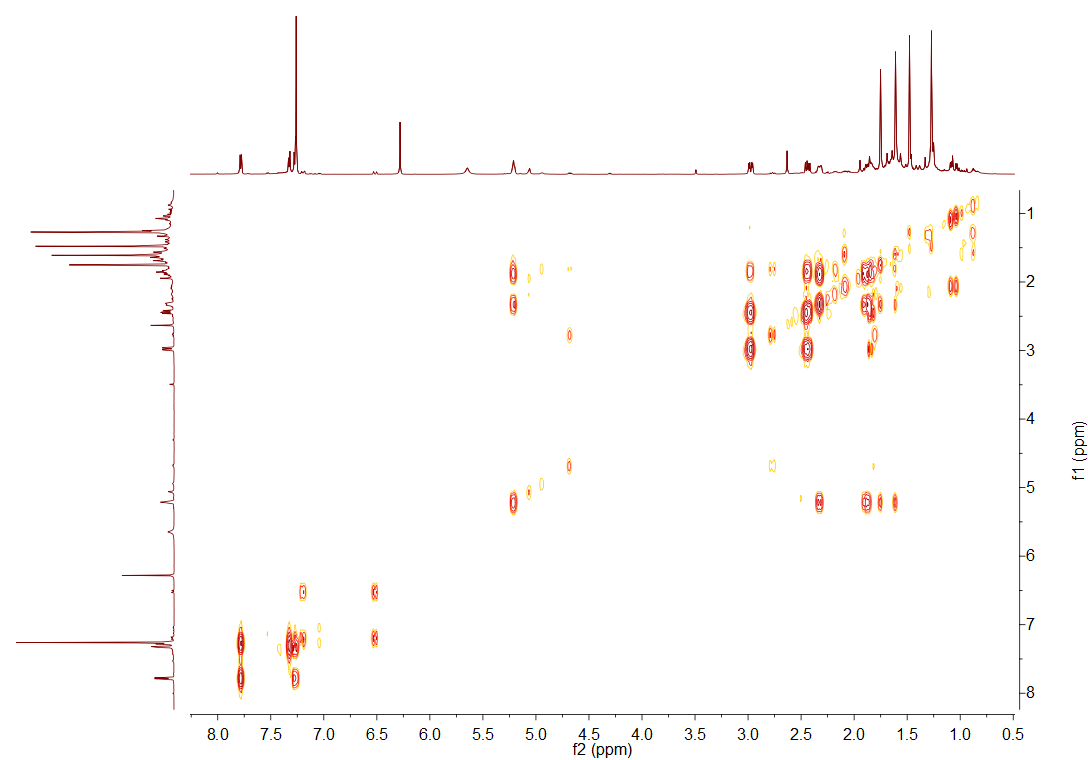


S49. ^1^H-^1^H COSY spectrum of compound **5** (CDCl_3_)


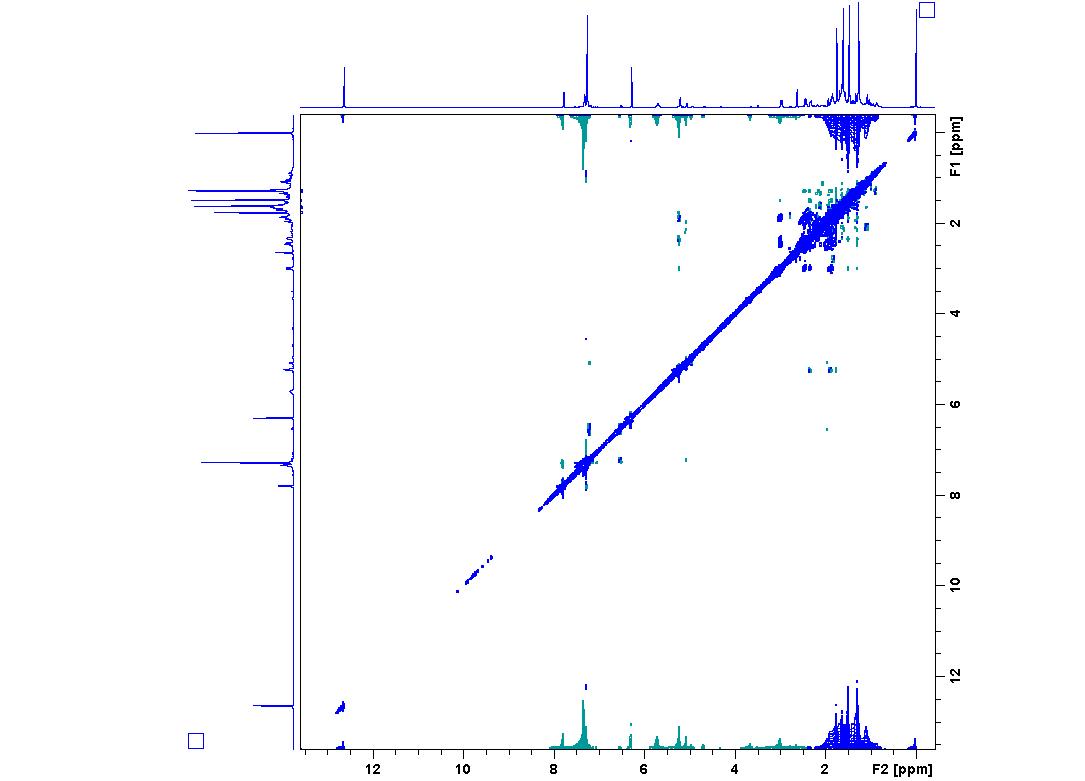


S50. ROESY spectrum of compound **5** (CDCl_3_)


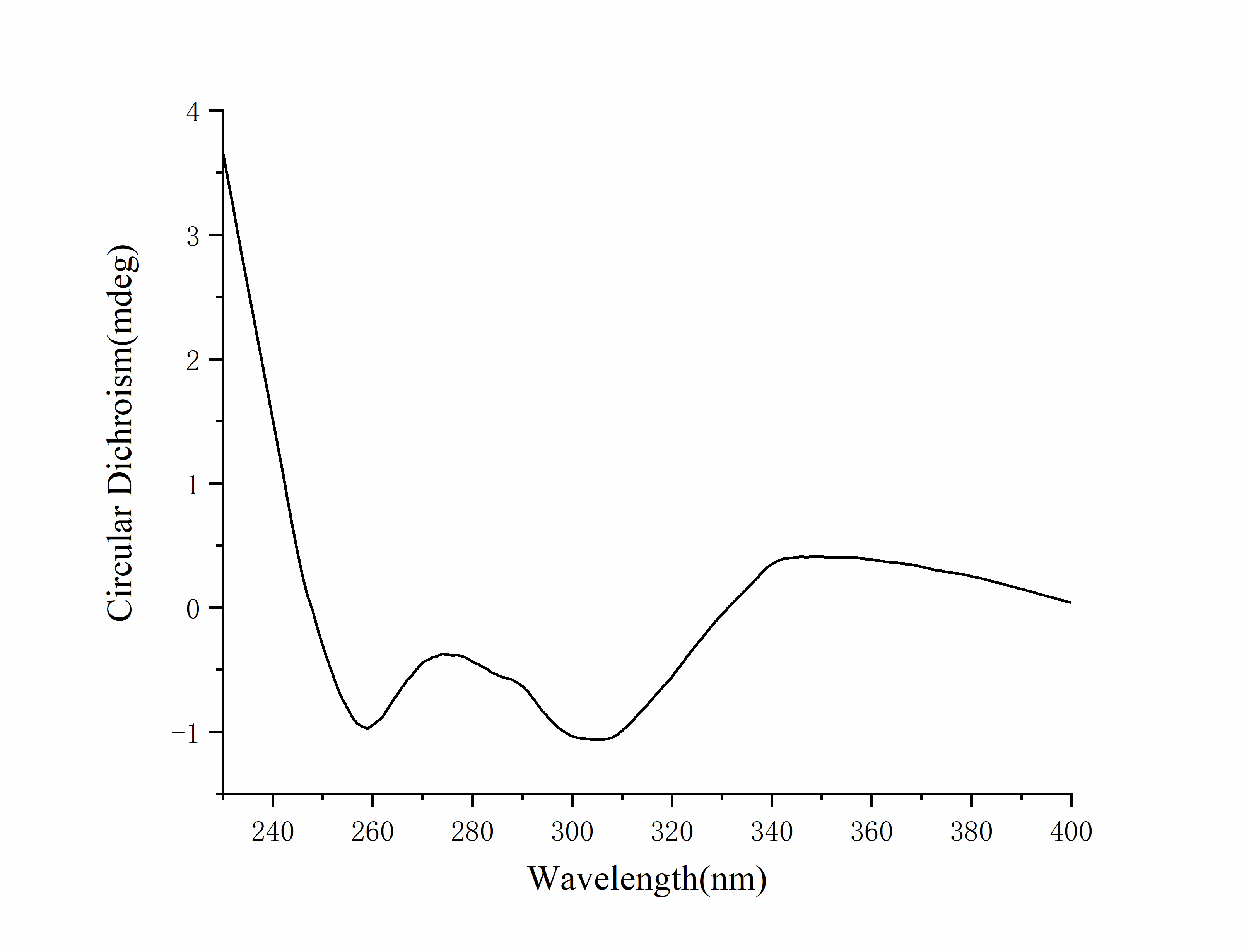


S51. CD spectrum of **5**


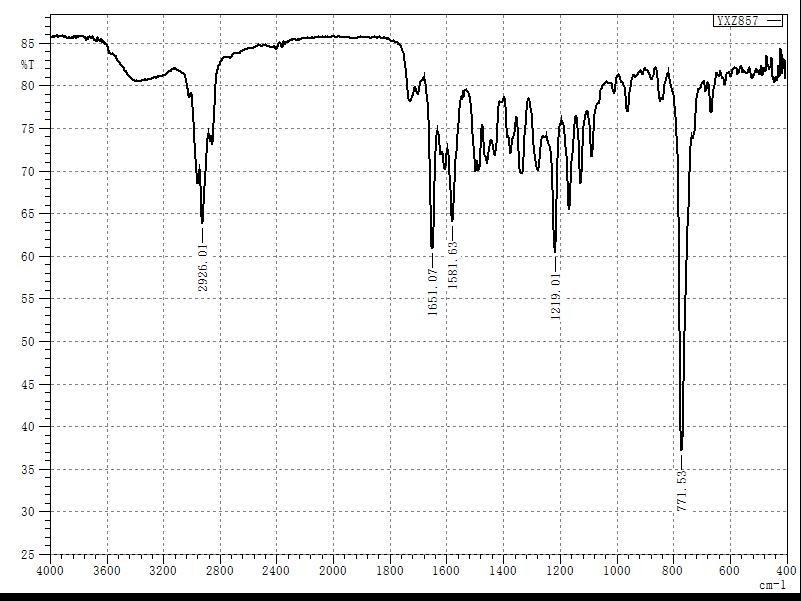


S52. IR spectrum of **5**


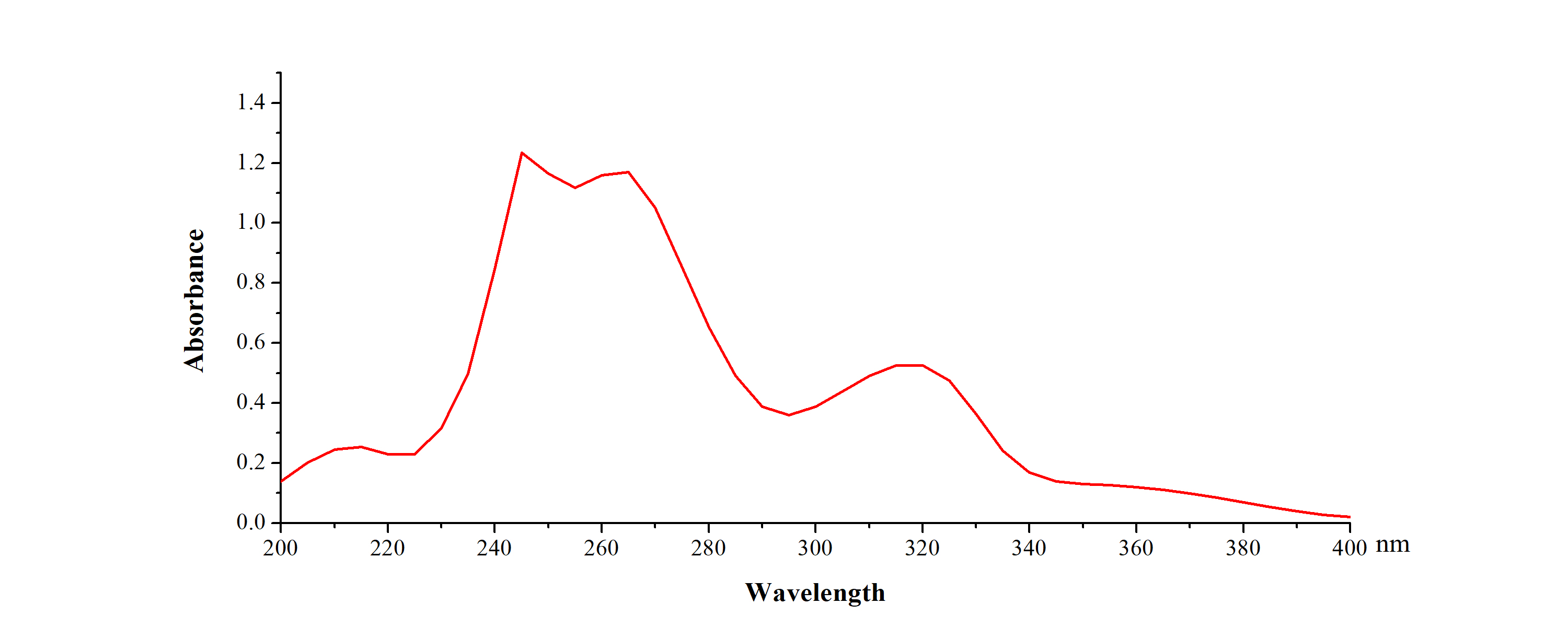


S53. UV spectrum of **5**


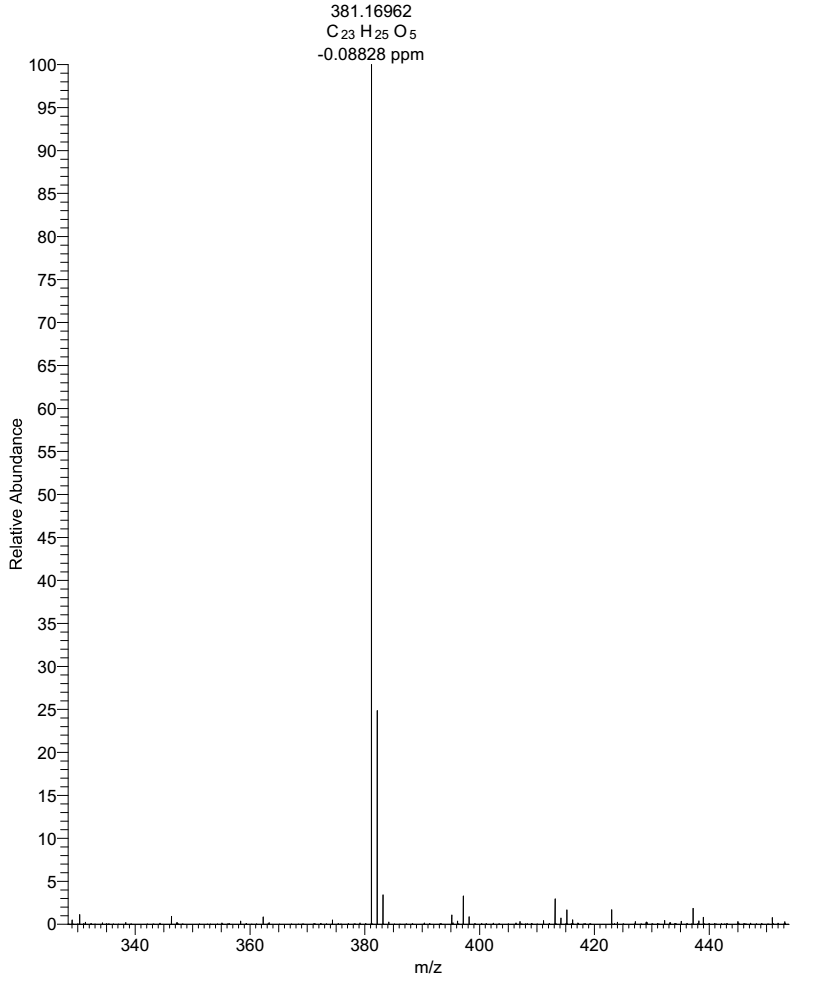


S54. HRESIMS data of **5**


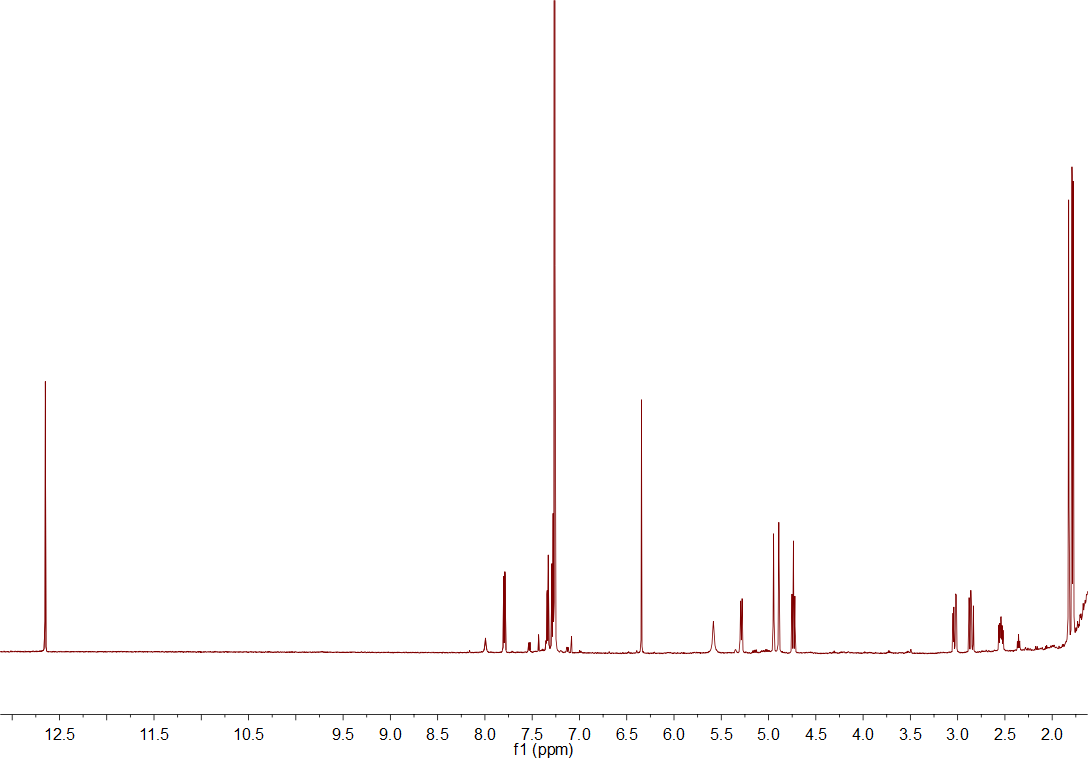


S55. ^1^H NMR spectrum of compound **6** (CDCl_3_, 600 MHz)


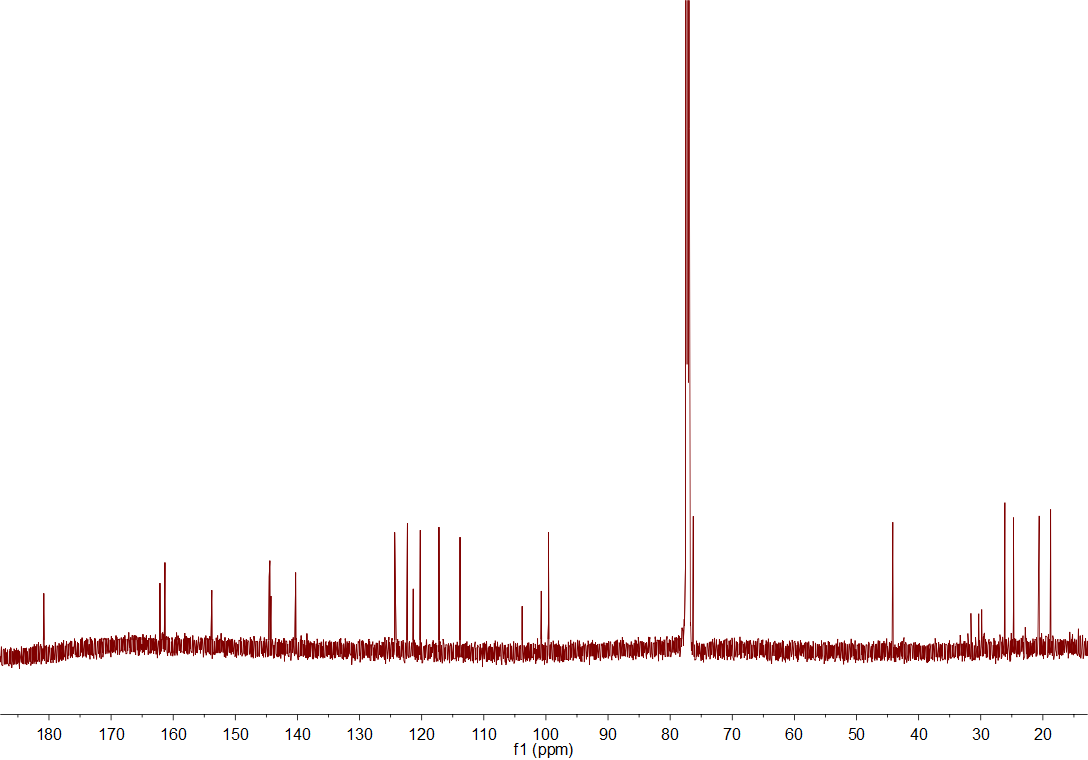


S56. ^13^C NMR spectrum of compound **6** (CDCl_3_, 150 MHz)


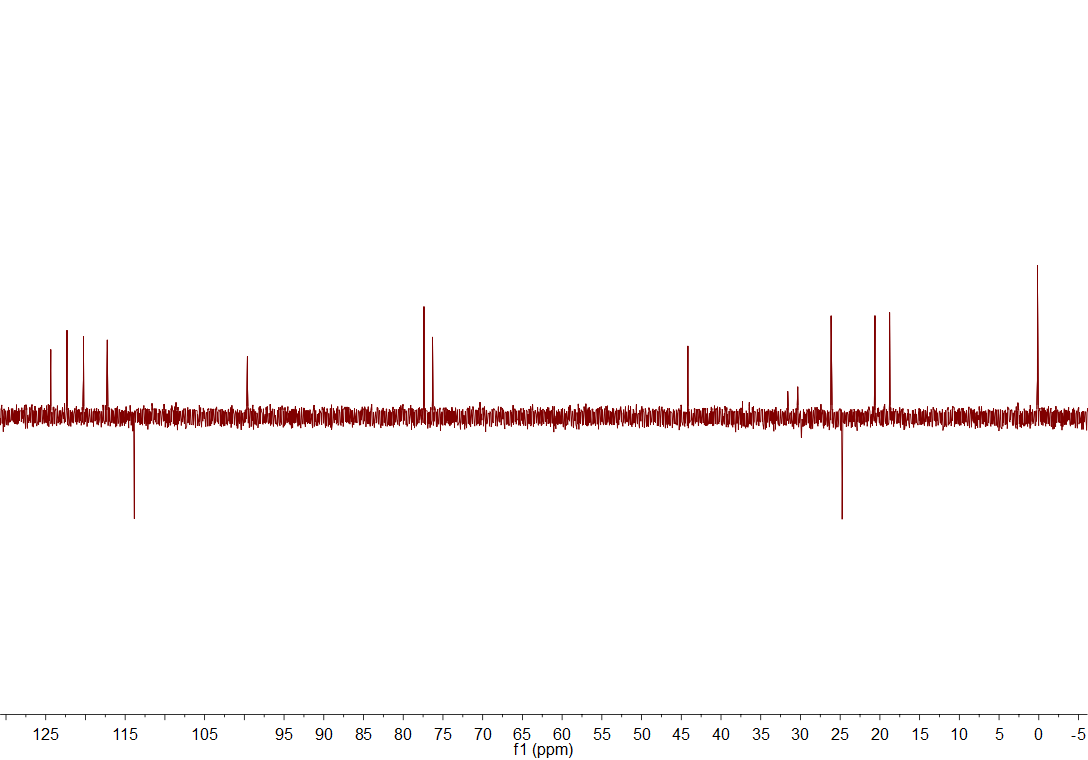


S57. DEPT 135° spectrum of compound **6** (CDCl_3_, 150 MHz)


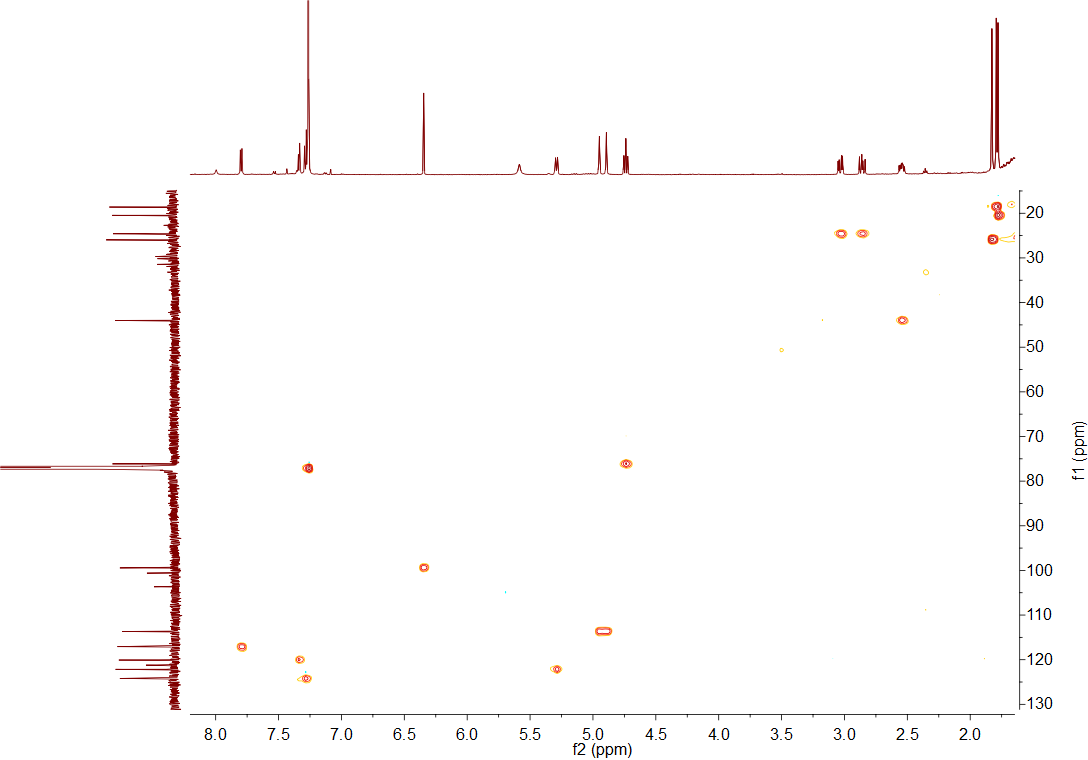


S58. HSQC spectrum of compound **6** (CDCl_3_)


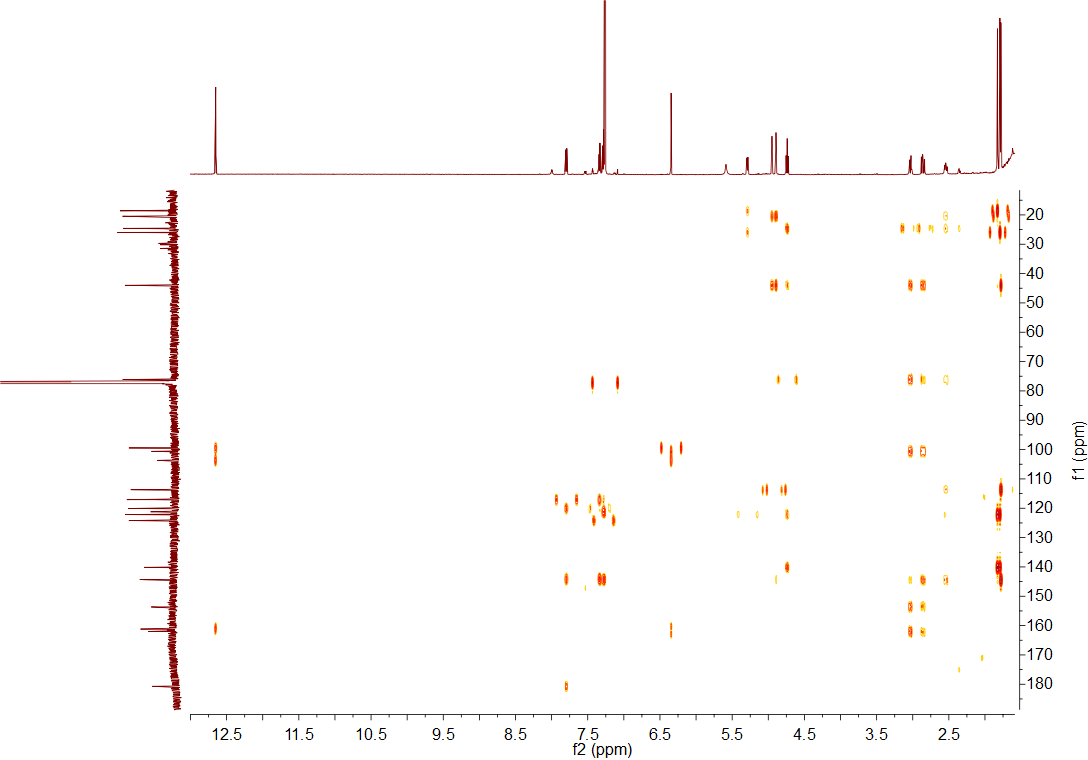


S59. HMBC spectrum of compound **6** (CDCl_3_)


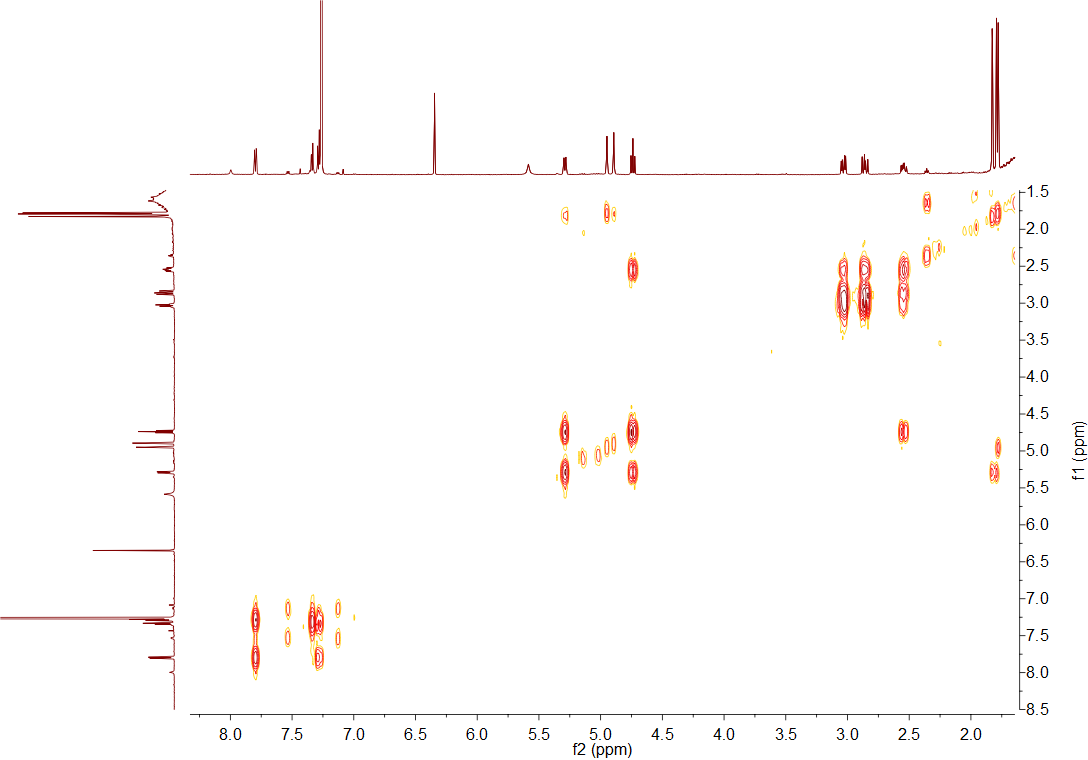


S60. ^1^H-^1^H COSY spectrum of compound **6** (CDCl_3_)


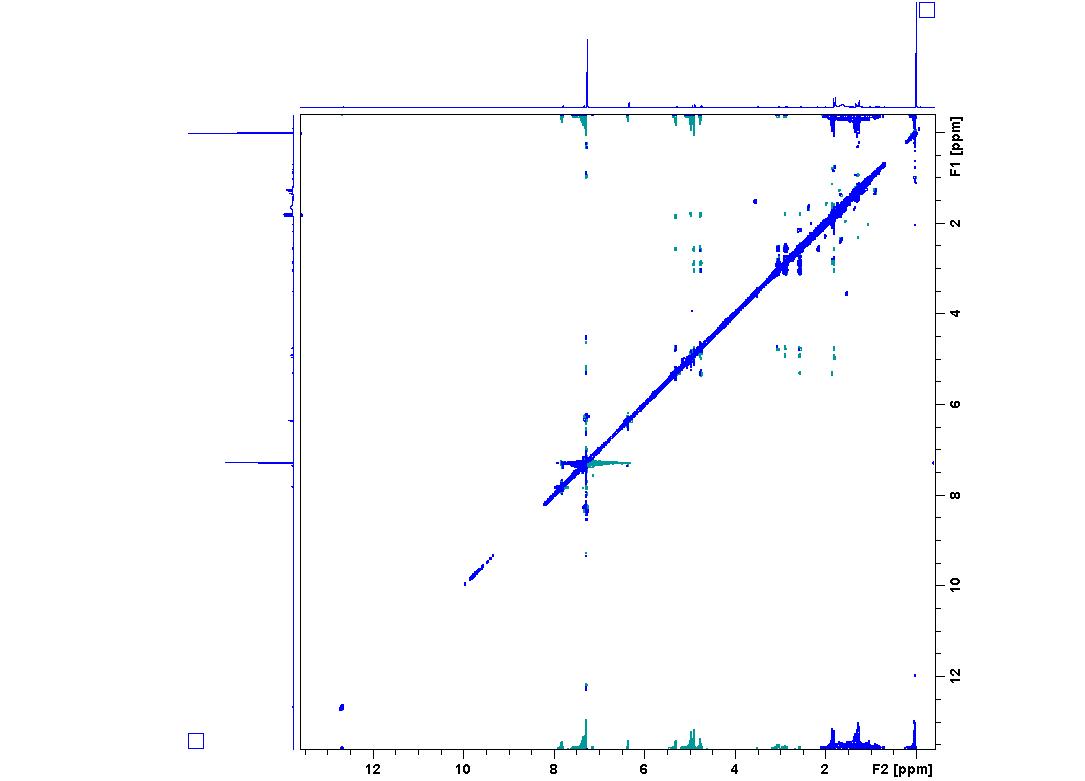


S61. ROESY spectrum of compound **6** (CDCl_3_)


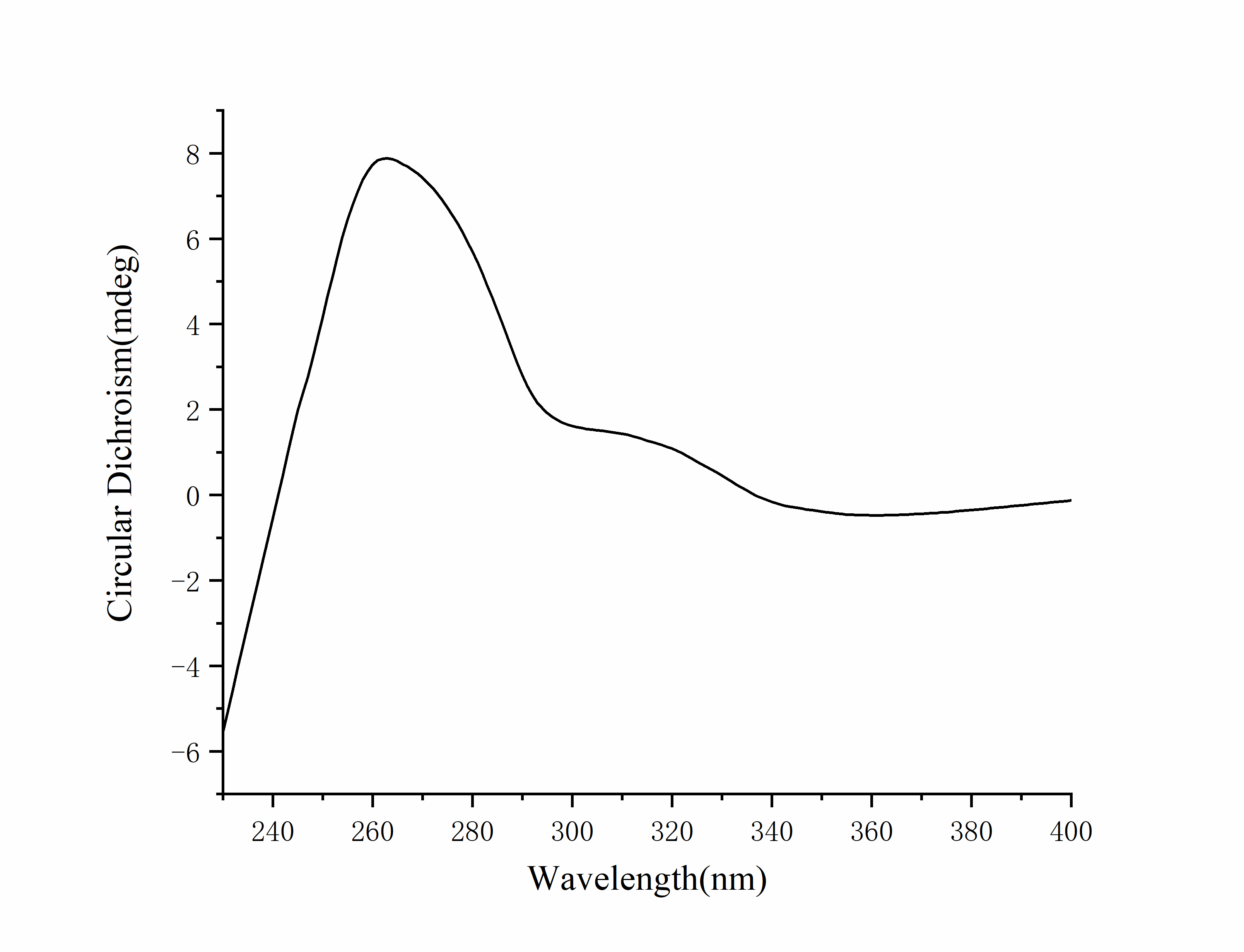


S62. CD spectrum of **6**

**
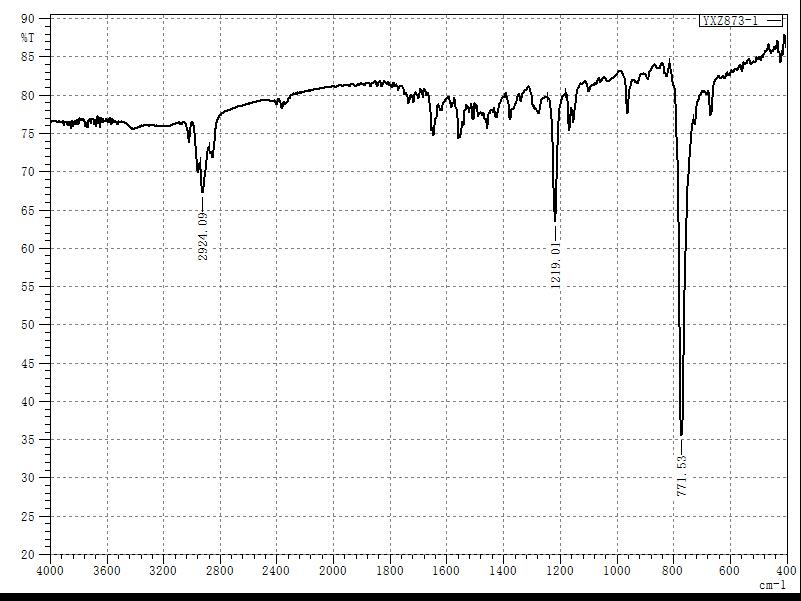
**

S63. IR spectrum of **6**

**
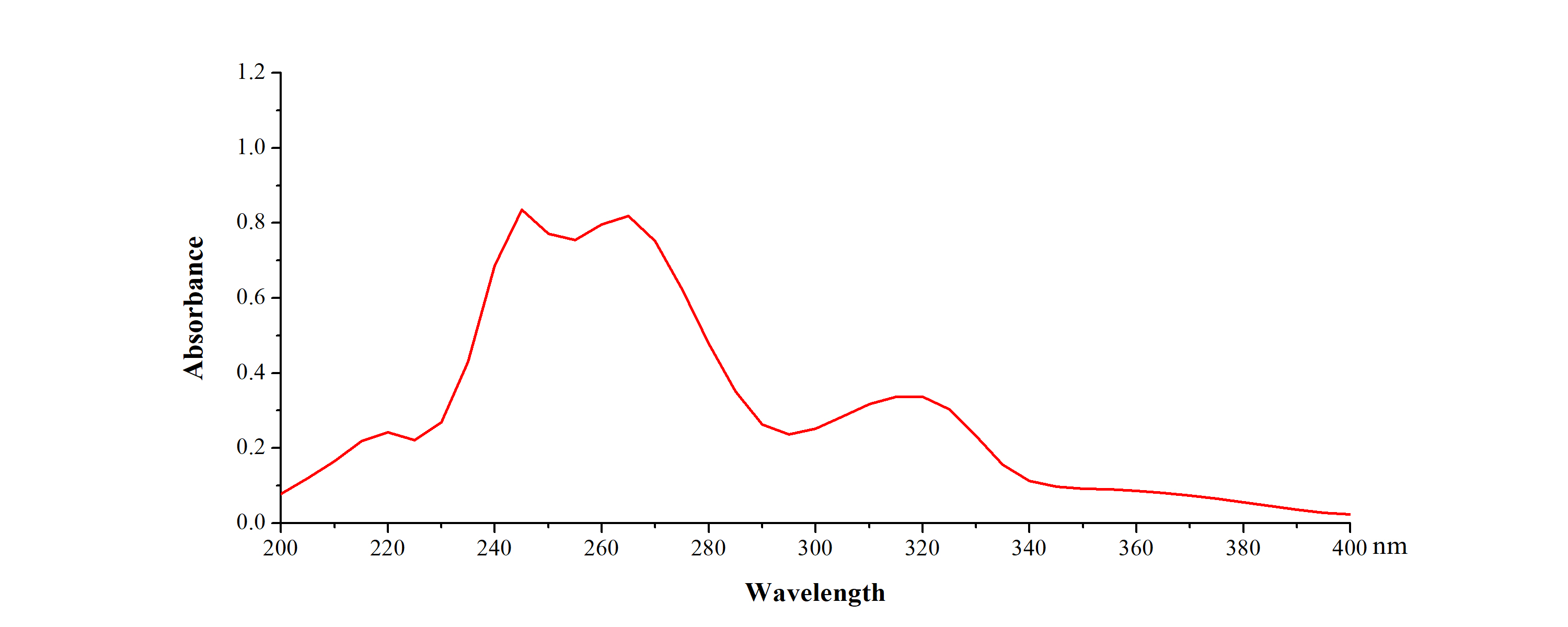
**

S64. UV spectrum of **6**


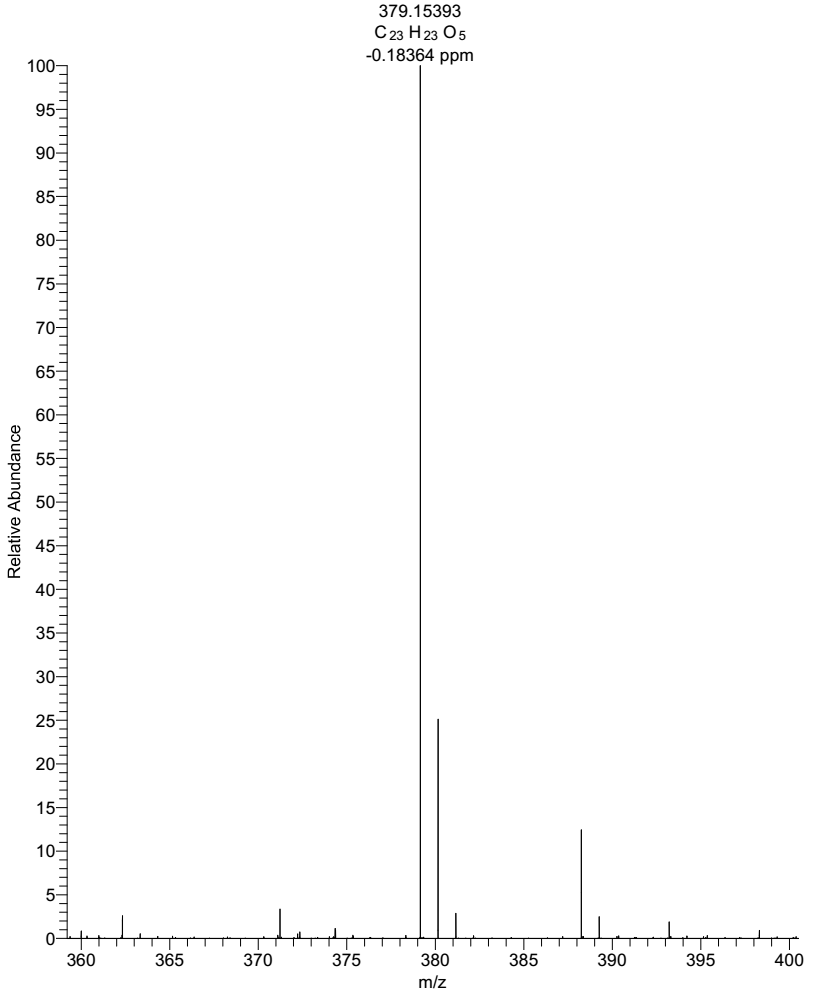


S65. HREIMS spectrum of **6**

S66. NMR calculation section

NMR calculation Data of **1**


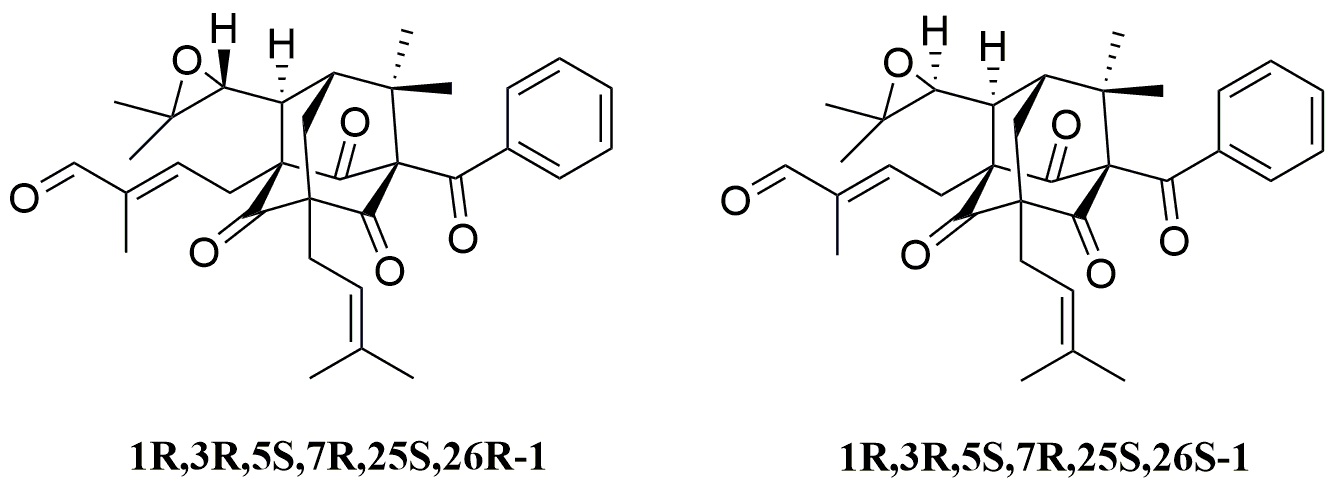


Table S1. Experimental and Calculated ^13^C NMR Data for **1** (δ in ppm).

| no | 1R,3R,5S,7R,25S,26R-**1** | | | 1R,3R,5S,7R,25S,26S-**1** | | |
| --- | --- | --- | --- | --- | --- | --- |
|  | *δ*_C_, exptl^a^ | *δ*_C_, (adj_calcd)^b^ | *δ*_C_, Δ*δ*^c^ | *δ*_C_, exptl^a^ | *δ*_C_, (adj_calcd)^b^ | *δ*_C_, Δ*δ*^c^ |
| 1 | 81.9 | 88.2 | 6.3 | 81.9 | 88.1 | 6.2 |
| 2 | 200.8 | 213.5 | 12.7 | 200.8 | 213.5 | 12.7 |
| 3 | 70.6 | 79.0 | 8.4 | 70.6 | 80.6 | 10.0 |
| 4 | 202.6 | 216.3 | 13.7 | 202.6 | 215.6 | 13.0 |
| 5 | 69.2 | 76.9 | 7.7 | 69.2 | 76.4 | 7.2 |
| 6 | 40.3 | 44.7 | 4.4 | 40.3 | 45.1 | 4.8 |
| 7 | 44.5 | 50.0 | 5.5 | 44.5 | 51.3 | 6.8 |
| 8 | 56.1 | 66.4 | 10.3 | 56.1 | 65.9 | 9.8 |
| 9 | 201.3 | 215.2 | 13.9 | 201.3 | 214.9 | 13.6 |
| 10 | 192.3 | 203.1 | 10.8 | 192.3 | 203.1 | 10.8 |
| 11 | 134.5 | 138.9 | 4.4 | 134.5 | 138.9 | 4.4 |
| 12 | 129.1 | 137.0 | 7.9 | 129.1 | 137.3 | 8.2 |
| 12' | 129.1 | 137.8 | 8.7 | 129.1 | 137.6 | 8.5 |
| 13 | 128.2 | 134.3 | 6.1 | 128.2 | 134.2 | 6.0 |
| 13' | 128.2 | 133.5 | 5.3 | 128.2 | 133.7 | 5.5 |
| 14 | 133.0 | 140.6 | 7.6 | 133.0 | 140.6 | 7.6 |
| 15 | 27.5 | 33.5 | 6.0 | 27.5 | 33.7 | 6.2 |
| 16 | 147.9 | 159.5 | 11.6 | 147.9 | 160.3 | 12.4 |
| 17 | 141.6 | 150.3 | 8.7 | 141.6 | 150.5 | 8.9 |
| 18 | 194.7 | 200.8 | 6.1 | 194.7 | 201.0 | 6.3 |
| 19 | 9.7 | 11.7 | 2.0 | 9.7 | 12.4 | 2.7 |
| 20 | 27.7 | 32.1 | 4.4 | 27.7 | 32.1 | 4.4 |
| 21 | 117.7 | 127.3 | 9.6 | 117.7 | 127.4 | 9.7 |
| 22 | 136.1 | 147.2 | 11.1 | 136.1 | 147.2 | 11.1 |
| 23 | 26.2 | 29.1 | 2.9 | 26.2 | 29.0 | 2.8 |
| 24 | 18.3 | 19.7 | 1.4 | 18.3 | 19.7 | 1.4 |
| 25 | 51.8 | 60.1 | 8.3 | 51.8 | 62.7 | 10.9 |
| 26 | 60.5 | 64.5 | 4.0 | 60.5 | 65.8 | 5.3 |
| 27 | 61.2 | 66.3 | 5.1 | 61.2 | 62.4 | 1.2 |
| 28 | 19.8 | 21.3 | 1.5 | 19.8 | 26.6 | 6.8 |
| 29 | 24.5 | 26.1 | 1.6 | 24.5 | 20.6 | 3.9 |
| 30 | 23.6 | 25.1 | 1.5 | 23.6 | 24.7 | 1.1 |
| 31 | 22.7 | 24.0 | 1.3 | 22.7 | 24.2 | 1.5 |

^a^Recorded in CD_3_Cl_3_ at 150 MHz. ^b^Calculated in CD_3_Cl_3_. ^c^Δ*δ* = |*δ*adj_calcd-*δ*_exptl_|

**Table S2. Conformers and Bolzmann distributions of the optimized 1R,3R,5S,7R,25S,26R-1**

| species | *G* | *ΔG* | *ΔE(kcal/mol)* | *p%* |
| --- | --- | --- | --- | --- |
| 1 | -1731.095983 | 0 | 0 | 44.81% |
| 2 | -1731.095157 | 0.000826 | 0.518322847 | 18.67% |
| 3 | -1731.095006 | 0.000977 | 0.613076781 | 15.91% |
| 4 | -1731.094695 | 0.001288 | 0.808232236 | 11.44% |
| 5 | -1731.093657 | 0.002326 | 1.459587097 | 3.81% |
| 6 | -1731.093479 | 0.002504 | 1.571283788 | 3.15% |
| 7 | -1731.092073 | 0.00391 | 2.453562145 | 0.71% |
| 8 | -1731.091616 | 0.004367 | 2.740333987 | 0.44% |
| 9 | -1731.091588 | 0.004395 | 2.757904252 | 0.43% |
| 10 | -1731.091153 | 0.00483 | 3.030870885 | 0.27% |
| 11 | -1731.090912 | 0.005071 | 3.182100675 | 0.21% |
| 12 | -1731.090358 | 0.005625 | 3.529740938 | 0.12% |
| 13 | -1731.089025 | 0.006958 | 4.366211101 | 0.03% |

Table S3. The coordinate for the lowest-energy conformer NMR calculations of the optimized 1R,3R,5S,7R,25S,26R-**1**

| 1R,3R,5S,7R,25S,26R-**1** Conf. 1 | | Standard Orientation (Ångstroms) | | |
| --- | --- | --- | --- | --- |
| I | atom | X | Y | Z |
| 1 | C | -0.135918 | 1.26105 | -0.127333 |
| 2 | C | -0.768434 | 0.39993 | -1.23146 |
| 3 | C | -1.366216 | -0.940735 | -0.77213 |
| 4 | C | -0.279049 | -1.706605 | -0.006256 |
| 5 | C | 0.388458 | -0.941669 | 1.149015 |
| 6 | C | 0.864626 | 0.444227 | 0.701537 |
| 7 | C | -1.282457 | 1.563083 | 0.925919 |
| 8 | C | -1.856624 | 0.219523 | 1.447049 |
| 9 | C | -0.789516 | -0.632881 | 2.214542 |
| 10 | C | 1.583359 | -1.766761 | 1.716433 |
| 11 | C | 2.78696 | -2.041699 | 0.8645 |
| 12 | O | 1.540181 | -2.190212 | 2.85929 |
| 13 | C | 2.931711 | -1.640739 | -0.472008 |
| 14 | C | 4.093179 | -1.93723 | -1.181107 |
| 15 | C | 5.129351 | -2.64116 | -0.564894 |
| 16 | C | 4.996957 | -3.04949 | 0.76439 |
| 17 | C | 3.836398 | -2.752244 | 1.471235 |
| 18 | C | -0.260517 | 0.13817 | 3.443942 |
| 19 | C | -1.431835 | -1.941507 | 2.729444 |
| 20 | C | -2.467383 | -0.564385 | 0.277164 |
| 21 | C | 0.501417 | 2.561478 | -0.670951 |
| 22 | C | 1.621015 | 2.358638 | -1.654275 |
| 23 | C | 2.917394 | 2.703273 | -1.521835 |
| 24 | C | 3.790444 | 2.402832 | -2.680188 |
| 25 | C | 3.584029 | 3.348321 | -0.339622 |
| 26 | O | -0.858921 | 0.789884 | -2.37988 |
| 27 | C | -1.928187 | -1.738583 | -1.972198 |
| 28 | C | -2.73928 | -2.955211 | -1.606458 |
| 29 | C | -4.046921 | -3.169372 | -1.828382 |
| 30 | C | -4.684608 | -4.479661 | -1.428168 |
| 31 | C | -4.994852 | -2.193615 | -2.482802 |
| 32 | O | 0.005999 | -2.86105 | -0.253015 |
| 33 | O | 1.904366 | 0.924322 | 1.104231 |
| 34 | C | -2.365254 | 2.457685 | 0.335975 |
| 35 | C | -2.664024 | 3.847754 | 0.755192 |
| 36 | O | -3.483672 | 2.737844 | 1.192851 |
| 37 | C | -3.347672 | 4.765885 | -0.23955 |
| 38 | C | -1.899728 | 4.557974 | 1.853383 |
| 39 | O | 4.981453 | 2.648784 | -2.727799 |
| 40 | H | -0.812463 | 2.093106 | 1.757379 |
| 41 | H | -2.652507 | 0.464825 | 2.159514 |
| 42 | H | 2.14579 | -1.093147 | -0.980593 |
| 43 | H | 4.190368 | -1.616018 | -2.214085 |
| 44 | H | 6.035958 | -2.868758 | -1.118968 |
| 45 | H | 5.799786 | -3.597799 | 1.249457 |
| 46 | H | 3.717674 | -3.060529 | 2.504079 |
| 47 | H | 0.310203 | 1.03498 | 3.1904 |
| 48 | H | -1.111158 | 0.443487 | 4.065437 |
| 49 | H | 0.38904 | -0.502739 | 4.040186 |
| 50 | H | -0.739279 | -2.466247 | 3.387113 |
| 51 | H | -2.337634 | -1.697761 | 3.298021 |
| 52 | H | -1.714094 | -2.634067 | 1.932157 |
| 53 | H | -3.242549 | 0.025002 | -0.22486 |
| 54 | H | -2.955972 | -1.48056 | 0.614083 |
| 55 | H | -0.292567 | 3.125016 | -1.175863 |
| 56 | H | 0.835998 | 3.152536 | 0.184624 |
| 57 | H | 1.319016 | 1.898994 | -2.594287 |
| 58 | H | 3.274183 | 1.919412 | -3.540786 |
| 59 | H | 4.131665 | 4.24229 | -0.657624 |
| 60 | H | 2.878075 | 3.618777 | 0.446709 |
| 61 | H | 4.323452 | 2.66475 | 0.092193 |
| 62 | H | -2.503849 | -1.038877 | -2.582269 |
| 63 | H | -1.075667 | -2.049656 | -2.589319 |
| 64 | H | -2.170163 | -3.752757 | -1.133405 |
| 65 | H | -3.967081 | -5.154031 | -0.950858 |
| 66 | H | -5.519315 | -4.318406 | -0.731409 |
| 67 | H | -5.106997 | -4.995559 | -2.302027 |
| 68 | H | -4.522394 | -1.252732 | -2.774302 |
| 69 | H | -5.441729 | -2.636268 | -3.383751 |
| 70 | H | -5.83064 | -1.956189 | -1.809945 |
| 71 | H | -2.661036 | 2.201213 | -0.684248 |
| 72 | H | -2.614045 | 5.394948 | -0.757496 |
| 73 | H | -3.903357 | 4.191448 | -0.986307 |
| 74 | H | -4.052822 | 5.427627 | 0.276782 |
| 75 | H | -2.566317 | 5.257833 | 2.370451 |
| 76 | H | -1.507022 | 3.864428 | 2.600303 |
| 77 | H | -1.066743 | 5.137199 | 1.43749 |

**Table S4. Conformers and Bolzmann distributions of the optimized 1R,3R,5S,7R,25S,26S-1**

| species | *G* | *ΔG* | *ΔE(kcal/mol)* | *p%* |
| --- | --- | --- | --- | --- |
| 1 | -1731.093963 | 0 | 0 | 24.54% |
| 2 | -1731.093841 | 0.000122 | 0.076556159 | 21.56% |
| 3 | -1731.093613 | 0.00035 | 0.219628325 | 16.93% |
| 4 | -1731.093535 | 0.000428 | 0.268574066 | 15.59% |
| 5 | -1731.093199 | 0.000764 | 0.479417258 | 10.92% |
| 6 | -1731.092467 | 0.001496 | 0.938754212 | 5.03% |
| 7 | -1731.09206 | 0.001903 | 1.194150579 | 3.27% |
| 8 | -1731.091305 | 0.002658 | 1.667920251 | 1.47% |
| 9 | -1731.090114 | 0.003849 | 2.415284065 | 0.42% |
| 10 | -1731.089746 | 0.004217 | 2.646207561 | 0.28% |

**Table S5. The coordinate for the lowest-energy conformer NMR calculations of the optimized 1R,3R,5S,7R,25S,26S-1**

| 1R,3R,5S,7R,25S,26S-**1** Conf. 1 | | Standard Orientation (Ångstroms) | | |
| --- | --- | --- | --- | --- |
| I | atom | X | Y | Z |
| 1 | C | 0.288199 | 1.21763 | -0.435499 |
| 2 | C | 0.575336 | 0.619811 | 0.950456 |
| 3 | C | 0.786928 | -0.906988 | 0.990658 |
| 4 | C | -0.414983 | -1.57856 | 0.3199 |
| 5 | C | -0.760215 | -1.070371 | -1.090644 |
| 6 | C | -0.846833 | 0.459146 | -1.133043 |
| 7 | C | 1.547749 | 0.866573 | -1.342246 |
| 8 | C | 1.736959 | -0.672733 | -1.366564 |
| 9 | C | 0.519778 | -1.421987 | -2.011506 |
| 10 | C | -2.104912 | -1.697794 | -1.566848 |
| 11 | C | -3.382638 | -1.378769 | -0.847792 |
| 12 | O | -2.119786 | -2.450538 | -2.526222 |
| 13 | C | -3.479745 | -0.569996 | 0.294827 |
| 14 | C | -4.713871 | -0.332926 | 0.89538 |
| 15 | C | -5.872738 | -0.899723 | 0.361417 |
| 16 | C | -5.790427 | -1.70682 | -0.77563 |
| 17 | C | -4.556678 | -1.943938 | -1.373125 |
| 18 | C | 0.314927 | -0.963773 | -3.47218 |
| 19 | C | 0.789262 | -2.944134 | -2.03639 |
| 20 | C | 2.015845 | -1.176595 | 0.058332 |
| 21 | C | 0.021222 | 2.740989 | -0.379489 |
| 22 | C | -1.239263 | 3.128835 | 0.342249 |
| 23 | C | -1.356151 | 3.861238 | 1.468069 |
| 24 | C | -2.737001 | 4.107566 | 1.942116 |
| 25 | C | -0.24776 | 4.436429 | 2.305038 |
| 26 | O | 0.722333 | 1.315302 | 1.934502 |
| 27 | C | 1.016427 | -1.39735 | 2.439729 |
| 28 | C | 1.491832 | -2.822195 | 2.563339 |
| 29 | C | 2.672005 | -3.257542 | 3.035269 |
| 30 | C | 2.960265 | -4.738184 | 3.126203 |
| 31 | C | 3.795885 | -2.376771 | 3.525793 |
| 32 | O | -1.027536 | -2.493453 | 0.832768 |
| 33 | O | -1.687526 | 1.028149 | -1.801144 |
| 34 | C | 2.791516 | 1.613249 | -0.883119 |
| 35 | C | 3.961351 | 1.910413 | -1.744336 |
| 36 | O | 2.992919 | 2.931681 | -1.419914 |
| 37 | C | 4.021733 | 1.517793 | -3.207339 |
| 38 | C | 5.312363 | 2.107767 | -1.083522 |
| 39 | O | -3.023562 | 4.745105 | 2.938974 |
| 40 | H | 1.313012 | 1.23159 | -2.344843 |
| 41 | H | 2.610739 | -0.895201 | -1.992149 |
| 42 | H | -2.599864 | -0.116529 | 0.738262 |
| 43 | H | -4.769697 | 0.293591 | 1.780966 |
| 44 | H | -6.834892 | -0.712837 | 0.830625 |
| 45 | H | -6.68861 | -2.150798 | -1.195857 |
| 46 | H | -4.475142 | -2.568951 | -2.255525 |
| 47 | H | 0.018185 | 0.083439 | -3.568044 |
| 48 | H | 1.252882 | -1.101992 | -4.023923 |
| 49 | H | -0.456771 | -1.566679 | -3.951013 |
| 50 | H | 0.01732 | -3.451169 | -2.614895 |
| 51 | H | 1.760355 | -3.131662 | -2.511168 |
| 52 | H | 0.80831 | -3.402475 | -1.04427 |
| 53 | H | 2.892437 | -0.678015 | 0.487059 |
| 54 | H | 2.238516 | -2.245267 | 0.074001 |
| 55 | H | 0.891591 | 3.228206 | 0.060684 |
| 56 | H | -0.055572 | 3.088608 | -1.417104 |
| 57 | H | -2.160537 | 2.793921 | -0.133109 |
| 58 | H | -3.533881 | 3.656375 | 1.307913 |
| 59 | H | -0.510489 | 5.448535 | 2.62792 |
| 60 | H | -0.108748 | 3.83651 | 3.211922 |
| 61 | H | 0.706185 | 4.464464 | 1.77496 |
| 62 | H | 1.709684 | -0.696719 | 2.909978 |
| 63 | H | 0.065756 | -1.289918 | 2.977111 |
| 64 | H | 0.764851 | -3.570294 | 2.253287 |
| 65 | H | 2.122778 | -5.340946 | 2.76181 |
| 66 | H | 3.853522 | -5.005668 | 2.543946 |
| 67 | H | 3.167547 | -5.036126 | 4.16364 |
| 68 | H | 3.575231 | -1.308916 | 3.459873 |
| 69 | H | 4.03397 | -2.604201 | 4.574016 |
| 70 | H | 4.714979 | -2.567492 | 2.953984 |
| 71 | H | 2.98926 | 1.564392 | 0.189643 |
| 72 | H | 4.560218 | 0.57098 | -3.333658 |
| 73 | H | 3.029767 | 1.418626 | -3.652986 |
| 74 | H | 4.564945 | 2.285323 | -3.770887 |
| 75 | H | 5.899491 | 1.18198 | -1.106656 |
| 76 | H | 5.882917 | 2.880647 | -1.611802 |
| 77 | H | 5.196815 | 2.422982 | -0.042457 |


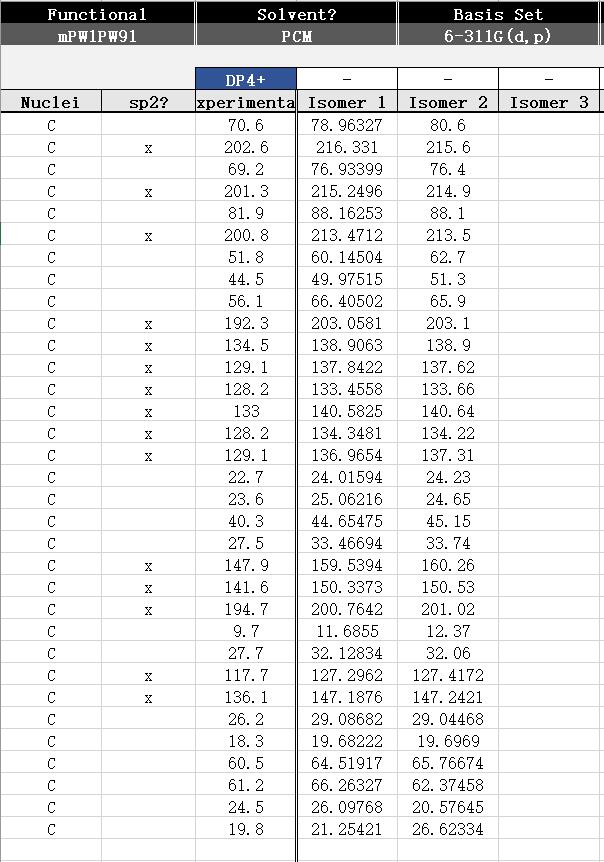


Figure S1. DP4+ analysis of compound **1** with isomer 1 (1R,3R,5S,7R,25S,26R) and isomer 2 (1R,3R,5S,7R,25S,26S).

NMR calculation Data of **2**


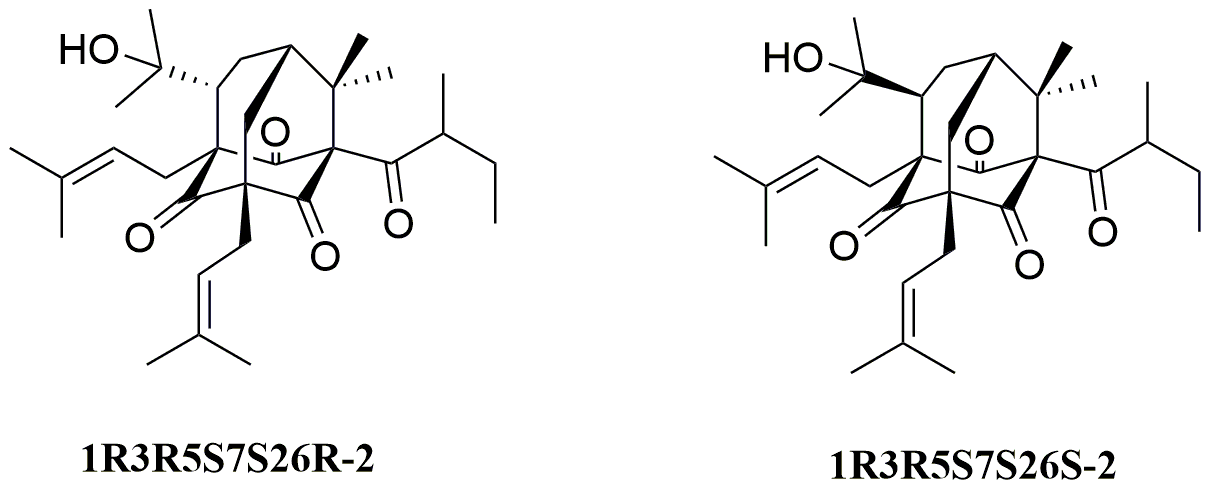


Table S1. Experimental and Calculated ^13^C NMR Data for **2** (δ in ppm).

| no. | 1R3R5S7S26S-**2** | | | 1R3R5S7S26R-**2** | | |
| --- | --- | --- | --- | --- | --- | --- |
|  | *δ*_C_, exptl^a^ | *δ*_C_, (adj_calcd)^b^ | *δ*_C_, Δ*δ*^c^ | *δ*_C_, exptl^a^ | *δ*_C_, (adj_calcd)^b^ | *δ*_C_, Δ*δ*^c^ |
| 1 | 71.1 | 78.5 | 7.4 | 71.1 | 78.5 | 7.4 |
| 2 | 208.5 | 225.6 | 17.1 | 208.5 | 222.0 | 13.5 |
| 3 | 68.1 | 76.7 | 8.6 | 68.1 | 75.2 | 7.1 |
| 4 | 205.5 | 219.4 | 13.9 | 205.5 | 219.3 | 13.8 |
| 5 | 87.6 | 94.6 | 7.0 | 87.6 | 94.7 | 7.1 |
| 6 | 206.3 | 220.2 | 13.9 | 206.3 | 220.6 | 14.3 |
| 7 | 49.7 | 54.3 | 4.6 | 49.7 | 56.2 | 6.5 |
| 8 | 208.7 | 223.5 | 14.8 | 208.7 | 224.0 | 15.3 |
| 9 | 50.3 | 49.5 | 0.8 | 50.3 | 49.2 | 1.1 |
| 10 | 34.3 | 42.0 | 7.7 | 34.3 | 39.7 | 5.4 |
| 11 | 119.8 | 127.3 | 7.5 | 119.8 | 128.8 | 9.0 |
| 12 | 136.5 | 147.92 | 11.4 | 136.5 | 147.01 | 10.5 |
| 13 | 18.3 | 28.51 | 10.2 | 18.3 | 28.57 | 10.3 |
| 14 | 26.3 | 20.26 | 6.0 | 26.3 | 20.28 | 6.0 |
| 15 | 29 | 33.02 | 4.0 | 29 | 32.92 | 3.9 |
| 16 | 119.3 | 128.04 | 8.7 | 119.3 | 128.48 | 9.2 |
| 17 | 135.2 | 146.97 | 11.8 | 135.2 | 146.42 | 11.2 |
| 18 | 18.2 | 28.81 | 10.6 | 18.2 | 28.85 | 10.6 |
| 19 | 26.2 | 19.73 | 6.5 | 26.2 | 19.47 | 6.7 |
| 20 | 20.9 | 24.32 | 3.4 | 20.9 | 24.43 | 3.5 |
| 21 | 20.4 | 22.90 | 2.5 | 20.4 | 22.89 | 2.5 |
| 22 | 47.7 | 58.97 | 11.3 | 47.7 | 56.55 | 8.8 |
| 23 | 43.6 | 50.23 | 6.6 | 43.6 | 48.54 | 4.9 |
| 24 | 38.5 | 44.15 | 5.7 | 38.5 | 42.72 | 4.2 |
| 25 | 29.7 | 34.49 | 4.8 | 29.7 | 33.71 | 4.0 |
| 26 | 76.1 | 80.61 | 4.5 | 76.1 | 81.83 | 5.7 |
| 27 | 26.8 | 30.95 | 4.2 | 26.8 | 23.84 | 3.0 |
| 28 | 33.2 | 34.40 | 1.2 | 33.2 | 35.83 | 2.6 |
| 29 | 24.9 | 25.66 | 0.8 | 24.9 | 25.79 | 0.9 |
| 30 | 22.8 | 23.68 | 0.9 | 22.8 | 23.96 | 1.2 |

^a^Recorded in CD_3_Cl_3_ at 150 MHz. ^b^Calculated in CD_3_Cl_3_. ^c^Δ*δ* = |*δ*adj_calcd-*δ*_exptl_|

**Table S2. Conformers and Bolzmann distributions of the optimized 1R3R5S7S26S-2**

| species | *G* | *ΔG* | *ΔE(kcal/mol)* | *p%* |
| --- | --- | --- | --- | --- |
| 1 | -1545.136054 | 0 | 0 | 28.04% |
| 2 | -1545.135999 | 5.5E-05 | 0.034513022 | 26.46% |
| 3 | -1545.135909 | 0.000145 | 0.090988877 | 24.05% |
| 4 | -1545.134427 | 0.001627 | 1.020957957 | 5.00% |
| 5 | -1545.134347 | 0.001707 | 1.071158717 | 4.59% |
| 6 | -1545.13403 | 0.002024 | 1.270079228 | 3.28% |
| 7 | -1545.133778 | 0.002276 | 1.428211622 | 2.51% |
| 8 | -1545.13343 | 0.002624 | 1.646584928 | 1.74% |
| 9 | -1545.133231 | 0.002823 | 1.771459319 | 1.41% |
| 10 | -1545.133135 | 0.002919 | 1.831700231 | 1.27% |
| 11 | -1545.132868 | 0.003186 | 1.999245267 | 0.96% |
| 12 | -1545.131999 | 0.004055 | 2.544551023 | 0.38% |
| 13 | -1545.131787 | 0.004267 | 2.677583037 | 0.30% |

Table S3. The coordinate for the lowest-energy conformer NMR calculations of the optimized 1R3R5S7S26S-**2**

| 1R3R5S7S26S-**2** Conf. 1 | | Standard Orientation (Ångstroms) | | |
| --- | --- | --- | --- | --- |
| I | atom | X | Y | Z |
| 1 | C | 0.654956 | 1.355753 | -0.12777 |
| 2 | C | -0.53756 | 0.957007 | -1.00127 |
| 3 | C | -1.39701 | -0.25049 | -0.60327 |
| 4 | C | -0.44818 | -1.3855 | -0.21342 |
| 5 | C | 0.472747 | -1.09485 | 0.973799 |
| 6 | C | 1.245238 | 0.20866 | 0.731328 |
| 7 | C | 0.172799 | 2.434449 | 0.944536 |
| 8 | C | 1.482336 | -2.28392 | 1.148189 |
| 9 | C | 2.563267 | -2.51655 | 0.085624 |
| 10 | O | 1.414917 | -3.02774 | 2.105297 |
| 11 | C | 1.825942 | 1.862712 | -1.032 |
| 12 | C | 2.353097 | 0.835425 | -2.0031 |
| 13 | C | 3.622218 | 0.423699 | -2.16183 |
| 14 | C | 3.982493 | -0.54114 | -3.26708 |
| 15 | C | 4.789259 | 0.874467 | -1.31721 |
| 16 | O | -0.84076 | 1.641492 | -1.96719 |
| 17 | C | -2.33728 | -0.64563 | -1.77216 |
| 18 | C | -3.33241 | -1.7315 | -1.4553 |
| 19 | C | -4.67219 | -1.63508 | -1.41435 |
| 20 | C | -5.51802 | -2.85298 | -1.12289 |
| 21 | C | -5.46508 | -0.37349 | -1.65651 |
| 22 | O | -0.42858 | -2.45027 | -0.80228 |
| 23 | C | 3.951048 | -2.37076 | 0.742715 |
| 24 | C | 2.403679 | -3.92586 | -0.51703 |
| 25 | C | -0.46835 | -0.83817 | 2.246124 |
| 26 | C | -1.38022 | 0.394613 | 1.942908 |
| 27 | C | -2.20861 | 0.199437 | 0.659449 |
| 28 | C | -0.75774 | 1.816577 | 2.052423 |
| 29 | O | 2.321176 | 0.377677 | 1.276999 |
| 30 | C | -0.48349 | 3.792621 | 0.446885 |
| 31 | C | -0.54014 | 4.761645 | 1.645288 |
| 32 | C | 0.233859 | 4.542212 | -0.69043 |
| 33 | O | -1.84935 | 3.579295 | 0.053945 |
| 34 | C | -1.37558 | -2.07098 | 2.492893 |
| 35 | C | 0.353716 | -0.60212 | 3.529559 |
| 36 | H | 1.115594 | 2.717151 | 1.427205 |
| 37 | H | 2.462948 | -1.76917 | -0.70815 |
| 38 | H | 1.464373 | 2.703359 | -1.61802 |
| 39 | H | 2.612802 | 2.230479 | -0.37237 |
| 40 | H | 1.612378 | 0.45101 | -2.70591 |
| 41 | H | 3.112103 | -0.82136 | -3.86846 |
| 42 | H | 4.734089 | -0.10418 | -3.93951 |
| 43 | H | 4.427466 | -1.45973 | -2.86031 |
| 44 | H | 5.468587 | 1.508896 | -1.90412 |
| 45 | H | 4.489058 | 1.427578 | -0.42533 |
| 46 | H | 5.378451 | 0.009389 | -0.98739 |
| 47 | H | -2.82952 | 0.266753 | -2.11316 |
| 48 | H | -1.70277 | -0.97902 | -2.6029 |
| 49 | H | -2.89205 | -2.70969 | -1.27185 |
| 50 | H | -4.90631 | -3.74398 | -0.95109 |
| 51 | H | -6.14904 | -2.69511 | -0.23684 |
| 52 | H | -6.20295 | -3.06579 | -1.95586 |
| 53 | H | -6.15188 | -0.50668 | -2.50393 |
| 54 | H | -6.09288 | -0.13838 | -0.78581 |
| 55 | H | -4.84368 | 0.50007 | -1.86566 |
| 56 | H | 4.735389 | -2.57429 | 0.0046 |
| 57 | H | 4.100917 | -1.36679 | 1.147003 |
| 58 | H | 4.054681 | -3.09228 | 1.559788 |
| 59 | H | 1.440744 | -4.03811 | -1.01981 |
| 60 | H | 2.47418 | -4.68137 | 0.27219 |
| 61 | H | 3.203508 | -4.11068 | -1.24331 |
| 62 | H | -2.11035 | 0.389639 | 2.764164 |
| 63 | H | -2.97339 | -0.56541 | 0.817006 |
| 64 | H | -2.73519 | 1.128584 | 0.4165 |
| 65 | H | -1.63044 | 2.46546 | 2.131457 |
| 66 | H | -0.241 | 1.885971 | 3.013696 |
| 67 | H | 0.471714 | 5.03291 | 1.96643 |
| 68 | H | -1.06203 | 5.672398 | 1.337099 |
| 69 | H | -1.07386 | 4.350496 | 2.504355 |
| 70 | H | 1.302765 | 4.67237 | -0.49392 |
| 71 | H | -0.21638 | 5.53602 | -0.78361 |
| 72 | H | 0.108666 | 4.035646 | -1.64977 |
| 73 | H | -1.83019 | 3.208308 | -0.84384 |
| 74 | H | -1.96583 | -2.35922 | 1.618301 |
| 75 | H | -2.07635 | -1.8401 | 3.304244 |
| 76 | H | -0.77648 | -2.93165 | 2.788037 |
| 77 | H | 1.117139 | 0.170582 | 3.416055 |
| 78 | H | -0.32531 | -0.29388 | 4.334312 |
| 79 | H | 0.85234 | -1.52141 | 3.837771 |

**Table S4. Conformers and Bolzmann distributions of the optimized 1R3R5S7S26R-2**

| species | *G* | *ΔG* | *ΔE(kcal/mol)* | *p%* |
| --- | --- | --- | --- | --- |
| 1 | -1545.133998 | 0 | 0 | 27.73% |
| 2 | -1545.133346 | 0.000652 | 0.409136194 | 13.89% |
| 3 | -1545.133247 | 0.000751 | 0.471259635 | 12.51% |
| 4 | -1545.132951 | 0.001047 | 0.657002446 | 9.14% |
| 5 | -1545.132908 | 0.00109 | 0.683985355 | 8.73% |
| 6 | -1545.132864 | 0.001134 | 0.711595773 | 8.34% |
| 7 | -1545.132705 | 0.001293 | 0.811369784 | 7.04% |
| 8 | -1545.132602 | 0.001396 | 0.876003262 | 6.32% |
| 9 | -1545.132469 | 0.001529 | 0.959462026 | 5.48% |
| 10 | -1545.130665 | 0.003333 | 2.091489164 | 0.81% |

**Table S5. The coordinate for the lowest-energy conformer NMR calculations of the optimized 1R3R5S7S26R-2**

| 1R3R5S7S26R-**2** Conf. 1 | | Standard Orientation (Ångstroms) | | |
| --- | --- | --- | --- | --- |
| I | atom | X | Y | Z |
| 1 | C | 0.929173 | 1.002252 | -0.5765 |
| 2 | C | -0.41875 | 0.875604 | -1.33167 |
| 3 | C | -1.62845 | 0.224137 | -0.6413 |
| 4 | C | -1.13424 | -1.05515 | 0.029239 |
| 5 | C | -0.05364 | -0.85966 | 1.093399 |
| 6 | C | 1.134929 | -0.06375 | 0.517423 |
| 7 | C | 0.810351 | 2.455613 | 0.090356 |
| 8 | C | 0.463717 | -2.26531 | 1.570306 |
| 9 | C | 1.234231 | -3.15883 | 0.58876 |
| 10 | O | 0.252294 | -2.66077 | 2.698782 |
| 11 | C | 2.064338 | 0.843484 | -1.6406 |
| 12 | C | 2.033728 | -0.4852 | -2.35564 |
| 13 | C | 3.013372 | -1.40197 | -2.43493 |
| 14 | C | 2.837058 | -2.64103 | -3.28168 |
| 15 | C | 4.353841 | -1.29872 | -1.74911 |
| 16 | O | -0.53905 | 1.380581 | -2.43128 |
| 17 | C | -2.74925 | -0.04687 | -1.67869 |
| 18 | C | -4.04454 | -0.55802 | -1.10371 |
| 19 | C | -5.24736 | 0.041345 | -1.11982 |
| 20 | C | -6.45507 | -0.65102 | -0.53163 |
| 21 | C | -5.5385 | 1.400193 | -1.7097 |
| 22 | O | -1.57205 | -2.15421 | -0.25642 |
| 23 | C | 2.647704 | -3.42492 | 1.145672 |
| 24 | C | 0.480769 | -4.49065 | 0.402902 |
| 25 | C | -0.71455 | 0.028388 | 2.252411 |
| 26 | C | -1.1486 | 1.409726 | 1.650465 |
| 27 | C | -2.10085 | 1.239972 | 0.452596 |
| 28 | C | -0.02913 | 2.458178 | 1.403656 |
| 29 | O | 2.246934 | -0.26961 | 0.970581 |
| 30 | C | 2.112893 | 3.325746 | 0.278386 |
| 31 | C | 3.18836 | 2.725172 | 1.188722 |
| 32 | C | 1.718775 | 4.723199 | 0.806736 |
| 33 | O | 2.763605 | 3.522625 | -0.9854 |
| 34 | C | -1.98147 | -0.67662 | 2.802796 |
| 35 | C | 0.251304 | 0.226338 | 3.439791 |
| 36 | H | 0.244757 | 3.027512 | -0.65788 |
| 37 | H | 1.319645 | -2.6531 | -0.37888 |
| 38 | H | 1.923787 | 1.636498 | -2.37783 |
| 39 | H | 3.02575 | 1.021959 | -1.16872 |
| 40 | H | 1.126598 | -0.67892 | -2.92899 |
| 41 | H | 3.611543 | -2.69775 | -4.05963 |
| 42 | H | 2.939661 | -3.55237 | -2.67616 |
| 43 | H | 1.860007 | -2.66685 | -3.77422 |
| 44 | H | 5.156461 | -1.16419 | -2.48805 |
| 45 | H | 4.40851 | -0.47803 | -1.03208 |
| 46 | H | 4.57969 | -2.22744 | -1.20935 |
| 47 | H | -2.89128 | 0.875162 | -2.24488 |
| 48 | H | -2.36236 | -0.78635 | -2.39089 |
| 49 | H | -3.98234 | -1.54828 | -0.65644 |
| 50 | H | -6.20096 | -1.62872 | -0.11076 |
| 51 | H | -6.91294 | -0.04489 | 0.262919 |
| 52 | H | -7.23359 | -0.80086 | -1.29302 |
| 53 | H | -6.2992 | 1.322252 | -2.49884 |
| 54 | H | -5.95536 | 2.072105 | -0.94635 |
| 55 | H | -4.6607 | 1.886337 | -2.14112 |
| 56 | H | 3.187752 | -4.10385 | 0.475684 |
| 57 | H | 3.2214 | -2.50092 | 1.246467 |
| 58 | H | 2.578199 | -3.89962 | 2.129963 |
| 59 | H | 0.373226 | -4.99785 | 1.3673 |
| 60 | H | 1.048409 | -5.14494 | -0.2686 |
| 61 | H | -0.51315 | -4.33151 | -0.01985 |
| 62 | H | -1.75071 | 1.86263 | 2.449931 |
| 63 | H | -3.07853 | 0.891457 | 0.795217 |
| 64 | H | -2.27584 | 2.207983 | -0.03437 |
| 65 | H | -0.51968 | 3.435746 | 1.456816 |
| 66 | H | 0.64972 | 2.428913 | 2.259642 |
| 67 | H | 2.823298 | 2.60408 | 2.213115 |
| 68 | H | 3.52503 | 1.750661 | 0.836907 |
| 69 | H | 4.044618 | 3.406647 | 1.212824 |
| 70 | H | 1.369119 | 4.710703 | 1.842977 |
| 71 | H | 0.930256 | 5.174081 | 0.188632 |
| 72 | H | 2.600603 | 5.368859 | 0.75201 |
| 73 | H | 2.138234 | 3.976417 | -1.57405 |
| 74 | H | -2.73366 | -0.88737 | 2.036962 |
| 75 | H | -2.44871 | -0.03179 | 3.556988 |
| 76 | H | -1.71379 | -1.62113 | 3.27515 |
| 77 | H | 0.365382 | -0.7057 | 3.993373 |
| 78 | H | 1.248878 | 0.551635 | 3.136669 |
| 79 | H | -0.16252 | 0.982083 | 4.119326 |


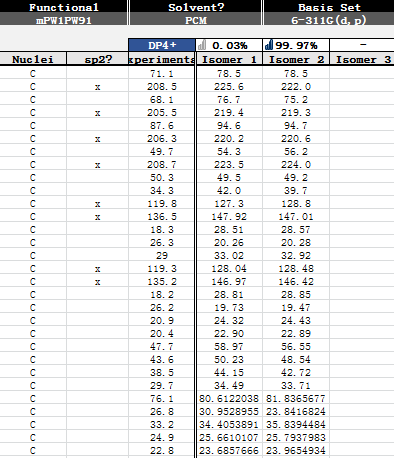


Figure S1. DP4+ analysis of compound **2** with isomer 1 (1R3R5S7S26S-**2**) and isomer 2 (1R3R5S7S26R-**2**).

S67. ECD calculation section

ECD Calculation Data of **4**


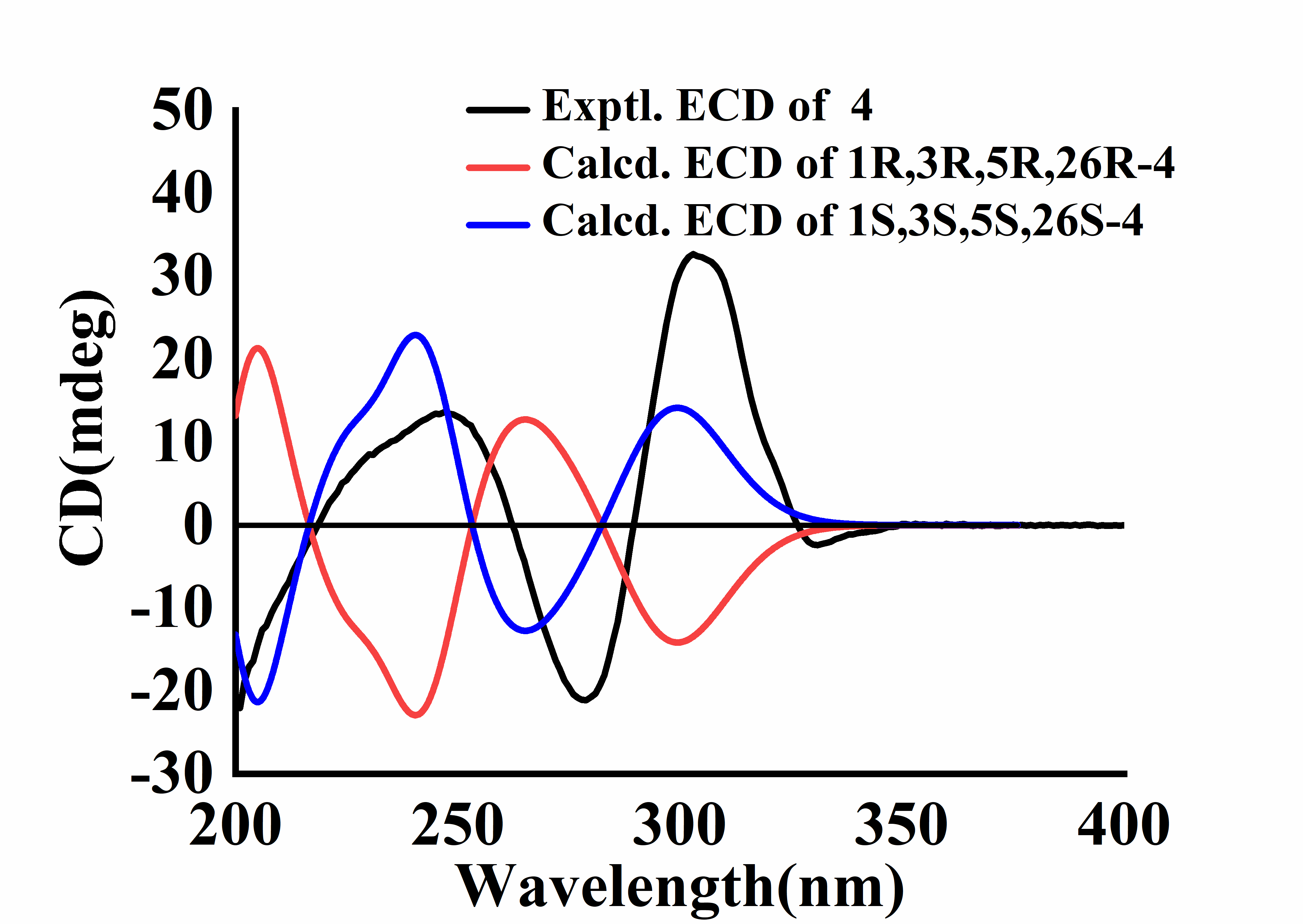


Figure S4. Experimental and calculated ECD spectra of **4**


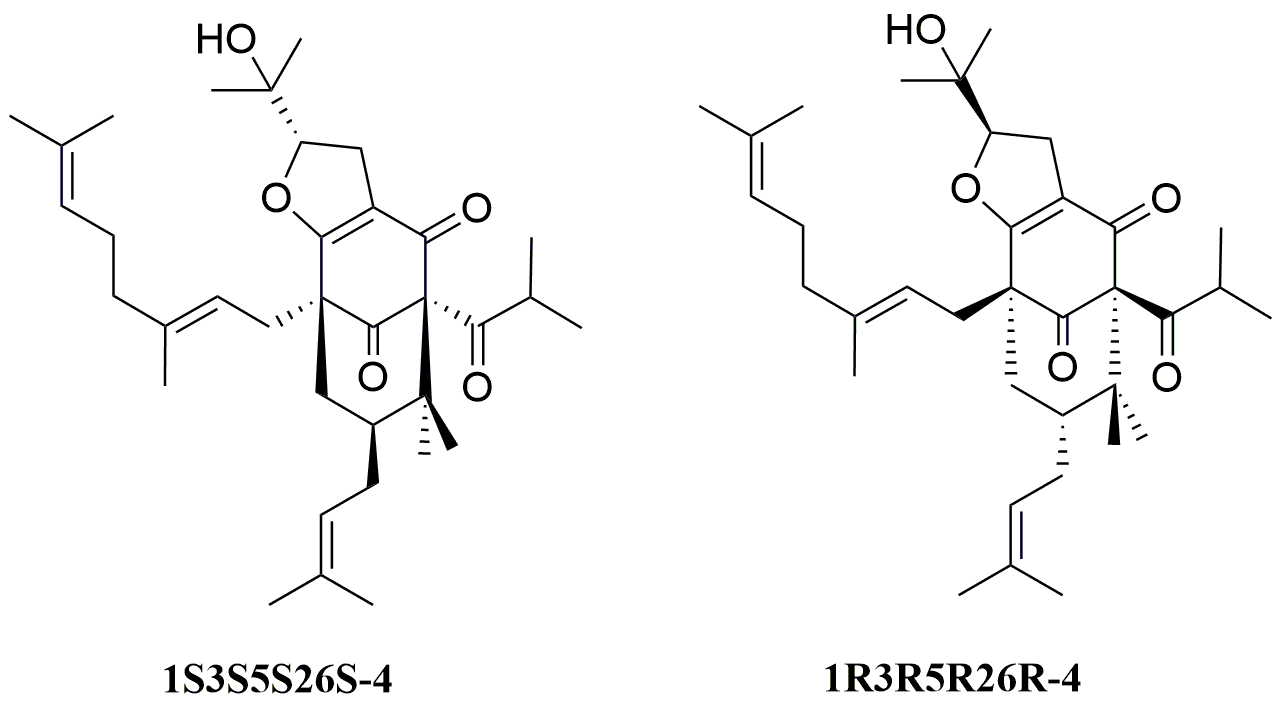


Table S1. Conformers and Bolzmann distributions of the optimized 1S3S5S26S-**4**

| species | *G* | *ΔG* | *ΔE(kcal/mol)* | *p%* |
| --- | --- | --- | --- | --- |
| 1 | -1233.25588 | 0 | 0 | 47.58% |
| 2 | -1233.255374 | 0.000506 | 0.317519807 | 27.83% |
| 3 | -1233.25421 | 0.001670 | 1.047940865 | 8.11% |
| 4 | -1233.25382 | 0.002060 | 1.29266957 | 5.36% |
| 5 | -1233.253818 | 0.002062 | 1.293924589 | 5.35% |
| 6 | -1233.253221 | 0.002659 | 1.66854776 | 2.84% |
| 7 | -1233.252764 | 0.003116 | 1.955319602 | 1.75% |
| 8 | -1233.252171 | 0.003709 | 2.327432735 | 0.93% |
| 9 | -1233.250918 | 0.004962 | 3.113702139 | 0.25% |

**Table S2. Cartesian coordinates of optimized 1S3S5S26S-4**

| 1S3S5S26S-**4** Conf.1 | | Standard Orientation (Ångstroms) | | |
| --- | --- | --- | --- | --- |
| I | atom | X | Y | Z |
| 1 | C | 0.026672 | -0.03768 | -1.28253 |
| 2 | C | -1.32305 | -0.51646 | -0.84255 |
| 3 | C | -1.06746 | -1.08498 | 0.561466 |
| 4 | C | -0.56598 | -0.05478 | 1.577494 |
| 5 | C | 0.593844 | 0.766359 | 0.940888 |
| 6 | C | 0.895694 | 0.55796 | -0.47929 |
| 7 | C | 2.084917 | 0.984206 | -1.23137 |
| 8 | C | 1.58034 | 0.777889 | -2.67516 |
| 9 | O | 0.417123 | -0.10889 | -2.61156 |
| 10 | C | 2.630407 | 0.18431 | -3.64432 |
| 11 | C | 3.86578 | 1.081011 | -3.74935 |
| 12 | C | 2.032543 | -0.03732 | -5.03871 |
| 13 | O | 1.299631 | 1.548999 | 1.57195 |
| 14 | C | -2.25928 | 0.706893 | -0.71382 |
| 15 | C | -1.87967 | 0.827866 | 1.891255 |
| 16 | C | -2.91658 | 0.814485 | 0.672531 |
| 17 | O | -1.29997 | -2.25543 | 0.856238 |
| 18 | C | -1.90756 | -1.58757 | -1.76559 |
| 19 | C | 0.086377 | -0.74075 | 2.811877 |
| 20 | C | -2.68783 | 0.24894 | 3.093009 |
| 21 | C | -3.91005 | 1.988275 | 0.65831 |
| 22 | C | 1.111519 | -1.85816 | 2.568726 |
| 23 | O | -0.10912 | -0.35112 | 3.966115 |
| 24 | C | -1.49186 | 2.273066 | 2.275797 |
| 25 | O | 3.064913 | -1.09112 | -3.15412 |
| 26 | C | 2.496498 | -1.40652 | 3.02639 |
| 27 | C | 0.683744 | -3.11771 | 3.317816 |
| 28 | H | 2.939348 | 0.348099 | -0.97975 |
| 29 | H | 2.357695 | 2.029216 | -1.05737 |
| 30 | H | 1.217588 | 1.736806 | -3.07294 |
| 31 | H | 4.379289 | 1.171225 | -2.78617 |
| 32 | H | 4.599131 | 0.652325 | -4.44236 |
| 33 | H | 3.604833 | 2.084557 | -4.10067 |
| 34 | H | 1.198701 | -0.7474 | -5.00881 |
| 35 | H | 2.776918 | -0.47275 | -5.71546 |
| 36 | H | 1.674847 | 0.900748 | -5.47542 |
| 37 | H | -3.04398 | 0.65066 | -1.48074 |
| 38 | H | -1.73084 | 1.647405 | -0.92363 |
| 39 | H | -3.53307 | -0.09166 | 0.779599 |
| 40 | H | -2.08551 | -1.1962 | -2.7737 |
| 41 | H | -1.23611 | -2.44969 | -1.85667 |
| 42 | H | -2.86384 | -1.95881 | -1.37912 |
| 43 | H | -2.23357 | 0.488084 | 4.058447 |
| 44 | H | -2.79442 | -0.8384 | 3.013679 |
| 45 | H | -3.69867 | 0.667785 | 3.14249 |
| 46 | H | -4.43753 | 2.089293 | 1.610714 |
| 47 | H | -4.67081 | 1.832752 | -0.11518 |
| 48 | H | -3.41142 | 2.937371 | 0.436357 |
| 49 | H | 1.169466 | -2.0857 | 1.500233 |
| 50 | H | -0.73987 | 2.282063 | 3.072436 |
| 51 | H | -1.094 | 2.825923 | 1.418588 |
| 52 | H | -2.35003 | 2.836425 | 2.658886 |
| 53 | H | 2.270641 | -1.65376 | -3.08673 |
| 54 | H | 2.505874 | -1.15518 | 4.092692 |
| 55 | H | 3.237383 | -2.19501 | 2.857417 |
| 56 | H | 2.822531 | -0.5181 | 2.47702 |
| 57 | H | 1.366643 | -3.94659 | 3.104359 |
| 58 | H | -0.32561 | -3.42709 | 3.028716 |
| 59 | H | 0.676771 | -2.95615 | 4.401585 |

Table S3. Conformers and Bolzmann distributions of the optimized 1R3R5R26R-**4**

| species | *G* | *ΔG* | *ΔE(kcal/mol)* | *p%* |
| --- | --- | --- | --- | --- |
| 1 | -1233.25588 | 0 | 0 | 47.58% |
| 2 | -1233.255374 | 0.000506000 | 0.317519807 | 27.83% |
| 3 | -1233.25421 | 0.001670000 | 1.047940865 | 8.11% |
| 4 | -1233.25382 | 0.002060000 | 1.29266957 | 5.36% |
| 5 | -1233.253817 | 0.002063000 | 1.294552098 | 5.34% |
| 6 | -1233.253221 | 0.002659000 | 1.66854776 | 2.84% |
| 7 | -1233.252764 | 0.003116000 | 1.955319602 | 1.75% |
| 8 | -1233.252171 | 0.003709000 | 2.327432735 | 0.93% |
| 9 | -1233.250918 | 0.004962000 | 3.113702139 | 0.25% |

**Table S4. Cartesian coordinates of optimized 1R3R5R26R-4**

| 1R3R5R26R-**4** Conf.2 | | Standard Orientation (Ångstroms) | | |
| --- | --- | --- | --- | --- |
| I | atom | X | Y | Z |
| 1 | C | 0.026672 | -0.03768 | -1.28253 |
| 2 | C | -1.32305 | -0.51646 | -0.84255 |
| 3 | C | -1.06746 | -1.08498 | 0.561466 |
| 4 | C | -0.56598 | -0.05478 | 1.577494 |
| 5 | C | 0.593844 | 0.766359 | 0.940888 |
| 6 | C | 0.895694 | 0.55796 | -0.47929 |
| 7 | C | 2.084917 | 0.984206 | -1.23137 |
| 8 | C | 1.58034 | 0.777889 | -2.67516 |
| 9 | O | 0.417123 | -0.10889 | -2.61156 |
| 10 | C | 2.630407 | 0.18431 | -3.64432 |
| 11 | C | 3.86578 | 1.081011 | -3.74935 |
| 12 | C | 2.032543 | -0.03732 | -5.03871 |
| 13 | O | 1.299631 | 1.548999 | 1.57195 |
| 14 | C | -2.25928 | 0.706893 | -0.71382 |
| 15 | C | -1.87967 | 0.827866 | 1.891255 |
| 16 | C | -2.91658 | 0.814485 | 0.672531 |
| 17 | O | -1.29997 | -2.25543 | 0.856238 |
| 18 | C | -1.90756 | -1.58757 | -1.76559 |
| 19 | C | 0.086377 | -0.74075 | 2.811877 |
| 20 | C | -2.68783 | 0.24894 | 3.093009 |
| 21 | C | -3.91005 | 1.988275 | 0.65831 |
| 22 | C | 1.111519 | -1.85816 | 2.568726 |
| 23 | O | -0.10912 | -0.35112 | 3.966115 |
| 24 | C | -1.49186 | 2.273066 | 2.275797 |
| 25 | O | 3.064913 | -1.09112 | -3.15412 |
| 26 | C | 2.496498 | -1.40652 | 3.02639 |
| 27 | C | 0.683744 | -3.11771 | 3.317816 |
| 28 | H | 2.939348 | 0.348099 | -0.97975 |
| 29 | H | 2.357695 | 2.029216 | -1.05737 |
| 30 | H | 1.217588 | 1.736806 | -3.07294 |
| 31 | H | 4.379289 | 1.171225 | -2.78617 |
| 32 | H | 4.599131 | 0.652325 | -4.44236 |
| 33 | H | 3.604833 | 2.084557 | -4.10067 |
| 34 | H | 1.198701 | -0.7474 | -5.00881 |
| 35 | H | 2.776918 | -0.47275 | -5.71546 |
| 36 | H | 1.674847 | 0.900748 | -5.47542 |
| 37 | H | -3.04398 | 0.65066 | -1.48074 |
| 38 | H | -1.73084 | 1.647405 | -0.92363 |
| 39 | H | -3.53307 | -0.09166 | 0.779599 |
| 40 | H | -2.08551 | -1.1962 | -2.7737 |
| 41 | H | -1.23611 | -2.44969 | -1.85667 |
| 42 | H | -2.86384 | -1.95881 | -1.37912 |
| 43 | H | -2.23357 | 0.488084 | 4.058447 |
| 44 | H | -2.79442 | -0.8384 | 3.013679 |
| 45 | H | -3.69867 | 0.667785 | 3.14249 |
| 46 | H | -4.43753 | 2.089293 | 1.610714 |
| 47 | H | -4.67081 | 1.832752 | -0.11518 |
| 48 | H | -3.41142 | 2.937371 | 0.436357 |
| 49 | H | 1.169466 | -2.0857 | 1.500233 |
| 50 | H | -0.73987 | 2.282063 | 3.072436 |
| 51 | H | -1.094 | 2.825923 | 1.418588 |
| 52 | H | -2.35003 | 2.836425 | 2.658886 |
| 53 | H | 2.270641 | -1.65376 | -3.08673 |
| 54 | H | 2.505874 | -1.15518 | 4.092692 |
| 55 | H | 3.237383 | -2.19501 | 2.857417 |
| 56 | H | 2.822531 | -0.5181 | 2.47702 |
| 57 | H | 1.366643 | -3.94659 | 3.104359 |
| 58 | H | -0.32561 | -3.42709 | 3.028716 |
| 59 | H | 0.676771 | -2.95615 | 4.401585 |

ECD Calculation Data of **5**


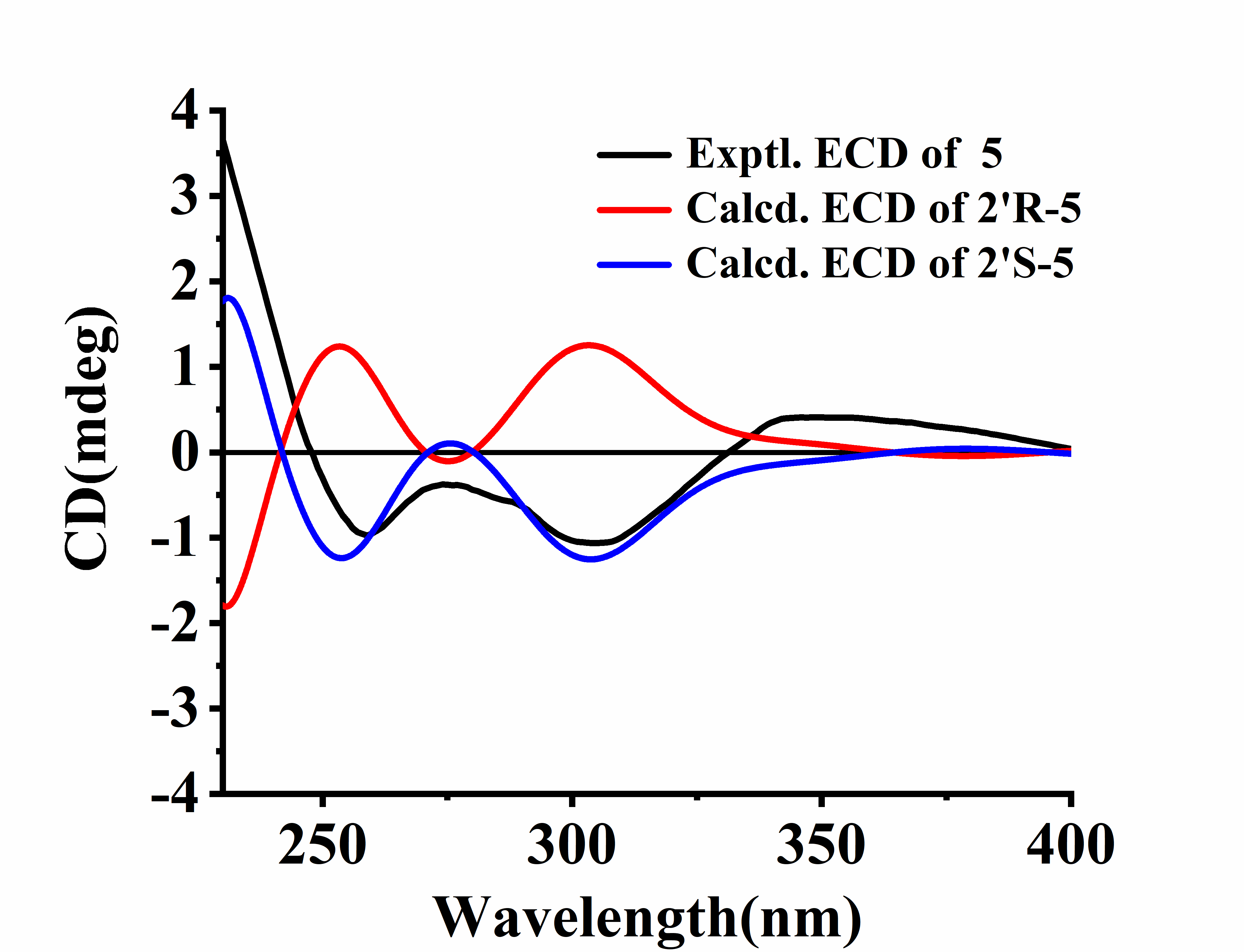


Figure S5. Experimental and calculated ECD spectra of **5**


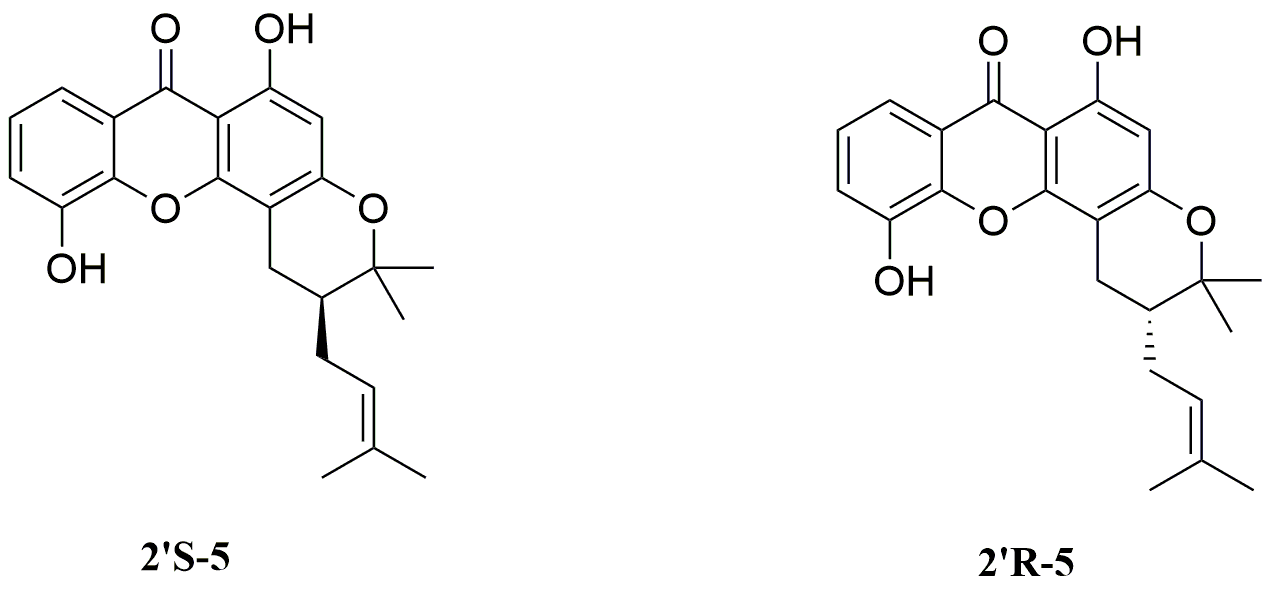


Table S1. Conformers and Bolzmann distributions of the optimized 2'S-**5**

| species | *G* | *ΔG* | *ΔE(kcal/mol)* | *p%* |
| --- | --- | --- | --- | --- |
| 1 | -1266.672391 | 0 | 0 | 63.45% |
| 2 | -1266.671625 | 0.000766000 | 0.480672194 | 28.16% |
| 3 | -1266.670339 | 0.002052000 | 1.28764927 | 7.20% |
| 4 | -1266.668326 | 0.004065000 | 2.550825674 | 0.85% |
| 5 | -1266.667153 | 0.005238000 | 3.28689419 | 0.25% |
| 6 | -1266.666182 | 0.006209000 | 3.896205809 | 0.09% |

**Table S2. Cartesian coordinates of optimized 2'S-5**

| 2'S-**5** Conf.1 | | Standard Orientation (Ångstroms) | | |
| --- | --- | --- | --- | --- |
| I | atom | X | Y | Z |
| 1 | C | 4.9918 | 2.2666 | 0.1296 |
| 2 | C | 3.8971 | 3.1288 | 0.2096 |
| 3 | C | 2.6063 | 2.6085 | 0.1854 |
| 4 | C | 2.3882 | 1.2337 | 0.0839 |
| 5 | C | 3.4846 | 0.3823 | 0.0034 |
| 6 | C | 4.7886 | 0.8893 | 0.0256 |
| 7 | O | 1.0844 | 0.8072 | 0.0711 |
| 8 | C | 0.83 | -0.5426 | -0.0347 |
| 9 | C | 1.8383 | -1.5048 | -0.1234 |
| 10 | C | 3.2385 | -1.0594 | -0.1063 |
| 11 | C | -0.5231 | -0.9316 | -0.0519 |
| 12 | C | -0.8364 | -2.2913 | -0.1291 |
| 13 | C | 0.174 | -3.2493 | -0.2311 |
| 14 | C | 1.5076 | -2.8577 | -0.2262 |
| 15 | O | 4.1539 | -1.8719 | -0.1816 |
| 16 | O | 1.563 | 3.4889 | 0.2629 |
| 17 | C | -1.6174 | 0.0938 | 0.0402 |
| 18 | C | -2.9833 | -0.4824 | -0.3632 |
| 19 | C | -3.1708 | -1.8858 | 0.2761 |
| 20 | O | -2.1117 | -2.7791 | -0.1324 |
| 21 | O | 2.4457 | -3.8497 | -0.3202 |
| 22 | C | -4.1163 | 0.5157 | -0.0401 |
| 23 | C | -3.9768 | 1.8184 | -0.7857 |
| 24 | C | -3.6665 | 3.0319 | -0.2883 |
| 25 | C | -3.5967 | 4.2344 | -1.195 |
| 26 | C | -3.3586 | 3.3328 | 1.1528 |
| 27 | C | -4.4529 | -2.5568 | -0.2493 |
| 28 | C | -3.2335 | -1.8817 | 1.8139 |
| 29 | H | -2.9548 | -0.6267 | -1.4547 |
| 30 | H | 6.0024 | 2.6683 | 0.1476 |
| 31 | H | 4.0492 | 4.2021 | 0.2896 |
| 32 | H | 5.642 | 0.2172 | -0.0378 |
| 33 | H | -0.0806 | -4.3034 | -0.3057 |
| 34 | H | 0.7414 | 2.9669 | 0.2116 |
| 35 | H | -1.6472 | 0.4792 | 1.0668 |
| 36 | H | -1.3754 | 0.9332 | -0.623 |
| 37 | H | 3.3357 | -3.4296 | -0.3008 |
| 38 | H | -5.0832 | 0.0932 | -0.3354 |
| 39 | H | -4.1826 | 0.6802 | 1.0385 |
| 40 | H | -4.1724 | 1.7352 | -1.8558 |
| 41 | H | -3.8302 | 3.9856 | -2.2356 |
| 42 | H | -4.3106 | 4.9973 | -0.8677 |
| 43 | H | -2.5907 | 4.6662 | -1.1746 |
| 44 | H | -3.3318 | 2.4455 | 1.7883 |
| 45 | H | -2.3772 | 3.8125 | 1.2337 |
| 46 | H | -4.1104 | 4.0146 | 1.5635 |
| 47 | H | -4.5008 | -3.6054 | 0.0691 |
| 48 | H | -5.3609 | -2.0591 | 0.1033 |
| 49 | H | -4.4641 | -2.5698 | -1.3453 |
| 50 | H | -3.3041 | -2.9069 | 2.1968 |
| 51 | H | -2.3297 | -1.4574 | 2.2621 |
| 52 | H | -4.0964 | -1.3221 | 2.1872 |

ECD Calculation Data of **6**


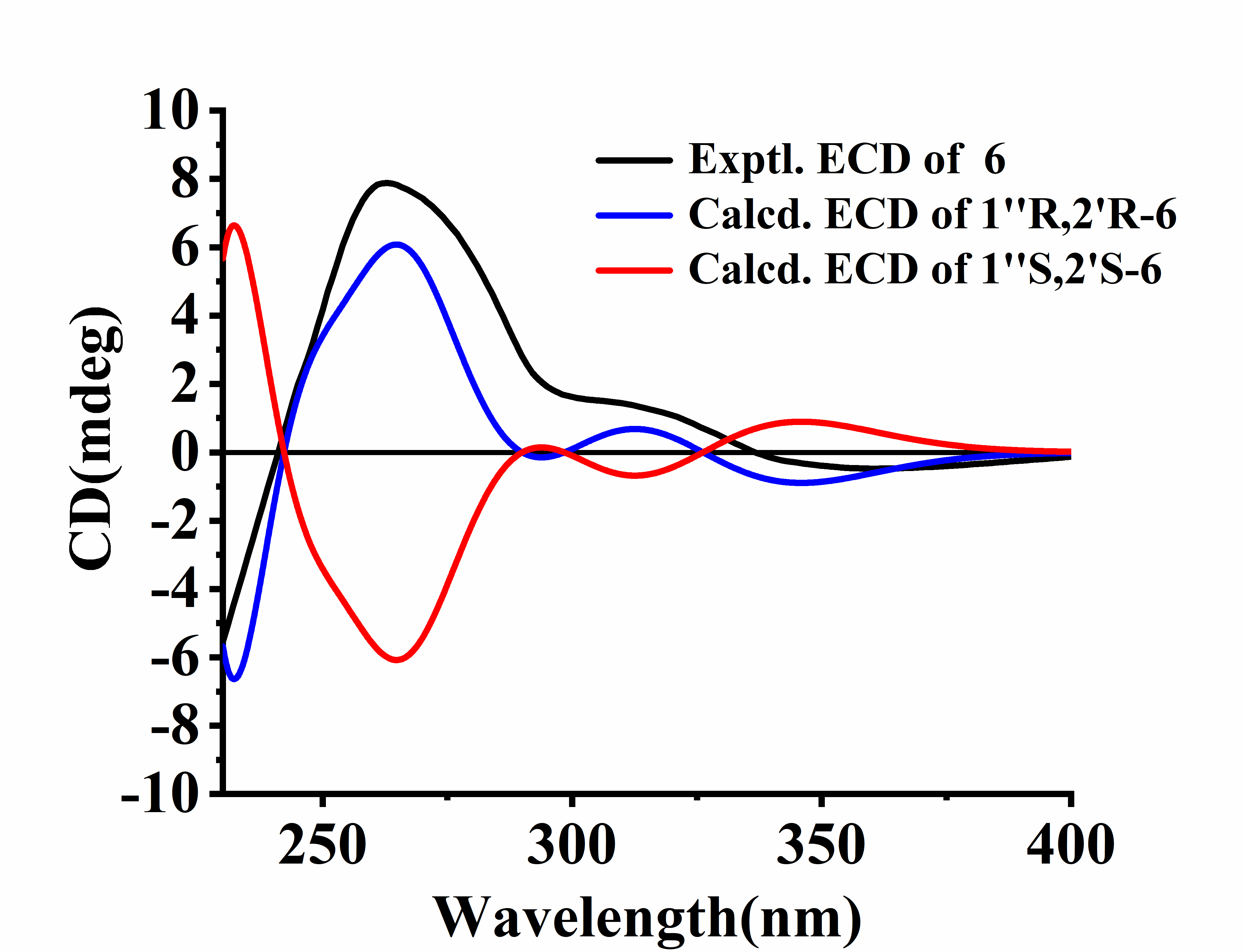


Figure S6. Experimental and calculated ECD spectra of **6**


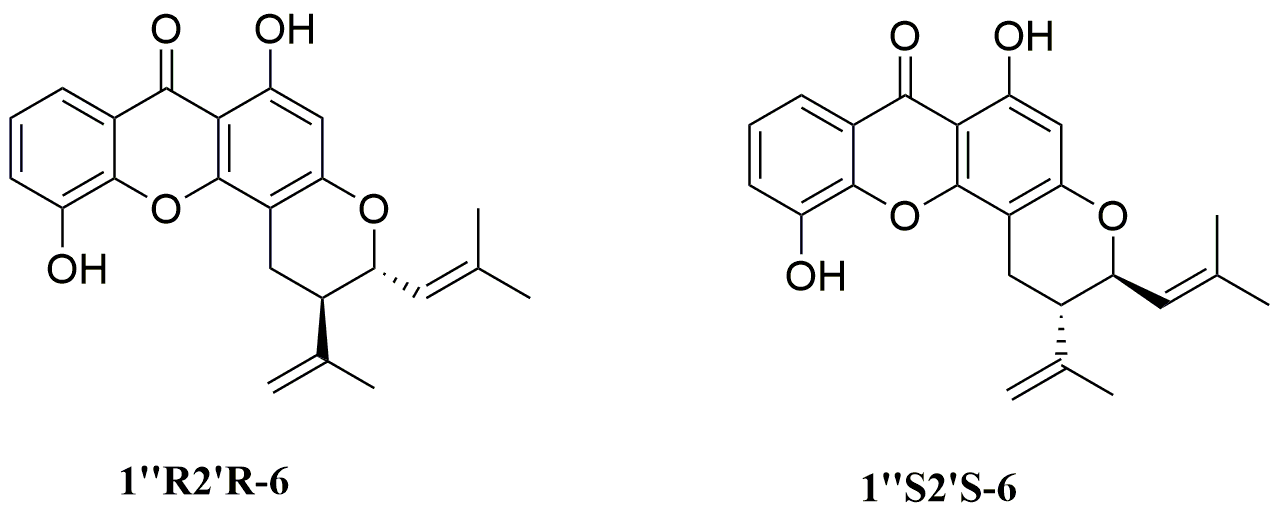


Table S1. Conformers and Bolzmann distributions of the optimized 1''R2'R-**6**

| species | *G* | *ΔG* | *ΔE(kcal/mol)* | *p%* |
| --- | --- | --- | --- | --- |
| 1 | -1265.460368 | 0 | 0 | 76.88% |
| 2 | -1265.459066 | 0.001302 | 0.817017227 | 19.33% |
| 3 | -1265.456225 | 0.004143 | 2.599771407 | 0.95% |
| 4 | -1265.456155 | 0.004213 | 2.643697064 | 0.88% |
| 5 | -1265.456008 | 0.00436 | 2.735940945 | 0.76% |
| 6 | -1265.455984 | 0.004384 | 2.75100117 | 0.74% |
| 7 | -1265.455547 | 0.004821 | 3.025222774 | 0.46% |

**Table S2. Cartesian coordinates of optimized 1''R2'R-6**

| 1''R2'R-**6** Conf.1 | | Standard Orientation (Ångstroms) | | |
| --- | --- | --- | --- | --- |
| I | atom | X | Y | Z |
| 1 | C | -5.9522 | -1.0878 | 0.2628 |
| 2 | C | -5.1898 | -2.2567 | 0.2556 |
| 3 | C | -3.8029 | -2.1754 | 0.167 |
| 4 | C | -3.159 | -0.9396 | 0.0859 |
| 5 | C | -3.9272 | 0.2195 | 0.0934 |
| 6 | C | -5.3223 | 0.1553 | 0.1814 |
| 7 | O | -1.7891 | -0.9508 | 0.0034 |
| 8 | C | -1.121 | 0.2512 | -0.081 |
| 9 | C | -1.7715 | 1.4877 | -0.0818 |
| 10 | C | -3.238 | 1.5109 | 0.0068 |
| 11 | C | 0.2829 | 0.1917 | -0.1696 |
| 12 | C | 1.0097 | 1.3873 | -0.2256 |
| 13 | C | 0.3549 | 2.6196 | -0.2406 |
| 14 | C | -1.0319 | 2.6699 | -0.1663 |
| 15 | O | -3.8475 | 2.5749 | 0.0076 |
| 16 | O | -3.0972 | -3.3462 | 0.1619 |
| 17 | O | -1.6081 | 3.9111 | -0.1766 |
| 18 | C | 1.0017 | -1.1318 | -0.1716 |
| 19 | C | 2.4562 | -0.9886 | -0.6476 |
| 20 | C | 3.0712 | 0.2396 | 0.0675 |
| 21 | O | 2.3736 | 1.4429 | -0.3055 |
| 22 | C | 4.508 | 0.4778 | -0.3377 |
| 23 | C | 3.2238 | -2.3011 | -0.4885 |
| 24 | C | 3.4652 | -2.83 | 0.9002 |
| 25 | C | 3.6832 | -2.9665 | -1.5629 |
| 26 | C | 5.6062 | 0.561 | 0.4407 |
| 27 | C | 6.9605 | 0.7991 | -0.1766 |
| 28 | C | 5.6281 | 0.4457 | 1.9392 |
| 29 | H | 2.4067 | -0.7421 | -1.7206 |
| 30 | H | 2.9788 | 0.1286 | 1.153 |
| 31 | H | -7.036 | -1.146 | 0.3314 |
| 32 | H | -5.6745 | -3.2276 | 0.3181 |
| 33 | H | -5.9166 | 1.0668 | 0.1867 |
| 34 | H | 0.9284 | 3.541 | -0.303 |
| 35 | H | -2.1541 | -3.1146 | 0.084 |
| 36 | H | -2.5837 | 3.7951 | -0.1161 |
| 37 | H | 0.9582 | -1.5293 | 0.8495 |
| 38 | H | 0.4828 | -1.8405 | -0.8294 |
| 39 | H | 4.6346 | 0.6004 | -1.4137 |
| 40 | H | 2.5211 | -3.0936 | 1.3855 |
| 41 | H | 4.0874 | -3.7317 | 0.8901 |
| 42 | H | 3.9804 | -2.0903 | 1.5182 |
| 43 | H | 4.236 | -3.8966 | -1.4645 |
| 44 | H | 3.5275 | -2.6002 | -2.5736 |
| 45 | H | 6.9139 | 0.8765 | -1.2679 |
| 46 | H | 7.3925 | 1.7303 | 0.2043 |
| 47 | H | 7.638 | -0.0254 | 0.0686 |
| 48 | H | 4.6387 | 0.3257 | 2.3842 |
| 49 | H | 6.2315 | -0.4171 | 2.24 |
| 50 | H | 6.0716 | 1.3457 | 2.3785 |
